# MSX2 Initiates and Accelerates Mesenchymal Stem/Stromal Cell Specification of hPSCs by Regulating TWIST1 and PRAME

**DOI:** 10.1016/j.stemcr.2018.06.019

**Published:** 2018-07-19

**Authors:** Leisheng Zhang, Hongtao Wang, Cuicui Liu, Qingqing Wu, Pei Su, Dan Wu, Jiaojiao Guo, Wen Zhou, Yuanfu Xu, Lihong Shi, Jiaxi Zhou

**Affiliations:** 1 State Key Laboratory of Experimental Hematology, Institute of Hematology & Blood Diseases Hospital, Chinese Academy of Medical Sciences & Peking Union Medical College, Tianjin 300020, China; 2 Center for Stem Cell Medicine, Chinese Academy of Medical Sciences & Department of Stem Cells and Regenerative Medicine, Peking Union Medical College, Tianjin 300020, China; 3 School of Basic Medical Science and Cancer Research Institute, Central South University, Changsha 410013, China

**Keywords:** human pluripotent stem cells, mesenchymal stem/stromal cells, MSX2, TWIST1

## Abstract

The gap in knowledge of the molecular mechanisms underlying differentiation of human pluripotent stem cells (hPSCs) into the mesenchymal cell lineages hinders the application of hPSCs for cell-based therapy. In this study, we identified a critical role of muscle segment homeobox 2 (MSX2) in initiating and accelerating the molecular program that leads to mesenchymal stem/stromal cell (MSC) differentiation from hPSCs. Genetic deletion of MSX2 impairs hPSC differentiation into MSCs. When aided with a cocktail of soluble molecules, MSX2 ectopic expression induces hPSCs to form nearly homogeneous and fully functional MSCs. Mechanistically, MSX2 induces hPSCs to form neural crest cells, an intermediate cell stage preceding MSCs, and further differentiation by regulating TWIST1 and PRAME. Furthermore, we found that MSX2 is also required for hPSC differentiation into MSCs through mesendoderm and trophoblast. Our findings provide novel mechanistic insights into lineage specification of hPSCs to MSCs and effective strategies for applications of stem cells for regenerative medicine.

## Highlights

• MSX2 rapidly initiates mesenchymal program in hPSCs
• Functional MSCs are derived with MSX2 programming and soluble factors
• Neural crest is the intermediate stage from hPSCs to MSCs with MSX2 programming
• MSX2 regulates mesengenesis by targeting TWIST1 and PRAME

## Teaser

In this article, Zhou and colleagues show that MSX2 rapidly initiates and accelerates MSC specification of hPSCs by regulating TWIST1 and PRAME. The study provides novel mechanistic insights into lineage specification of hPSCs to MSCs and effective strategies for applications of stem cells for regenerative medicine.

## Introduction

Mesenchymal stem/stromal cells (MSCs) are promising sources for cell-based therapies due to their self-renewal capacity, multi-lineage differentiation potential, and immunomodulatory properties (Friedenstein et al., 1968, Nombela-Arrieta et al., 2011). There are more than 300 clinical trials underway to evaluate the utility of MSCs in a variety of diseases, including autoimmune disorders, wound healing, and neurological disorders (Keating, 2012, Salem and Thiemermann, 2010). Currently, bone marrow-derived MSCs (BM-MSCs) are the most commonly used source for MSCs in clinical trials (Ankrum and Karp, 2010). However, these cell sources have some limitations, including limited cell proliferative capacity, declined therapeutic potency after *in vitro* expansion, donor-dependent variability in quality, and the risk of pathogen transmission (Wang et al., 2016). These shortcomings hamper their clinical applications. Therefore, there is an urgent need to find alternative inexhaustible sources of MSCs.

Human pluripotent stem cells (hPSCs), including human embryonic stem cells (hESCs) and human induced pluripotent stem cells (hiPSCs), have the capacity to self-renew indefinitely and give rise to almost all human cell types (Lund et al., 2012) and therefore have emerged as an alternative source for MSCs. Considerable progress has been made in differentiating hPSCs into MSCs with immune-phenotype and biological functions similar to those of BM-MSCs (Kimbrel et al., 2014, Wang et al., 2014). The use of hPSCs as a source for MSCs has many advantages, including generating unlimited amounts of early-passage MSCs with consistent high quality and deriving patient-derived induced pluripotent stem cells (iPSCs) for autologous therapy through gene correction (Frobel et al., 2014, Sabapathy and Kumar, 2016).

Since 2005, several groups have developed a number of protocols to differentiate hPSCs into MSCs with an immunophenotype and biological function similar to those of BM-MSCs. These methods include OP9 co-culture (Barberi et al., 2005, Olivier et al., 2006), three-dimensional embryoid body (EB) induction (Brown et al., 2009, Wei et al., 2012), and differentiation on two-dimensional monolayer (Gonzalo-Gil et al., 2016, Harkness et al., 2011). Despite these encouraging advances, limitations remain in the existing protocols. For example, most strategies require laborious manipulations, which include scraping, handpicking, sorting of cells, or serial passages (Fukuta et al., 2014, Gibson et al., 2017, Kopher et al., 2010, Lian et al., 2007, Sanchez et al., 2011). In addition, the current differentiation procedures are time consuming and usually take several weeks to obtain homogeneous MSCs (Boyd et al., 2009, Wang et al., 2016). Thus, the development of simple, rapid, and efficient methods directing the differentiation of hPSCs into MSCs becomes crucial.

In contrast to the advances in the development of differentiation strategies, little is known about the molecular signatures and mechanisms underlying the differentiation process (Deng et al., 2016, Luzzani and Miriuka, 2017). This can be largely attributed to the fact that most differentiation methods require several weeks to generate homogeneous MSCs from hPSCs, making it unfeasible to dissect the underlying molecular program. Recently, it was reported that inhibition of nuclear factor kappa B (NF-kB) signaling or EZH2 enhances differentiation of hPSCs to MSCs (Deng et al., 2016, Yu et al., 2017). Inhibition of transforming growth factor β (TGF-β) signaling with SB431542 also enhances the generation of MSCs (Fukuta et al., 2014, Mahmood et al., 2010). Besides these studies, little is known about the molecular mechanism for MSC differentiation. Thus, it is of great importance to establish an improved model for dissecting the molecular mechanism underlying hPSC differentiation toward MSCs. In this study, by combining MSX2 ectopic expression with a soluble-molecule (SM) cocktail, we developed a rapid and efficient strategy to generate near-homogeneity in MSCs from hPSCs within a week. The MSCs are functional and display multi-lineage differentiation potential and function in preventing colitis *in vivo* comparable with that of BM-MSCs. By conducting transcriptomic analysis, we uncovered multiple key signaling pathways and molecules involved in MSC differentiation from hPSCs. Furthermore, we identified TWIST1 and PRAME as crucial regulators of MSC differentiation.

## Results

### MSX2 Initiates Mesenchymal Differentiation in hPSCs

**Figure 1:**
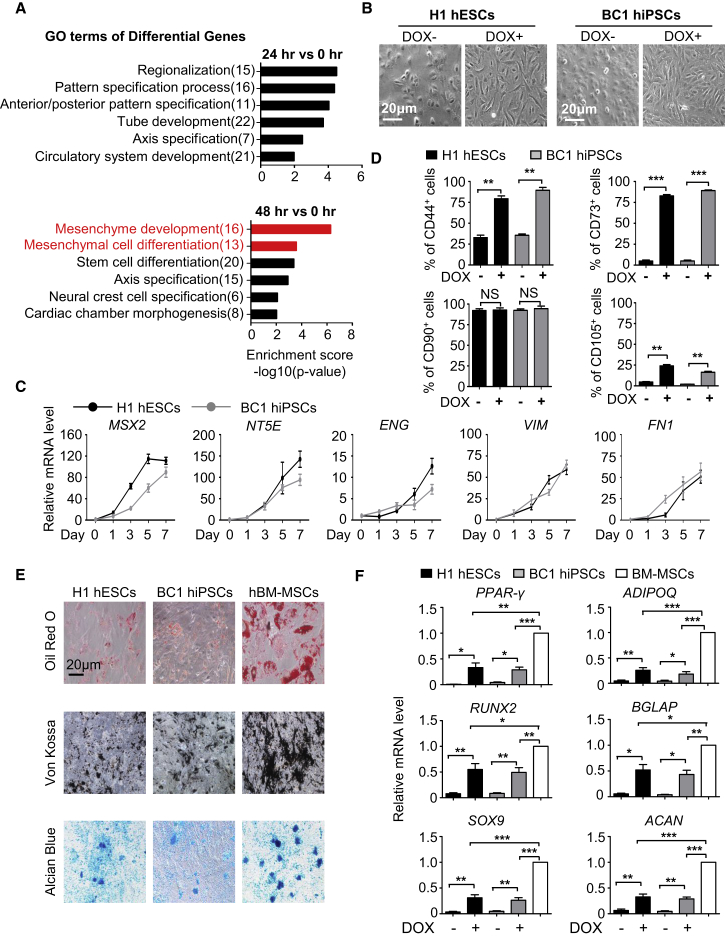
MSX2 Initiates Mesenchymal Differentiation in hPSCs (A) Gene ontology (GO) analysis of upregulated genes in DOX-inducible GFP-MSX2 H1 hESCs with 3 μg/mL DOX. (B) Images of GFP-MSX2 hPSCs (H1, BC1) cultured in DMEM/F12 containing 2%FBS ± DOX (3 μg/mL) for 7 days. Scale bar, 20 μm. (C) qRT-PCR analysis of MSX2 and MSC markers in GFP-MSX2 hPSCs (H1, BC1) with 3 μg/mL DOX (mean ± SEM, N = 3). Values are normalized to day 0 (=1) before adding DOX. (D) Flow cytometry (FCM) analysis of MSC markers of GFP-MSX2 hPSCs (H1, BC1) cultured in DMEM/F12 containing 2%FBS ± DOX (3 μg/mL) for 7 days (mean ± SEM, N = 3). ^∗∗^p < 0.01; ^∗∗∗^p < 0.001; NS, not significant. (E) Adipogenic, osteogenic, or chondrogenic differentiation potential of the MSCs derived from GFP-MSX2 hPSCs (H1, BC1) and human bone marrow-derived (hBM) MSCs for the indicated lineages. Scale bar, 20 μm. (F) qRT-PCR analysis of adipogenic (upper), osteogenic (middle), chondrogenic (bottom) markers after induction for the indicated lineages (mean ± SEM, N = 3). ^∗^p < 0.05; ^∗∗^p < 0.01; ^∗∗∗^p < 0.001. See also Figure S1.

We recently reported that MSX2 mediates the entry of hPSCs into mesendoderm during early fate specification (Wu et al., 2015). From the RNA sequencing (RNA-seq) data of hPSCs with MSX2 ectopic expression, we found rapid upregulation of multiple mesenchyme development and mesenchymal cell differentiation-associated genes in cells 48 hr and 72 hr after MSX2 overexpression, even under pluripotency-supporting conditions (Figures 1A and S1A). In contrast, early pattern specification and regionalization-associated genes were enriched mainly 24 hr after MSX2 overexpression (Figure 1A). These observations led us to speculate that MSX2 itself might be capable of initiating mesenchymal differentiation in hPSCs. To test this, we took advantage of a previously described DOX-inducible system to induce MSX2 ectopic expression under basal medium (DMEM/F12) containing 2% FBS, 1% L-glutamine (Gibco), and 1% non-essential amino acid (NEAA) (Gibco) known to support mesenchymal cells and then determined whether mesenchymal differentiation could be induced (Boyd et al., 2009). Overexpression of MSX2 in hPSCs (H1, H9, BC1, and Z-15), which could be monitored by the emergence of GFP fluorescence (Figure S1B), caused profound morphological changes, including from aggregates of cobblestone-shaped cells to separated cells with elongated, spindle-like shapes (Figures 1B and S1C), highly reminiscent of MSCs. To further characterize those cells, we measured the expression of CD44, CD73, CD90, CD105, and other markers of MSCs with qRT-PCR and flow cytometry assays (Barberi et al., 2005, Chen et al., 2012, Mani et al., 2008). Indeed, a gradual upregulation of NT5E (also known as CD73), ENG (also known as CD105), VIM, and FN1, and the concomitant sharp decrease of pluripotency markers, including POU5F1 (also known as OCT4), SOX2, and NANOG, were observed (Figures 1C and S1D). With MSX2 ectopic expression, almost 90% of cells were positive for CD44, CD73, and CD90 within 7 days of induction (Figures 1D, S1E, and S1F). In contrast, little expression of CD31, CD34, and CD45 was detected, indicating that the cells are neither endothelial nor hematopoietic cells (Figure S1E). Compared with MSCs from the bone marrow, CD105 expression levels were lower in MSX2 overexpressed cells (Figures 1D and S1F). This is an interesting distinction between MSX2-induced MSCs and those from the bone marrow (BM-MSCs).

We next determined the multi-lineage differentiation potential of the cells denoted as MSX2 programmed cells (M-MSCs), including to adipogenic, osteogenic, and chondrogenic cells (Zhang et al., 2017). After 3–4 weeks of differentiation, a portion of cells were stained positive for oil red O, von Kossa, and alcian blue, respectively (Figure 1E). However, compared with BM-MSCs, the differentiation potential of the MSX2-overexpressing cells was much lower. Experiments with qRT-PCR analysis of the multiple lineage differentiation markers (Vodyanik et al., 2010), including PPAR-γ, ADIPOQ, RUNX2, BGLAP, SOX9, and ACAN, further confirmed the observations (Figure 1F). Thus, although MSX2 ectopic expression suffices to induce entry of hPSCs to the mesenchymal fate, the differentiated cells appear to be immature and only exhibit some degree of adipogenic, osteogenic, and chondrogenic potential and partial expression of CD105 compared with BM-MSCs.

### Rapid and High-Efficiency Derivation of MSCs

**Figure 2:**
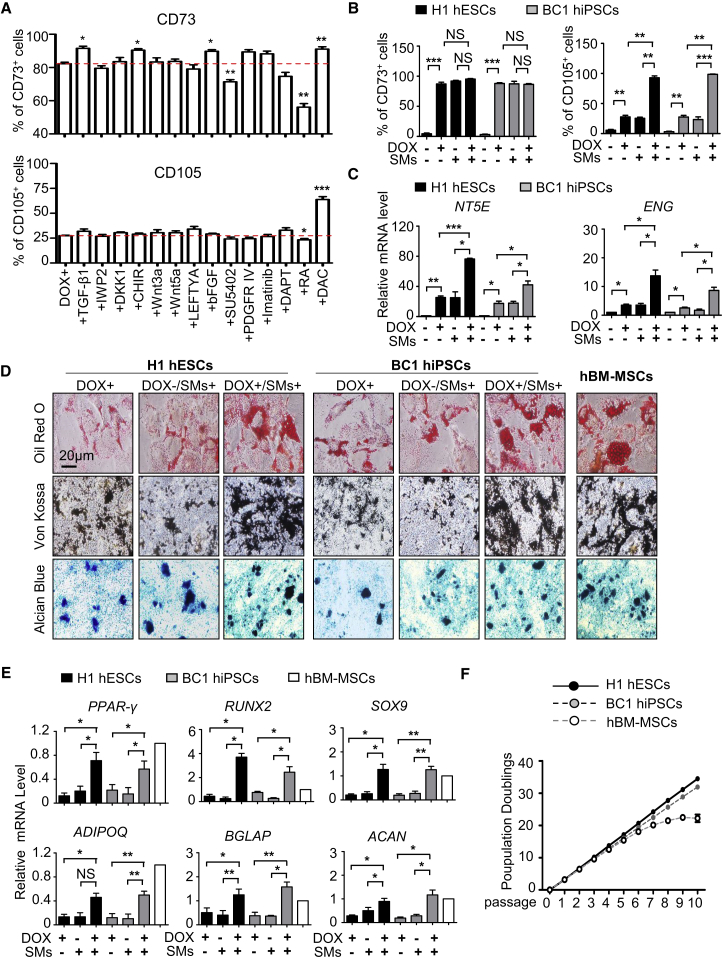
Rapid and High-Efficiency Derivation of MSCs (A) FCM analysis for MSC markers of GFP-MSX2 H1 hESCs with 3 μg/mL DOX alone or indicated chemical compound addition (see Table S3) for 7 days (mean ± SEM, N = 3). ^∗^p < 0.05; ^∗∗^p < 0.01; ^∗∗∗^p < 0.001. (B) FCM analysis of MSC markers in GFP-MSX2 hPSCs (H1, BC1) with indicated treatments for 7 days (mean ± SEM, N = 3). ^∗∗^p < 0.01; ^∗∗∗^p < 0.001; NS, not significant. (C) qRT-PCR analysis of MSC markers in GFP-MSX2 hPSCs (H1, BC1) with indicated treatments for 7 days (mean ± SEM, N = 3). ^∗^p < 0.05; ^∗∗^p < 0.01; ^∗∗∗^p < 0.001. Values are normalized to the Dox− group (=1). (D) Tri-lineage differentiation potential of MC-MSCs derived from GFP-MSX2 hPSCs (H1, BC1) and hBM-MSCs for the indicated lineages. Scale bar, 20 μm. (E) Relative expression levels of genes associated with tri-lineage differentiation of the MC-MSCs derived from GFP-MSX2 hPSCs (H1, BC1) (mean ± SEM, N = 3). ^∗^p < 0.05; ^∗∗^p < 0.01. Values are normalized to the hBM-MSCs group (=1). (F) Expansion potential of MC-MSCs derived from GFP-MSX2 hPSCs (H1, BC1) and hBM-MSCs in MSC culture media with CHIR99021 (0.5 μM) for 10 passages by population doubling assay (mean ± SEM, N = 3). See also Figure S2.

We and others have shown that chemical compounds are powerful tools for large-scale derivation of progenitor cells and terminal differentiated functional cells from hPSCs (Loh et al., 2016, Yang et al., 2016, Zhou et al., 2010). To identify conditions that allow for the development of more mature MSCs, we conducted a small-scale screening of chemical compounds and growth factors associated with the Wnt, fibroblast growth factor (FGF), TGF-βsignaling pathways as well as several known epigenetic regulators, all of which have been implicated in mesenchymal morphogenesis (Meng et al., 2013, Nieto et al., 2016, Thiery et al., 2009, Williams and Hare, 2011). The screening experiments allowed us to identify TGF-β1, CHIR99021, basic FGF (bFGF), and DAC (decitabine), a chemical compound that inhibits DNA methyltransferase, which enhanced the generation of CD44^+^, CD73^+^, or CD105^+^ cells when combined with MSX2 ectopic expression (Figures 2A and S2A–S2C). We subsequently applied all four SMs to MSX2-overexpressing cells (MC-MSCs) and found that the derived MC-MSCs showed typical elongated, spindle-like shapes more strikingly (Figure S2D). Moreover, CD73^+^ or CD105^+^ cells could be derived nearly homogeneously, much higher than MSX2 ectopic expression and growth factors/chemical compound treatment alone (Figures 2B and S2E–S2G). Consistent with these findings, the levels of NT5E, ENG, VIM, and FN1 mRNA were much higher in MC-MSCs (Figures 2C and S2H). In contrast, little difference of CD44, CD90, was detected between the different groups (Figure S2E). Furthermore, the colony-forming ability of MC-MSCs was much higher than that of M-MSCs and SM-MSCs, reaching almost the same level as BM-MSCs (Figure S2I).

We next examined the adipogenic, osteogenic, and chondrogenic potential of MC-MSCs. Indeed, the differentiation potential was significantly improved over the M-MSCs (Figure 2D), reaching the same level as BM-MSCs. Furthermore, elevated mRNA levels of adipogenic markers including PPAR-γ and ADIPOQ, osteogenic markers including RUNX2 and BGLAP, and chondrogenic markers including SOX9 and ACAN were also observed in MC-MSCs after differentiation into respective lineages (Figure 2E). Additionally, like BM-MSCs, MC-MSCs were capable of forming bone *in vivo* (Figure S2J). Importantly, unlike BM-MSCs, which have limited proliferation potential (Wei et al., 2012), the MC-MSCs could be cultivated consecutively for more than 10 passages (Figure 2F). In summary, MSX2 ectopic expression, aided with a cocktail of small molecules and soluble factors, allows us to accomplish rapid and near-homogeneous derivation of mature MSCs from hPSCs.

### MC-MSCs Resemble BM-MSCs and Are Functional

**Figure 3:**
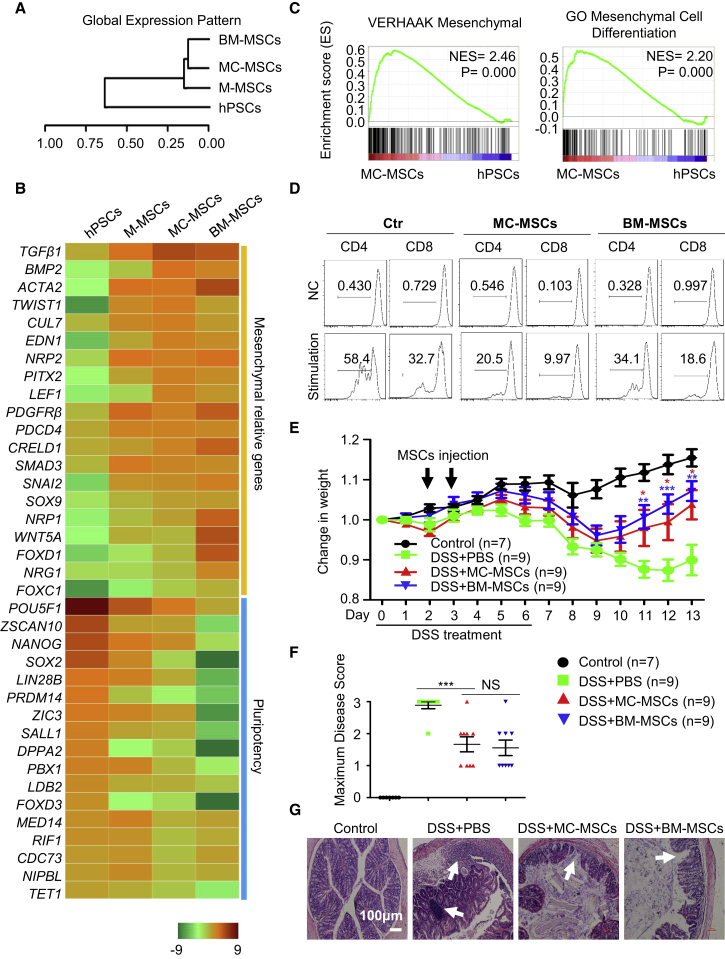
MC-MSCs Resemble BM-MSCs and Are Functional (A) Hierarchical clustering analysis of hPSCs (H1 hESCs), M-MSCs, MC-MSCs, BM-MSCs. (B) Heatmap illustrating expression of mesenchymal development/differentiation and pluripotency-associated genes for hPSCs (H1 hESCs), M-MSCs, MC-MSCs, BM-MSCs. (C) GSEA comparing MC-MSCs and hPSCs (H1 hESCs). The NES and p values are shown. (D) The sorted CD3^+^ T lymphocytes were stimulated with plate-bound anti-CD3 antibody and anti-CD28 antibody or with Molecular Probes sulfate latex for 72 hr. Then, the lymphocytes were stained with anti-CD4 or anti-CD8 antibodies for CFSE dilution analysis. One of three independent experiments is shown. Ctr, control. (E) Mice were given untreated drinking water (control) or 2% DSS in drinking water (DSS) for 6 days (Wang et al., 2016). Then, all mice were given untreated drinking water for the next 7 days. On days 2 and 3, mice treated with DSS were injected intraperitoneally (i.p.) with PBS, MC-MSCs or BM-MSCs. The control group mice were injected i.p. with PBS. The change in body weight of mice was measured. Data are analyzed by multiple t test and shown as mean ± SEM (N = 3). ^∗^p < 0.05; ^∗∗^p < 0.01, ^∗∗∗^p < 0.001. (F) The maximum colitis severity of each animal in the four groups was quantified by maximum disease score, which is typically assessed based on the stool consistency, rectal bleeding, and body weight together (Wang et al., 2016). Data are analyzed by students' t test and shown as mean ± SEM. ^∗∗∗^p < 0.001; NS, not significant. (G) Histopathologic analysis of colons by H&E staining. The white arrows indicate typical morphology of the damage. Scale bar, 100 μm. See also Figure S3.

To further characterize MC-MSCs at a molecular level, we conducted genome-wide RNA profiling to compare MC-MSCs with BM-MSCs. Unsupervised clustering revealed grouping of MC-MSCs with BM-MSCs (Figure 3A). Furthermore, MC-MSCs have much more similarities in global gene expression to BM-MSCs as opposed to M-MSCs or hPSCs (Figure 3B). Expectedly, multiple pluripotency-associated genes showed minimal expression in MC-MSCs, while mesenchymal development/differentiation-associated genes were highly expressed with levels comparable with those in BM-MSCs (Figure 3B). Gene set enrichment analysis (GSEA) also showed high enrichment of genes, including mesenchymal cell markers and genes involved in mesenchymal cell differentiation, mesenchyme development, and positive regulation of mesenchymal cell proliferation in MC-MSCs (Figures 3C and S3A).

The similarities between MC-MSCs and BM-MSCs led us to ask whether MC-MSCs are functional. We first examined whether MC-MSCs exhibit immunomodulatory activity *in vitro*. After interferon γ treatment for 24 hr, the expression of anti-inflammatory gene IDO1 and pre-inflammatory gene IL-6 dramatically elevated in both MC-MSCs and BM-MSCs, while TGF-β expression was mostly affected (Figure S3B). Furthermore, like BM-MSCs, MC-MSCs inhibited proliferation of CD4^+^ T lymphocytes when stimulated with anti-CD3 and anti-CD28 antibody and CD8^+^ T lymphocytes when stimulated with Molecular Probes sulfate latex (Figure 3D). To further explore the immunomodulatory activity of MC-MSCs *in vivo*, we took advantage of a recently described dextran sulfate sodium (DSS)-induced acute colitis model (Wang et al., 2016) to assess whether colitis-caused tissue damage and decrease in body weight could be treated with MC-MSCs. In this model, the decrease of body weight occurred in the window between day 4 and day 9 after DSS treatment, and the mice soon began to gain weight from day 10 after application of MC-MSCs or BM-MSCs (Figure 3E). The maximum weight loss measurement also showed significant improvement after MC-MSC treatment (Figure S3C, p < 0.05). No significant difference was found between the groups of MC-MSCs or BM-MSCs. The maximum disease score was much lower after MC-MSC treatment (Figure 3F, p < 0.001). Also, application of MC-MSCs prevented the decrease of colon length (Figures S3D and S3E, p < 0.001), while no significant difference was seen between animals treated with MC-MSCs or BM-MSCs. Compared with the DSS + PBS group, MC-MSCs reduced the maximum severity of DSS-induced colitis (Figure 3F). As expected, less damage to the epithelial cells was also observed after MC-MSC treatment (Figure 3G). Thus, MC-MSCs exhibit healing effects similar to BM-MSCs in the *in vivo* colitis model and therefore can be potentially used as tools for cell-based therapy and other regenerative medicine-related purposes.

### Neural Crest as the Intermediate Stage between Pluripotency and Mesenchymal Fate

**Figure 4:**
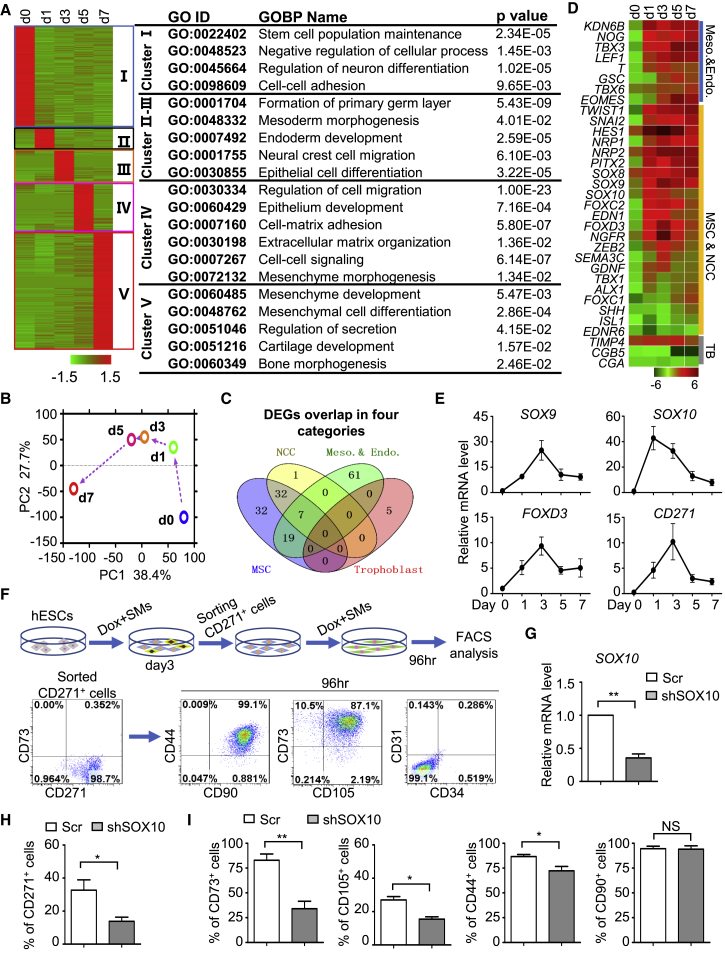
Neural Crest as the Intermediate Stage between Pluripotency and Mesenchymal Fate (A) Hierarchical cluster analysis of 2,782 differentially expressed genes (DEGs) (fold change >2, fragments per kilobase of transcript per million mapped reads >0.2) in whole-transcriptome level (left) and GO biological process (GOBP) analysis with p value (right). (B) PCA of samples of MC-MSC induction from GFP-MSX2 H1 hESCs. PC1, principal component 1; PC2, principal component 2. (C) Venn diagram shows the overlap among MSC, NCC, mesoderm and endoderm, and trophoblast-associated genes enriched in 14,453 DEGs. (D) Heatmap illustration shows the expression changes of mesendoderm, MSC and NCC, and TB (trophoblast)-associated genes during MC-MSC induction. (E) qRT-PCR analysis of the dynamic expression for NCC-associated genes during MSC induction from GFP-MSX2 H1 hESCs with DOX + SM (mean ± SEM, N = 3). (F) MSC potential analysis of CD271^+^ NCCs derived from GFP-MSX2 H1 hESCs. CD271^+^ cells were isolated at day 3 of differentiation and cultured with DOX + SM for 96 hr, followed by FCM analysis. (G) qRT-PCR analysis showing the depletion of SOX10 by small hairpin RNAs (shRNAs; a mixture of shSOX10-1, shSOX10-2) (mean ± SEM, N = 3). ^∗∗^p < 0.01. (H and I) FCM analysis for CD271^+^ NCCs at day 3 (H) or MSC markers at day 7 (I) in H1 hESCs (Scr, shSOX10) under SMs conditions, respectively (mean ± SEM, N = 3). ^∗^p < 0.05; ^∗∗^p < 0.01; NS, not significant. See also Figure S4.

The high-efficiency and rapid directed differentiation method we have developed makes it feasible to dissect how cell fate changes occur from the pluripotent state to the mesenchymal fate. We conducted time course RNA-seq analysis of cells undergoing the fate changes (i.e., from day 0 to day 7). To elucidate the differentiation route, 2,782 highly expressed genes at different time points were clustered by using hierarchical cluster analysis (Figure 4A). Consistent with the initial loss of pluripotency and onset of early differentiation, genes associated with primary germ layer formation were highly enriched in the cell population of day 1 (Figure 4A). Overall, the principal component analysis (PCA) of the transcriptome showed a clear stepwise differentiation process from pluripotency to mesenchymal cells (Figure 4B).

Where do mesenchymal cells originate during early human development? There have been different reports suggesting that they might arise from mesoderm, endoderm, or the neural crest based on *in vivo* or *in vitro* models (Fukuta et al., 2014, Motohashi et al., 2007, Slukvin and Vodyanik, 2011, Vodyanik et al., 2010). Recently, trophoblasts were reported as the potential origin for MSCs during hPSC differentiation (Wang et al., 2016). To explore this in our differentiation model, we clustered those 14,453 differentially expressed genes into different categories and tested their potential overlaps using Venn map analysis. Surprisingly, no overlap of MSC-associated genes with trophoblasts was found (0/5) (Figure 4C). In contrast, neural crest-associated genes exhibited nearly perfect overlap with those of MSCs (39/40) (Figure 4C), suggesting that the neural crest may be the main intermediate stage during MSC induction in our method. To test this, we explored the dynamic expression of neural crest-related genes during MSC induction. The results from RNA-seq showed that neural crest-associated genes quickly upregulated at day 1 of MSC induction, peaked at day 3, and then began to decrease (Figure 4D). Real-time PCR analysis of the neural crest-related genes, including SOX9, SOX10, FOXD3, and CD271, further confirmed these observations (Figure 4E). Consistently, flow cytometry analysis showed that CD271^+^ cells began to appear at day 1 of MSC induction, peaked at day 3, became CD73^+^ cells at day 5, and disappeared at day 7 (Figure S4A). Importantly, the isolated CD271-positive cells at day 3 of MSC induction could further differentiate to MSCs (Figure 4F). Furthermore, knockdown of SOX10, a master regulator of neural crest genesis (Gammill and Bronner-Fraser, 2003), severely impaired neural crest induction and MSC induction (Figures 4G–4I), strongly indicating that neural crest serves as the main intermediate stage during the cell fate transition from pluripotency to mesenchymal cells. Interestingly, we noticed that the isolated CD271^−^ cells could also partially differentiate into MSCs (Figure S4B). The increase of mesendoderm-associated genes T and MIXL1 was observed (Figures 4D and S4C), although their expression was much lower than those of neural crest-related genes, and these data implied that mesendoderm may act as another potential origin for MSC differentiation. Together, these data suggest that neural crest serves as the main intermediate cell type during MSC induction with our current methods (Figures S4D–S4H).

### MSX2 Is Critical for Mesenchymal Differentiation

**Figure 5:**
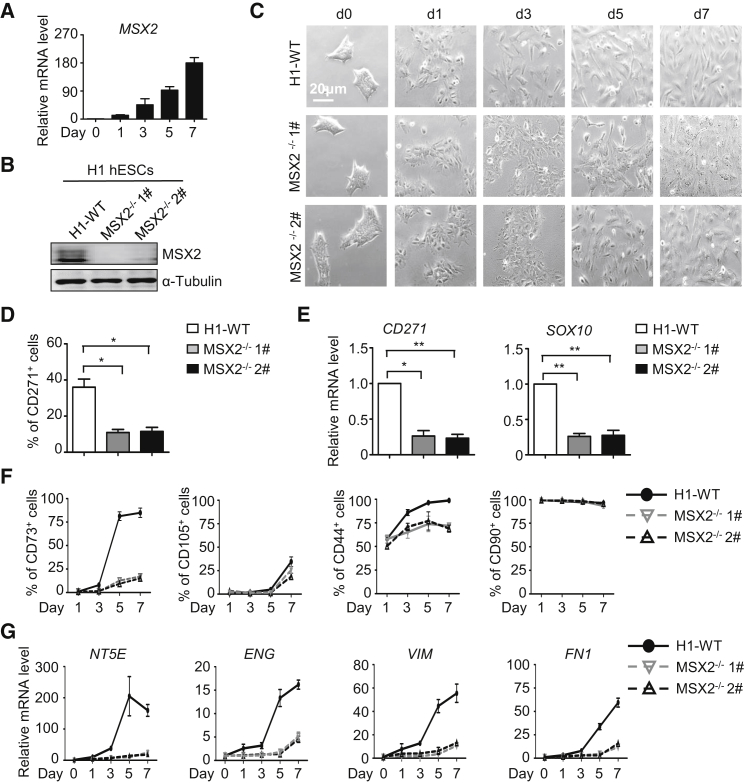
MSX2 Is Critical for Mesenchymal Differentiation (A) Time course analysis of MSX2 gene expression in H1 hESCs under SMs conditions for indicated time by qRT-PCR (mean ± SEM, N = 3). (B) Western blotting analysis confirmed the expression of MSX2 in wild-type H1 (H1-WT) and MSX2-deleted H1 hESCs after MSC induction for 7 days under SMs conditions. α-Tubulin was used as a loading control. (C) Images of H1-WT and MSX2-deleted H1 hESCs under SMs conditions for indicated time points. Scale bar, 20 μm. (D and E) FCM analysis (D) and qRT-PCR analysis (E) for NCC marker in H1-WT and MSX2-deleted H1 hESCs after MSC induction at day 3 under SMs conditions (mean ± SEM, N = 3). ^∗^p < 0.05; ^∗∗^p < 0.01. (F and G) FCM analysis (F) and qRT-PCR analysis (G) for MSC markers for indicated times in H1-WT and MSX2-deleted H1 hESCs after MSC induction under SMs conditions (mean ± SEM, N = 3).

Because overexpression of MSX2 sufficed to initiate the MSC differentiation from hPSCs (Figures 1B–1F), we asked whether MSX2 is necessary for human mesengenesis. The use of small molecules, albeit not optimal, already enabled us to achieve consistent generation of CD73^+^ and CD105^+^ MSCs (Figures 2A–2C). Indeed, upregulation of endogenous MSX2 was observed in this differentiation system (Figure 5A). We next assessed the effect of MSX2 deletion in H1 hESCs (established previously; Wu et al., 2015) and BC1 hiPSCs (established herein) on mesenchymal differentiation. First, MSX2 deletion significantly delayed the morphological changes of hPSCs to MSCs (Figures 5B and S5A). On day 3, while all wild-type cells became elongated and turned into spindle-like separated cells, MSX2-deleted cells still retained the morphology of cell aggregates (Figure 5C). Consistently, compared with the wild-type cells, neural crest cell (NCC) generation was severely impaired in MSX2-deleted cells (Figures 5D and 5E). Furthermore, much lower percentages of CD73^+^ and CD105^+^ cells were produced with MSX2 deletion compared with wild-type H1 hESCs and BC1 hiPSCs (Figures 5F and S5B). Consistently, expressions of NT5E, ENG, VIM, and FN1 were also severely attenuated after MSX2 deletion (Figures 5G and S5C). Thus, MSX2 is essential for MSC differentiation of hPSCs.

As mentioned above, mesendoderm and trophoblasts were also reported as the alternative origins for MSCs during hPSC differentiation. We thus also tested whether MSX2 deletion had any impact on hPSC differentiation into MSCs through mesendoderm and trophoblast by taking advantage of previously reported strategies (Tran et al., 2012, Wang et al., 2016). Indeed, we found MSX2 deletion severely decreased the expression of mesendoderm and trophoblast-associated genes (Figures S5D and S5E) and the percentage of CD44^+^, CD73^+^, and CD105^+^ cells (Figures S5F and S5G). Markers of mesenchymal cells, such as NT5E, ENG, VIM, and FN1, were also found to be much lower after MSX2 depletion (Figures S5H and S5I). Thus, MSX2 is also required for hPSC differentiation into MSCs through mesendoderm and trophoblast. Altogether, these data revealed MSX2 is a general effector mediating MSC differentiation from hPSCs.

### PRAME and TWIST1 Are Essential for MSC Generation of hPSCs

**Figure 6:**
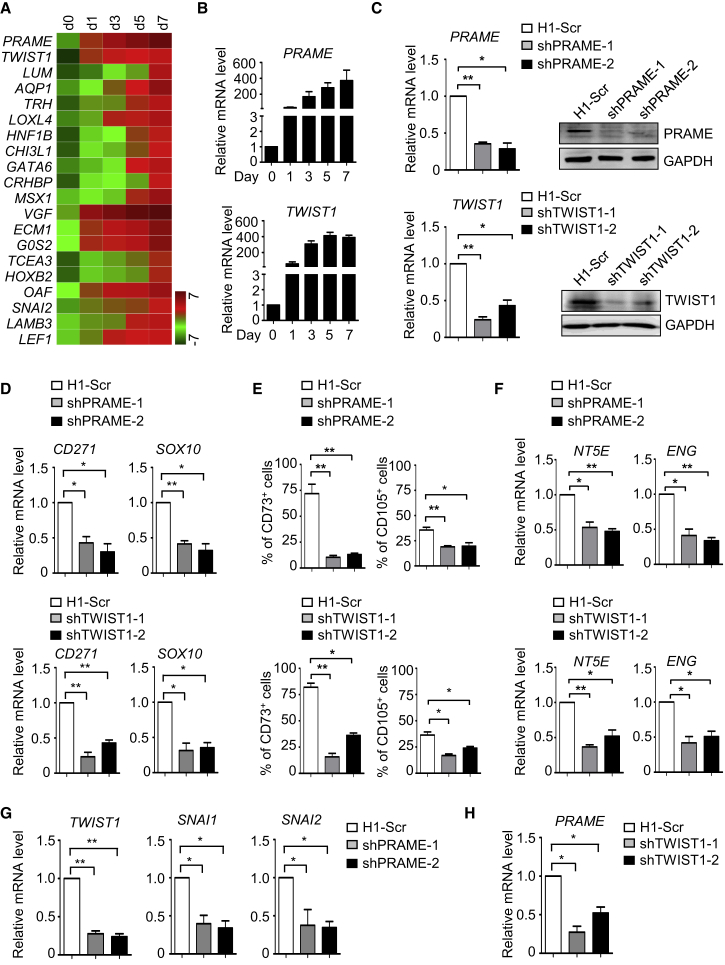
PRAME and TWIST1 Are Essential for MSC Generation from hPSCs (A) Heatmap illustration shows top 20 DEGs continuously upregulated according to the fold change during hPSC-MSC induction with DOX + SMs. (B) Time course analysis of PRAME and TWIST1 mRNA levels in GFP-MSX2 H1 hESCs during MC-MSC induction by qRT-PCR (mean ± SEM, N = 3). Values are normalized to day 0 (=1) before adding DOX. (C) qRT-PCR and western blotting analysis showing the successful depletion of PRAME or TWIST1 by shRNA targeting PRAME or TWIST1 (mean ± SEM, N = 3). ^∗^p < 0.05; ^∗∗^p < 0.01. (D) qRT-PCR analysis of NCC markers in H1 hESCs (Scr, shPRAME, shTWIST1) after MSC induction for 3 days under SMs conditions (mean ± SEM, N = 3). ^∗^p < 0.05; ^∗∗^p < 0.01. (E) FCM analysis for MSC markers in H1 hESCs (Scr, shPRAME, shTWIST1) after MSC induction for 7 days under SMs conditions (mean ± SEM, N = 3). ^∗^p < 0.05; ^∗∗^p < 0.01. (F) qRT-PCR analysis of MSC markers in H1 hESCs (Scr, shPRAME, shTWIST1) after MSC induction for 7 days under SMs conditions (mean ± SEM, N = 3). ^∗^p < 0.05; ^∗∗^p < 0.01. (G and H) qRT-PCR analysis of TWIST1-associated genes or PRAME in H1 hESCs (Scr, shPRAME, shTWIST1) after MSC induction for 3 days under SMs conditions (mean ± SEM, N = 3). ^∗^p < 0.05; ^∗∗^p < 0.01. See also Figure S6.

To further dissect the molecular mechanisms underlying human mesenchymal differentiation, we sought to identify genes essential for the differentiation process. First, we selected the top 20 genes with consecutive upregulation during differentiation (Figure 6A), allowing us to discover PRAME (preferentially expressed antigen in melanoma) (Epping et al., 2005) and TWIST1 (twist family bHLH transcription factor 1) (Qin et al., 2012), both of which exhibited robust and rapid upregulation at the early stage of MSC differentiation from hPSCs under the above optimal or sub-optimal circumstances (Figures 6B and S6A). We next asked whether they had a functional role in mesenchymal differentiation. Small hairpin RNA-mediated knockdown successfully depleted PRAME or TWIST1, as shown by decreased mRNA and protein levels (Figure 6C). Indeed, depletion of PRAME or TWIST1 severely impaired the differentiation of neural crest, as assessed by real-time PCR analysis of neural crest-associated genes CD271 and SOX10 (Figure 6D). Furthermore, the percentage of CD44^+^, CD73^+^, and CD105^+^ cells (Figures 6E and S6B) and mRNA levels of NT5E, ENG, VIM, and FN1 were significantly reduced (Figures 6F and S6C). Interestingly, we also found mutual regulation between TWIST1 and PRAME during MSC induction (Figures 6G and 6H). Thus, PRAME and TWIST1 are critical for hPSC differentiation to MSCs.

### MSX2 Directly Targets TWIST1 during Mesenchymal Differentiation

**Figure 7:**
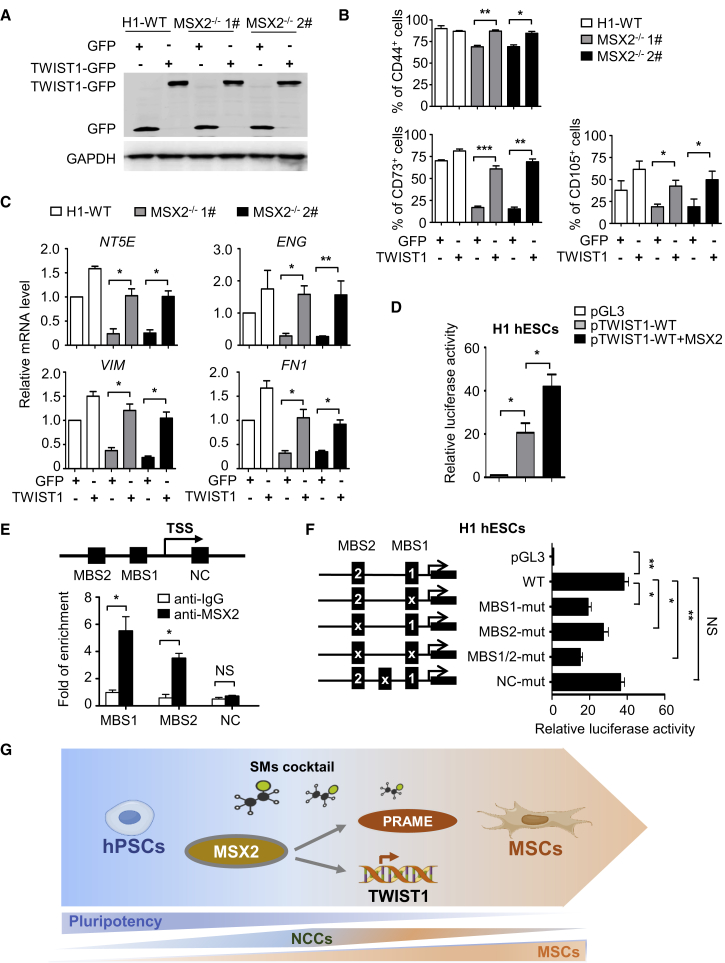
MSX2 Directly Targets TWIST1 during Mesenchymal Differentiation (A) Western blotting analysis of exogenous TWIST1 or GFP in H1-WT and MSX2-deleted H1 cells at day 7 of MSC induction under SMs conditions. GAPDH was used as a loading control. (B and C) FCM analysis (B) and qRT-PCR analysis (C) of indicated MSC markers in H1 and MSX2-deleted H1 cells without or with TWIST1 overexpression at day 7 of MSC induction under SMs conditions (mean ± SEM, N = 3). ^∗^p < 0.05; ^∗∗^p < 0.01; ^∗∗∗^p < 0.001. (D) Relative luciferase activity in GFP-MSX2 H1 hESCs transfected with pGL3 construct containing TWIST1 promoter (pTWIST1-0.7kb-LUC) ± DOX (3 μg/mL) for 72 hr (mean ± SEM, N = 3). ^∗^p < 0.05. Values are normalized to the pGL3 group (=1). (E) ChIP-qPCR analysis of the occupancy of MSX2 on the two potential MSX2-binding sites (MBS1, MBS2) of TWIST1 promoter in GFP-MSX2 H1 hESCs with DOX (3 μg/mL) for 72 hr. Non-specific immunoglobulin G was used as isotype control. Values are normalized to those of their corresponding input samples (mean ± SEM, N = 3). ^∗^p < 0.05; NS, not significant. (F) Relative luciferase activity in GFP-MSX2 H1 hESCs transfected with WT or MSX2-binding site mutated (MBS1-mut, MBS1-mut, MBS1/2-mut) TWIST1 promoter-luciferase reporter constructs with DOX (3 μg/mL) for 3 days. A non-specific mutant in TWIST1 5′ flanking region was used as a negative control (NC-mut). Normalized to the cells transfected with pGL3 (=1) (mean ± SEM, N = 3). ^∗^p < 0.05; ^∗∗^p < 0.01; NS, not significant. Values are normalized to the pGL3 group (=1). (G) Schematic model for efficient hPSC-MSC induction and the underlying mechanism. Based on MSX2 and specific SMs cocktail, hPSCs can be directly programmed into MC-MSCs through an NCC intermediate stage. During the process, MSX2 upregulates the expressions of PRAME and TWIST1. Furthermore, TWIST1 serves as a key direct target of MSX2 and mediates its programming function. See also Figure S7.

Having identified PRAME and TWIST1 as key regulators of hPSC mesenchymal differentiation, we asked whether they could be modulated by MSX2. We first determined whether PRAME or TWIST1 overexpression could rescue the defects caused by MSX2 knockout. We confirmed ectopic PRAME and TWIST1 expression by using western blotting or emergence of GFP fluorescence (Figures 7A, S7A, and S7B). Interestingly, overexpression of TWIST1, but not PRAME, rescued the decrease of CD73^+^ and CD105^+^ cells caused by MSX2 knockout (Figures 7B and S7C). qRT-PCR analysis further showed that the mRNA levels of NT5E, ENG, VIM, and FN1 were restored upon TWIST1 overexpression (Figure 7C). Thus, TWIST1 may serve as a downstream target of MSX2 during MSC differentiation of hPSCs.

To assess whether MSX2 directly targets TWIST1, we isolated the *TWIST1* 5′ flanking sequence of various lengths (0.7 and 1.4 kb) and tested their responses to MSX2 ectopic expression using a luciferase-based reporter assay. Indeed, the two *TWIST1* promoter fragments responded to MSX2 overexpression by increasing the luciferase activity (Figures 7D and S7D). The 0.7 kb fragment was used for further study because no significant difference was seen between the two fragments (Figure S7D). Interestingly, two potential MSX2 binding sites (MBSs), MBS1 (CCAATGAC) and MBS2 (CGAATTGT), were identified within this fragment, while chromatin immunoprecipitation (ChIP) analysis showed that the area containing either MBS1 or MBS2 could be enriched by MSX2 (Figure 7E). Further, mutations of these two sites severely impaired TWIST1 activation by MSX2 both in H1 and BC1 hPSCs (Figures 7F and S7E). Thus, the two MBSs are likely MSX2 binding sites within the TWIST1 promoter that are functional during MSC differentiation of hPSCs.

## Discussion

In this study, we found that MSX2 is sufficient to initiate the mesenchymal differentiation program in hPSCs. By taking advantage of MSX2 as a programming factor and addition of soluble factors, we establish a novel strategy to differentiate hPSCs into MSCs within a week without any co-culture or EB utilization. To our knowledge, this is a more rapid procedure than any has been described thus far for MSC differentiation from hPSCs. The transcriptome analysis further reveals a stepwise early developmental process of human MSCs with neural crest identified as the intermediate stage between pluripotency and mesenchymal fate. We also discovered PRAME and TWIST1 as essential regulators in mediating MSC differentiation from hPSCs (Figure 7G).

We previously demonstrated that MSX2 functions to mediate the entry of hPSCs into mesendoderm during hPSC early fate specification (Wu et al., 2015). In this study, we examined the role of MSX2 in mesenchymal differentiation and found that MSX2 is also essential. In animal models, Msx2 deletion results in profound defects in the development of multiple organs, including skull vault, tooth, hair follicle, and mammary gland (Alappat et al., 2003, Satokata et al., 2000, Wilkie et al., 2000). Mutations of MSX2 are associated with Boston-type craniosynostosis and parietal foramina in human development (Jabs et al., 1993, Wilkie et al., 2000). It has been speculated that the function of MSX2 in the development of the aforementioned organs is linked to its ability to regulate epithelial to mesenchymal transition (Richter et al., 2014, Thiery and Sleeman, 2006). Our studies on hPSC mesenchymal differentiation indicate that the function of MSX2 in mesengenesis is conserved from animals to human.

Interestingly, MSX2 itself is sufficient to initiate the mesenchymal differentiation program in hPSCs. This function is largely unknown in hPSCs, thus revealing MSX2 as a stem cell programming factor. Indeed, with the aid of a number of chemical compounds and growth factors, MSX2 programs hPSCs into functional MSCs within a week, thereby significantly accelerating MSC generation compared with previous described methods involving co-culture or EB induction (Barberi et al., 2005, Mahmood et al., 2010). MSCs generated from this system show *in vivo* function comparable with BM-MSCs (Wang et al., 2016). Since the ectopic MSX2 was delivered via lentivirus, MSCs generated using the current differentiation protocol currently cannot be used for therapeutic purposes.

By using time course genome-wide gene profiling analysis, we also discover a molecular roadmap of MSC generation from hPSCs. Interestingly, we identify neural crest as the intermediate stage occurring between pluripotency and mesenchymal fate. Different models with respect to the intermediate stage during MSC differentiation from hPSCs have been proposed previously, including mesoderm, endoderm, neural crest, or trophoblast lineage (Morikawa et al., 2009, Murray et al., 2014, Slukvin and Vodyanik, 2011, Vodyanik et al., 2010, Wang et al., 2016). With lineage tracing studies *in vivo*, it has been shown that Sox1^+^ neuroepithelium can give rise to MSCs in part through a neural crest intermediate stage (Takashima et al., 2007). Together, these results indicate neural crest can be a physiological stage during human mesengenesis rather than a culture artificial *in vitro*. By utilizing the rapid and high-efficiency MSC differentiation model, we provide convincing evidence supporting the neural crest intermediate stage. Our previous studies revealed that MSX2 is essential for mesendoderm induction from hPSCs. Interestingly, this study demonstrated that MSX2 induces hPSC differentiation to MSCs mainly via a neural crest intermediate. We speculate that the differences of both MSX2 induction time and culture media might lead to the different outcomes in these two studies. Thus, our studies confirm and extend previous findings, demonstrating that functional MSCs can be generated from hPSCs via the neural crest stage.

Very limited studies have been conducted to dissect the mechanism for mesenchymal differentiation from hPSCs (Deng et al., 2016, Yu et al., 2017). With the MSX2-based differentiation strategy and additional profiling analysis, we identified a large number of genes associated with MSCs generation from hPSCs, of which TWIST1 and PRAME are validated functionally as essential regulators. Our findings of TWIST1 in mesenchymal differentiation of hPSCs are consistent with its previously described role in mesenchymal development and epithelial-mesenchymal transition (Kang and Massague, 2004, Mani et al., 2008). At the mechanistic level, we found that MSX2 binds directly to the promoter of TWIST1 and activates it expression. To our knowledge, the molecule link between TWIST1 and MSX2 and the underlying regulation were largely unknown before. We also identify that PRAME, a germinal tissue-specific gene that is also expressed at high hematological malignancies and solid tumors (Epping et al., 2005, Oehler et al., 2009), is essential for mesenchymal differentiation. This is a novel function of PRAME that has never been documented. Despite the lack of modulation of PRAME by MSX2, it will be of enormous interest to further explore how PRAME controls human mesenchymal development and whether PRAME promotes carcinogenesis by giving the cells mesenchymal characteristics during cancer progression. Furthermore, we found that MSX2 is a general effector mediating MSC induction from all the three intermediate sources: neural crest, mesendoderm, and trophoblast. It will be intriguing to investigate whether TWIST1 and PRAME function as downstream targets of MSX2 during MSC induction from mesendoderm and trophoblast.

## Experimental Procedures

### hPSC-MSC Differentiation

To differentiate hPSCs into MSCs, hPSCs (H1 and H9 hESCs or BC1 and Z-15 hiPSCs) were separated into single cells by using Accutase (Gibco) and seeded into 12-well plates coated with growth factor-reduced gel (Stem Cell Technologies) in E8 medium supplemented with Y27632 (10 μM) (Sigma) at a density of 1.5 × 10^4^/mL. After 2 days (day 0), the medium was changed to DMEM/F12 basal media supplemented with 2% fetal bovine serum (Australia), 1% L-glutamine (Gibco) and 1% NEAA (Gibco), 4 ng/mL TGF-β1 (PeproTECH), 4 ng/mL bFGF (PeproTECH), 0.5 μM CHIR99021 (Selleck), and 20 nM DAC (Sigma) from day 0 to day 5, and then the medium was changed to 2% FBS/DMEM-F12 media containing 1% L-glutamine (Gibco), 1% NEAA (Gibco), and 20 nM DAC at day 6–7. The medium was changed every day. As to GFP-MSX2 H1 hESCs or GFP-MSX2 BC1 hiPSCs, 3 μg/mL DOX was added to induce MSX2 expression during the differentiation process. Other factors tested during the differentiation process are listed in Table S3.

### Ethical Approval

hBM-MSCs or mice were used under approval of research ethics (approval no. KT2014005-EC-1 for hBM-MSCs, KT2016011-EC-1 for mice) from the Laboratory Animal Center of Institute of Hematology & Blood Diseases Hospital, Chinese Academy of Medical Sciences & Peking Union Medical College.

### Mouse Model of DSS-Induced Colitis

The mouse model of acute colitis was performed as reported (Wang et al., 2016) and is described in Supplemental Experimental Procedures. All animal studies were approved (approval no. KT2016011-EC-1) by the Laboratory Animal Center of the Institute of Hematology & Blood Diseases Hospital, Chinese Academy of Medical Sciences and Peking Union Medical College (license no. SCXK & SYXK, 2005-0001, Tianjin).

### RNA-Seq and Bioinformatics Analysis

Human bone marrow-derived MSCs, M-MSCs, MC-MSCs, and the cells collected at indicated times of MSC differentiation from DOX-inducible GFP-MSX2 H1 hESCs were used to prepare the RNA-seq samples. RNAs were sequenced by BGI (Shenzhen, China) as we previously described (Wu et al., 2015). Data analysis was performed as described in Supplemental Experimental Procedures. RNA-seq data are available under accession number GSE104784 and SRP055541, or in Tables S4 and S5.

### Statistical Analysis

Data are shown as mean ± SEM, N = 3 independent experiments. Statistical calculations were performed using GraphPad Prism 5 software (version v5.01). p < 0.05 was considered statistically significant (^∗^p < 0.05; ^∗∗^p < 0.01, ^∗∗∗^p < 0.001; NS, not significant).

## Author Contributions

L.S.Z., H.T.W., and J.X.Z. coordinated and designed the project. L.S.Z., H.T.W., C.C.L., Q.Q.W., P.S., D.W., J.J.G., W.Z., Y.F.X., and L.H.S. performed the experiments. J.X.Z., L.S.Z., H.T.W., and D.W. analyzed the data. L.S.Z., H.T.W., and J.X.Z. wrote the manuscript. J.X.Z. designed the experiments and edited the manuscript.

## Supplemental Information

Document S1. Supplemental Experimental Procedures, Figures S1–S7, and Tables S1–S3

Table S4. RNA-Seq Data of Inducible GFP-MSX2 H1 hESCs Cultured in mTeSR with DOX (3 μg/mL) Treatment for Indicated Time Points (0 hr, 24 hr, 48 hr, 72 hr), Related to Figures 1A and S1A

Table S5. RNA-Seq Data of Inducible GFP-MSX2 H1 hESCs Cultured in DMEM/F12 with DOX (3 μg/mL) and/or SMs Treatment for Indicated Time Points (FPKM Value; Days 0, 1, 3, 5, 7), Related to Figures 3, 4, 6, and S2–S4

Document S2. Article plus Supplemental Information
