# Supplementary material for: MSX2 Initiates and Accelerates Mesenchymal Stem/Stromal Cell Specification of hPSCs by Regulating TWIST1 and PRAME
Source: Stem Cell Reports. 2018 Jul 19;11(2):497–513. doi: 10.1016/j.stemcr.2018.06.019 (PMC6092836; doi:10.1016/j.stemcr.2018.06.019)
Supplement: Document S1. Supplemental Experimental Procedures, Figures S1–S7, and Tables S1–S3 [file mmc1.pdf]

Stem Cell Reports, Volume *11*

Supplemental Information

MSX2 Initiates and Accelerates Mesenchymal Stem/Stromal Cell Specification of hPSCs by Regulating TWIST1 and PRAME

Leisheng Zhang, Hongtao Wang, Cuicui Liu, Qingqing Wu, Pei Su, Dan Wu, Jiaojiao Guo, Wen Zhou, Yuanfu Xu, Lihong Shi, and Jiaxi Zhou

Figure. S1

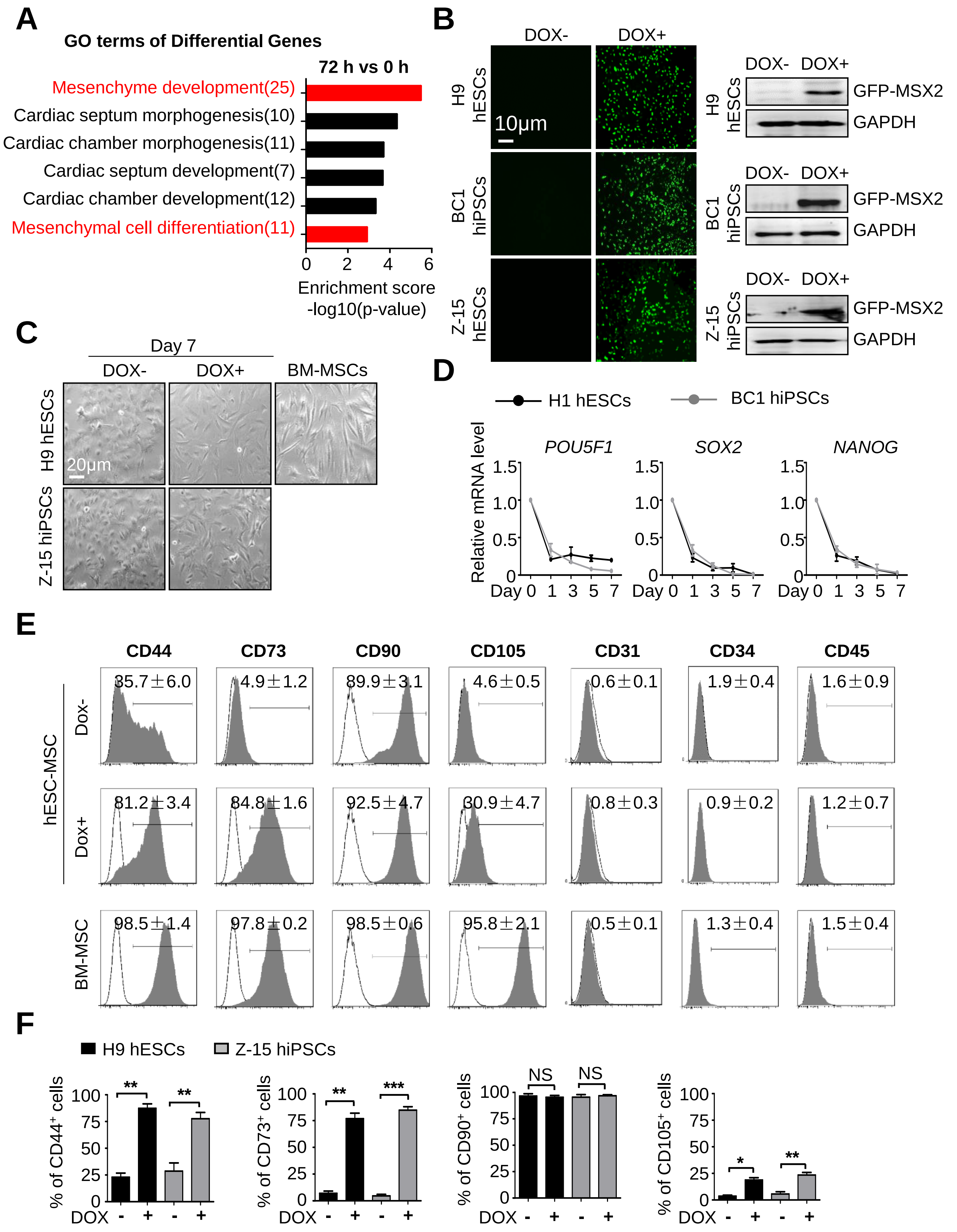
A
GO terms of Differential Genes
72 h vs 0 h
Mesenchyme development(25)
Cardiac septum morphogenesis(10)
Cardiac chamber morphogenesis(11)
Cardiac septum development(7)
Cardiac chamber development(12)
Mesenchymal cell differentiation(11)
0 2 4 6
Enrichment score
-log10(p-value)
B
DOX- DOX+
H9 hESCs
BC1 hiPSCs
Z-15 hESCs
10μm
GFP-MSX2
GAPDH
Z-15 hiPSCs
C
Day 7
DOX- DOX+ BM-MSCs
H9 hESCs
Z-15 hiPSCs
20μm
D
H1 hESCs
BC1 hiPSCs
POU5F1
SOX2
NANOG
Relative mRNA level
1.5 1.0 0.5 0
Day 0 1 3 5 7
E
CD44 CD73 CD90 CD105 CD31 CD34 CD45
hESC-MSC
Dox-
35.7±6.0 4.9±1.2 89.9±3.1 4.6±0.5 0.6±0.1 1.9±0.4 1.6±0.9
Dox+
81.2±3.4 84.8±1.6 92.5±4.7 30.9±4.7 0.8±0.3 0.9±0.2 1.2±0.7
BM-MSC
98.5±1.4 97.8±0.2 98.5±0.6 95.8±2.1 0.5±0.1 1.3±0.4 1.5±0.4
F
H9 hESCs
Z-15 hiPSCs
% of CD44+ cells
% of CD73+ cells
% of CD90+ cells
% of CD105+ cells
100 75 50 25 0
** ** *** NS *
DOX - + - +

Figure S1. Related to Figure 1. MSX2 initiates mesenchymal program in hPSCs. (A) Gene ontology analysis for up-regulated genes in DOX-inducible GFP-MSX2 H1 hESCs with 3μg/ml DOX treatment (72h vs 0h). (B) Left panel: fluorescence images of GFP-MSX2 H9 hESCs, BC1 and Z-15 hiPSCs with or without DOX (3 μg/ml) treatment for 48 h. Right panel: Western blot analysis of MSX2 in GFP-MSX2 H9 hESCs, BC1 and Z-15 hiPSCs with or without DOX (3 μg/ml) treatment for 48 h. Scale bar=10μm. (C) Phase contrast images of GFP-MSX2 H9 hESCs and Z-15 hiPSCs during differentiation in DMEM/F12 media containing 2% FBS with or without DOX (3 μg/ml) treatment. A time-course analysis is shown (from 0 h to 120 h). BM-MSCs was used as a control. Scale bar=20μm. (D) Time-course analysis of the pluripotent genes (POU5F1, SOX2 and NANOG) expression in GFP-MSX2 H1 hESCs and BC1 hiPSCs with DOX (3 μg/ml) treatment for indicated time by qRT-PCR. All values are normalized to the level (= 1) of mRNA in the cells before adding DOX (0 h). Results are shown as mean ± SEM (N=3). (E) FCM analysis for MSC markers (CD44, CD73, CD90, CD105), endothelial markers (CD31, CD34) and hematopoietic marker (CD45) of GFP-MSX2 H1 hESCs cultured in DMEM/F12 media containing 2% FBS with or without DOX (3 μg/ml) treatment for 7 days. All data are shown as mean ± SEM. (F) FCM analysis of MSC markers (CD44, CD73, CD90, CD105) of GFP-MSX2 H9 hESCs and Z-15 hiPSCs cultured in DMEM/F12 media containing 2% FBS with or without DOX (3 μg/ml) treatment for 7 days. All data are shown as mean ± SEM (N=3).

Figure. S2

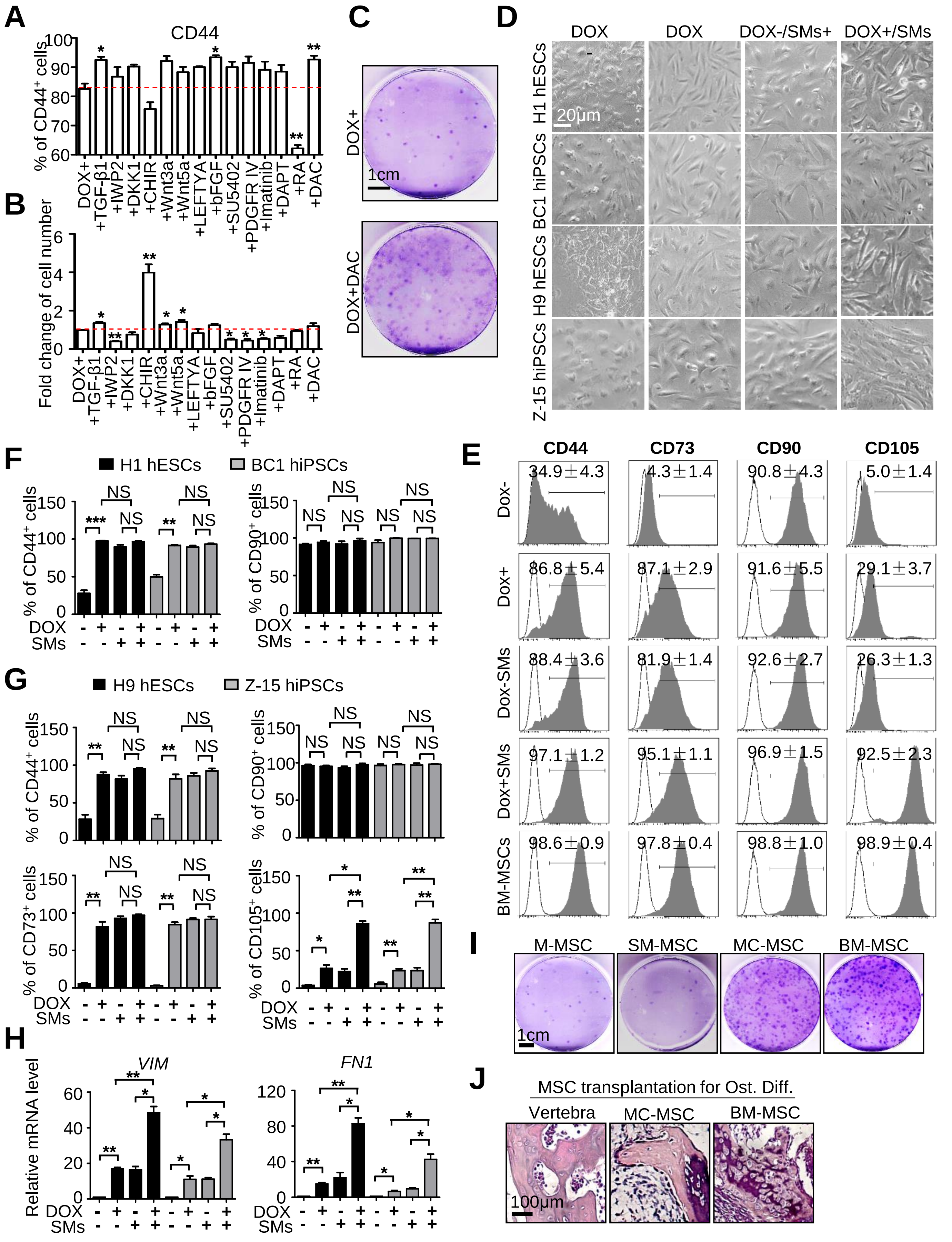
A
CD44
% of CD44+ cells
DOX+
+TGF-β1
+IWP2
+DKK1
+CHIR
+Wnt3a
+Wnt5a
+LEFTYA
+bFGF
+SU5402
+PDGFR IV
+Imatinib
+DAPT
+RA
+DAC
B
Fold change of cell number
C
DOX+
1cm
DOX+DAC
D
DOX
DOX
DOX-/SMs+
DOX+/SMs
H1 hESCs
20μm
BC1 hiPSCs
H9 hESCs
Z-15 hiPSCs
E
CD44
CD73
CD90
CD105
Dox-
34.9±4.3
4.3±1.4
90.8±4.3
5.0±1.4
Dox+
86.8±5.4
87.1±2.9
91.6±5.5
29.1±3.7
Dox-SMs
88.4±3.6
81.9±1.4
92.6±2.7
26.3±1.3
Dox+SMs
97.1±1.2
95.1±1.1
96.9±1.5
92.5±2.3
BM-MSCs
98.6±0.9
97.8±0.4
98.8±1.0
98.9±0.4
F
H1 hESCs
BC1 hiPSCs
% of CD44+ cells
% of CD90+ cells
DOX
SMs
G
H9 hESCs
Z-15 hiPSCs
% of CD44+ cells
% of CD90+ cells
% of CD73+ cells
% of CD105+ cells
DOX
SMs
H
VIM
FN1
Relative mRNA level
DOX
SMs
I
M-MSC
SM-MSC
MC-MSC
BM-MSC
1cm
J
MSC transplantation for Ost. Diff.
Vertebra
MC-MSC
BM-MSC
100μm

Figure S2. Related to Figure 2. Chemical Compounds further facilitate the MSCs generation initiated by MSX2. (A) FCM analysis for MSCs markers (CD44) of GFP-MSX2 H1 hESCs cultured in DMEM/F12 media containing 2% FBS and 3 μg/ml DOX with indicated chemical compound (see Supplemental Table S3) treatment for 7 days. IWP2 (2μM), DKK1 (200nM), CHIR (2μM), Wnt3a (10nM), Wnt5a (10nM), LEFTYA (500nM), SU5402 (2μM), PDGFR IV (5nM), Imatinib (10mg/ml), DAPT (10nM), RA (5nM). All data are shown as mean ± SEM (N=3), *, $P<0.05$; **, $P<0.01$; NS, not significant (not shown). (B) Fold change of total cell number after treatment as shown in Figure S2A. All values were normalized to the level (= 1) of the group without chemical compound treatment. All data are shown as mean ± SEM (N=3), *, $P<0.05$; **, $P<0.01$; NS, not significant. (C) 100 GFP-MSX2 H1 hESC-derived cells with DOX+ or DOX+DAC were seeded in 10 cm dish with 10%FBS/DMEM-F12 media and cultured for two weeks. The colonies were fixed and stained by crystal violet dye before being scanned. Scale bar, 1cm. (D) Phase contrast images of GFP-MSX2 hPSCs (H1 and H9 hESCs, BC1 and Z-15 hiPSCs) after treatment with DOX-, DOX+, DOX-SMs, DOX+SMs for 7 days. Scale bar=20μm. (E) FCM analysis for MSC markers (CD44, CD90, CD73 and CD105) of GFP-MSX2 H1 hESCs cultured in DMEM/F12 media containing 2% FBS with DOX-, DOX+, DOX-SMs, DOX+SMs treatment for 7 days. (F) FCM analysis for MSC markers (CD44, CD90, CD73 and CD105) in GFP-MSX2 H1 hESCs and BC1 hiPSCs cultured in DMEM/F12 media containing 2% FBS with DOX-, DOX+, DOX-SMs, DOX+SMs treatment for 7 days. All data are shown as mean ± SEM (N=3), **, $P<0.01$; ***, $P<0.001$; NS, not significant. (G) FCM analysis for CD44, CD73, CD90, CD105 expression in GFP-MSX2 H9 hESCs and Z-15 hiPSCs after treatment with DOX-, DOX+, DOX-SMs, DOX+SMs for 7 days. Results are shown as mean ± SEM (N=3). *, $P<0.05$; **, $P<0.01$; ***, $P<0.001$; NS, not significant. (H) qRT-PCR analysis of MSC markers (VIM, FN1) in GFP-MSX2 H1 hESCs and BC1 hiPSCs after treatment with indicated treatments for 7 days. All values are normalized to the level (= 1) of mRNA in the group (DOX-). Data are shown as mean±SEM (N=3). *P<0.05; **P<0.01. (I) 100 derived M-MSCs, SM-MSCs, MC-MSCs or BM-MSCs were seeded in 10 cm dish with 10%FBS/DMEM-F12 media and cultured for two weeks. The colonies were fixed and stained by crystal violet dye before being scanned. Scale bar, 1cm. (J) Bone formation in vivo by M-MSCs or MC-MSCs derived from H1 hESCs in 6-week nude mice. Vertebra of 6-week nude mice was used as a positive control. Scale Bar, 100 μm.

Figure. S3

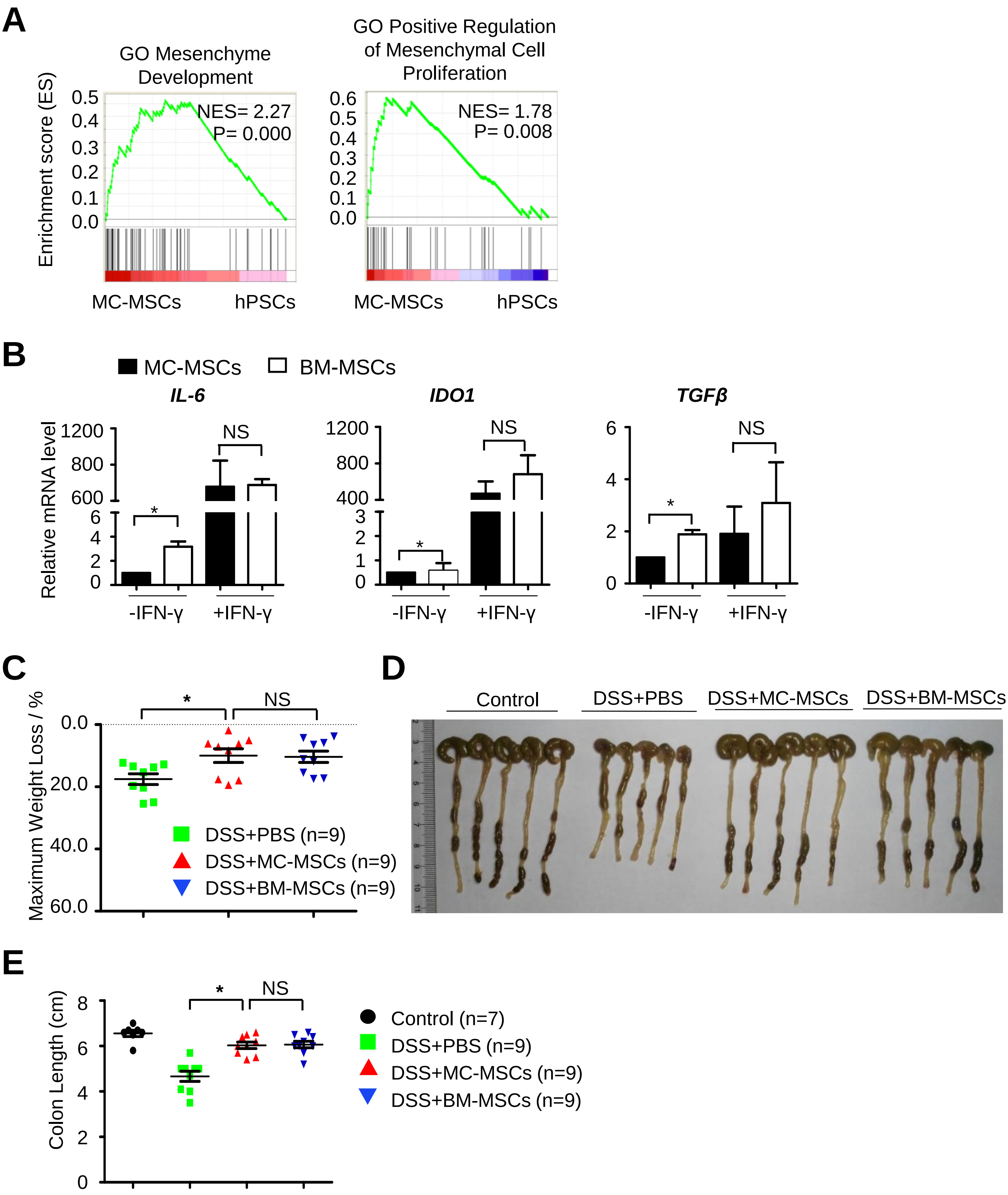

A
GO Mesenchyme Development
Enrichment score (ES)
NES= 2.27
P= 0.000
MC-MSCs
hPSCs
GO Positive Regulation of Mesenchymal Cell Proliferation
NES= 1.78
P= 0.008
MC-MSCs
hPSCs
B
MC-MSCs
BM-MSCs
IL-6
IDO1
TGFβ
Relative mRNA level
NS
*
-IFN-γ
+IFN-γ
C
Maximum Weight Loss / %
*
NS
DSS+PBS (n=9)
DSS+MC-MSCs (n=9)
DSS+BM-MSCs (n=9)
D
Control
DSS+PBS
DSS+MC-MSCs
DSS+BM-MSCs
E
Colon Length (cm)
*
NS
Control (n=7)
DSS+PBS (n=9)
DSS+MC-MSCs (n=9)
DSS+BM-MSCs (n=9)

Figure S3. Relative to Figure 3. MC-MSCs resemble BM-MSCs and show similar therapeutic effect on mice colitis as BM-MSCs. (A) Gene set enrichment analysis (GSEA) comparing MC-MSCs and hPSCs. The normalized enrichment scores (NES) and *P* values are showed in each plot. (B) MC-MSCs or BM-MSCs were cultured in 10% FBS/DMEM-F12 media with or without10ng/ml human IFN-γ for 24 h. The expression of IL-6, IDO1, TGFβ were measured by qRT-PCR analysis. All data are shown as means±SEM (N=3). (C) The maximum proportion of body weight loss for mice in the DSS treated groups (DSS+PBS, DSS+MC-MSCs, DSS+BM-MSCs). Results are shown as mean±SEM. All data were analyzed by students' T test, *, $P<0.05$; NS, not significant. (D) Images of colons dissected from mice in the four groups (DSS+PBS, DSS+MC-MSCs, DSS+BM-MSCs) at day 13 of the experiment. (E) The length of colons dissected from mice in the four groups at day 13 of the experiment. Data are analyzed using paired T test and shown as mean±SEM, *P<0.001; NS, not significant.

Figure. S4

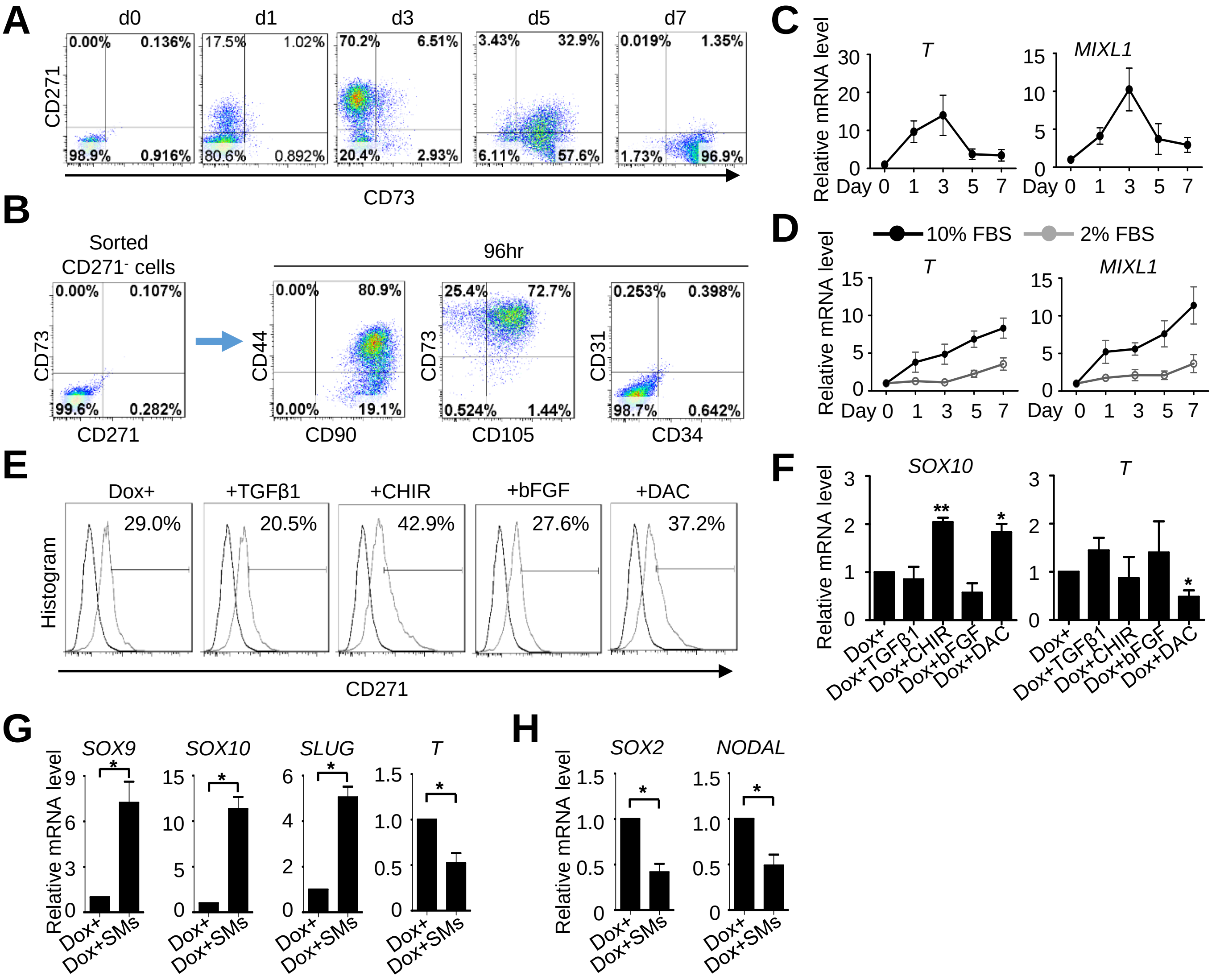

A
d0
d1
d3
d5
d7
CD271
CD73
B
Sorted CD271- cells
96hr
CD73
CD271
CD44
CD90
CD105
CD31
CD34
C
Relative mRNA level
T
MIXL1
Day 0 1 3 5 7
D
10% FBS
2% FBS
E
Dox+
+TGFβ1
+CHIR
+bFGF
+DAC
29.0%
20.5%
42.9%
27.6%
37.2%
Histogram
CD271
F
SOX10
Dox+
Dox+TGFβ1
Dox+CHIR
Dox+bFGF
Dox+DAC
G
SOX9
SLUG
Dox+SMs
H
SOX2
NODAL

Figure S4. Relative to Figure 4. Transcriptome analysis of DEGs during MSCs generation of hPSCs. (A) FCM analysis for the percentage of $CD271^{+}$ and $CD73^{-}$ cells(NCCs) in GFP-MSX2 H1 hESCs with DOX+SMs treatment at indicated time points of MSC induction. (B) MSC potential analysis of $CD271^{-}$ cells derived from inducible GFP-MSX2 H1 hESCs. $CD271^{-}$ cells were isolated at day3 of differentiation and cultured under DOX+SMs conditions for 96 hours, followed by FCM analysis. (C) qRT-PCR analysis of the dynamic expression for mesoendoderm associated genes (T, MIXL1) during MSC induction from DOX-inducible GFP-MSX2 overexpressing H1 hESCs under DOX+SMs conditions. All data are shown as mean ± SEM (N=3). (D) qRT-PCR analysis of the dynamic mRNA expression for mesoendoderm (T, MIXL1) associated genes in H1 hESCs under 2%, 5% FBS condition at indicated time points. All data are shown as mean ± SEM (N=3). (E) FCM analysis for the percentage of $CD271^{+}$ NCCs in GFP-MSX2 H1 hESCs with DOX+, DOX+TGFβ1, DOX+bFGF, DOX+CHIR, DOX+DAC treatment at day 3 of MSC induction. (F) qRT-PCR analysis of the expression of NCCs (SOX10) and mesoendoderm (T) associated genes in GFP-MSX2 H1 hESCs with DOX+, DOX+TGFβ1, DOX+bFGF, DOX+CHIR, DOX+DAC treatment at day 3 of MSC induction. (G) qRT-PCR analysis of the expression of NCCs (SOX9, SOX10, SLUG) and mesoendoderm (T) associated genes in GFP-MSX2 H1 hESCs with DOX+, DOX+SMs treatment at day 3 of MSC induction. (H) qRT-PCR analysis of the expression of SOX2 and NODAL in GFP-MSX2 H1 hESCs with DOX+, DOX+SMs treatment at day 3 of MSC induction.

Fig. S5

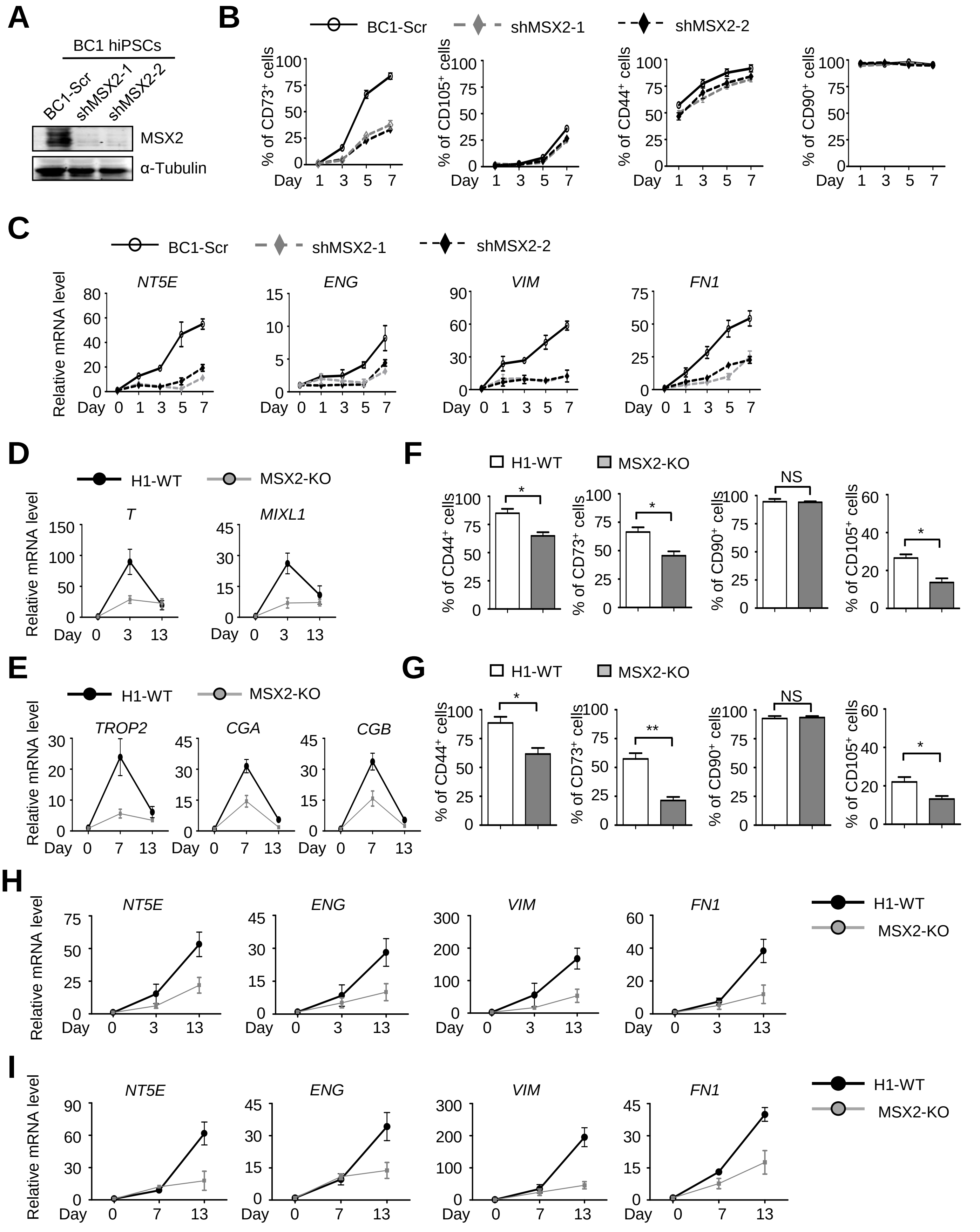

A
BC1 hiPSCs
BC1-Scr
shMSX2-1
shMSX2-2
MSX2
α-Tubulin
B
BC1-Scr
shMSX2-1
shMSX2-2
% of CD73+ cells
% of CD105+ cells
% of CD44+ cells
% of CD90+ cells
Day 1 3 5 7
C
BC1-Scr
shMSX2-1
shMSX2-2
NT5E
ENG
VIM
FN1
Relative mRNA level
Day 0 1 3 5 7
D
H1-WT
MSX2-KO
T
MIXL1
Relative mRNA level
Day 0 3 13
E
H1-WT
MSX2-KO
TROP2
CGA
CGB
Relative mRNA level
Day 0 7 13
F
H1-WT
MSX2-KO
% of CD44+ cells
% of CD73+ cells
% of CD90+ cells
% of CD105+ cells
NS
G
H1-WT
MSX2-KO
% of CD44+ cells
% of CD73+ cells
% of CD90+ cells
% of CD105+ cells
NS
H
NT5E
ENG
VIM
FN1
H1-WT
MSX2-KO
Relative mRNA level
Day 0 3 13
I
NT5E
ENG
VIM
FN1
H1-WT
MSX2-KO
Relative mRNA level
Day 0 7 13

Figure S5. Relative to Figure 5. MSX2 depletion impaired the neural crest, mesoderm or trophoblast derived MSCs. (A) Western-blotting analysis conformed the expression of MSX2 in BC1 hiPSCs depleted of MSX2 or expressing a scramble shRNA (Scr) after MSC induction for 7 days under SM conditions. α-Tubulin was used as a loading control. (B) FCM analysis for MSC markers (CD44, CD73, CD90, CD105) for indicated time in BC1 hiPSCs depleted of MSX2 or expressing a Scr shRNA after MSC induction under SMs conditions (mean±SEM, N=3). (C) Time-course analysis of MSC-specific markers (NT5E, ENG, VIM, FN1) in BC1 hiPSCs depleted of MSX2 or expressing a Scr shRNA after MSC induction under SMs conditions. All values are normalized to the level (= 1) of mRNA in the cells before adding DOX (0 h) (mean±SEM, N=3). (D) qRT-PCR analysis of the dynamic mRNA expression for mesoendoderm associated genes (T, MIXL1) during MSC induction from WT H1 and MSX2-deleted H1 hESCs under EB conditions. All data are shown as mean ± SEM (N=3). (E) qRT-PCR analysis of the dynamic mRNA expression for tropoblast associated genes (TROP2, CGA, CGB) during MSC induction from WT H1 and MSX2-deleted H1 hESCs under TB-MSC deriving conditions. All data are shown as mean ± SEM (N=3). (F) FCM analysis for MSC markers (CD44, CD73, CD90, CD105) in WT H1 and MSX2-deleted H1 hESCs after MSC induction under mesoendoderm-MSC deriving conditions. All data are shown as mean ± SEM (N=3). *P<0.05; NS, not significant. (G) FCM analysis for MSC markers (CD44, CD73, CD90, CD105) in WT H1 and MSX2-deleted H1 hESCs after MSC induction under TB-MSC deriving conditions. All data are shown as mean ± SEM (N=3). *P<0.05; *P<0.01; NS, not significant. (H) qRT-PCR analysis of the dynamic mRNA expression for MSC markers (NT5E, ENG, VIM, FN1) during MSC induction from WT H1 and MSX2-deleted H1 hESCs under EB conditions. All data are shown as mean ± SEM (N=3). (I) qRT-PCR analysis of the dynamic mRNA expression for MSC markers (NT5E, ENG, VIM, FN1) during MSC induction from WT H1 and MSX2-deleted H1 hESCs under TB-MSC deriving conditions. All data are shown as mean ± SEM (N=3).

Figure. S6

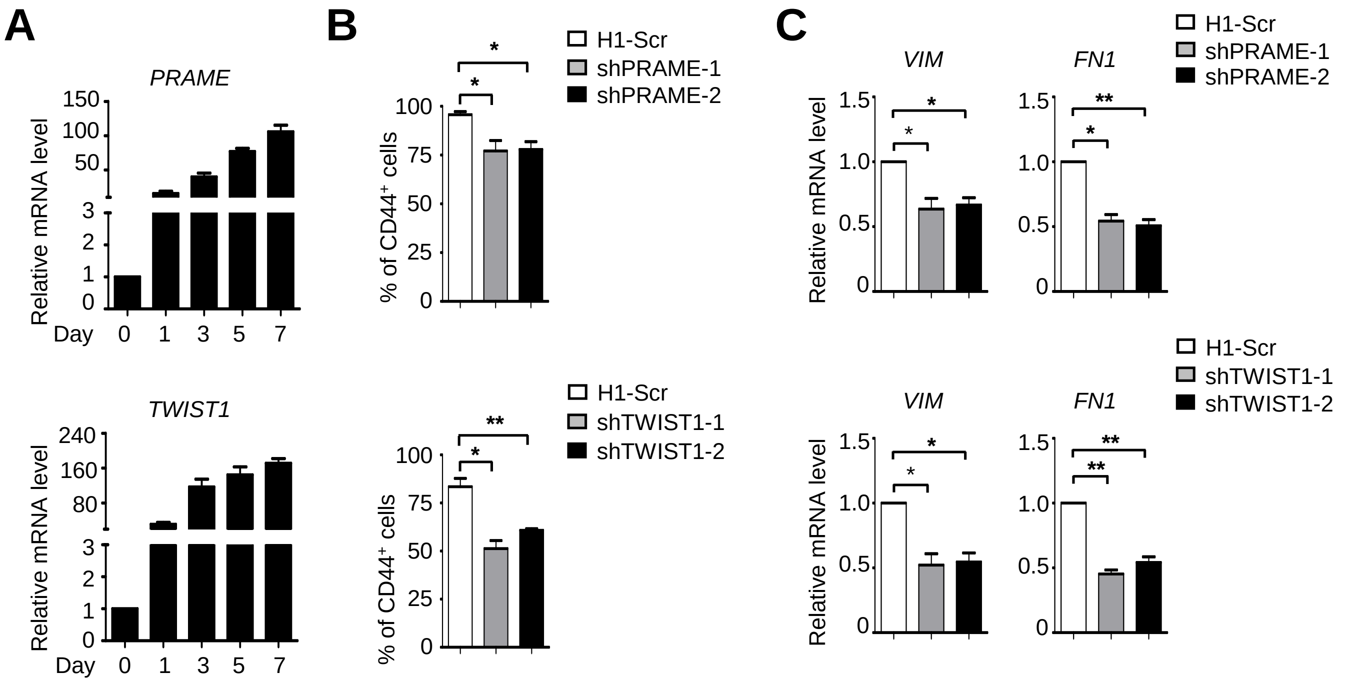
A
PRAME
Relative mRNA level
150
100
50
3
2
1
0
Day 0 1 3 5 7
TWIST1
Relative mRNA level
240
160
80
3
2
1
0
Day 0 1 3 5 7
B
% of CD44+ cells
100
75
50
25
0
*
*
H1-Scr
shPRAME-1
shPRAME-2
*
**
H1-Scr
shTWIST1-1
shTWIST1-2
C
VIM
FN1
Relative mRNA level
1.5
1.0
0.5
0
*
*
**
*
H1-Scr
shPRAME-1
shPRAME-2
VIM
FN1
*
*
**
**
H1-Scr
shTWIST1-1
shTWIST1-2

Figure S6. Relative to Figure 6. The depletion of PRAME or TWIST1 impaired MSC generation. (A) qRT-PCR analysis of the dynamic mRNA expression for PRAME or TWIST1 during MSC induction from H1 hESCs under SM conditions. All data are shown as mean ± SEM (N=3). (B) FCM analysis for MSC markers (CD44) in H1 hESCs depleted of PRAME or TWIST1 or expressing a Scr shRNA after MSC induction for 7 days under SMs conditions (mean±SEM, N=3). *$P<0.05$; **$P<0.01$. (C) qRT-PCR analysis of MSC-specific markers (VIM, FN1) in H1 hESCs depleted of PRAME or TWIST1 or expressing a Scr shRNA after MSC induction for 7 days under SM conditions (mean±SEM, N=3). *$P<0.05$; **$P<0.01$.

Fig. S7

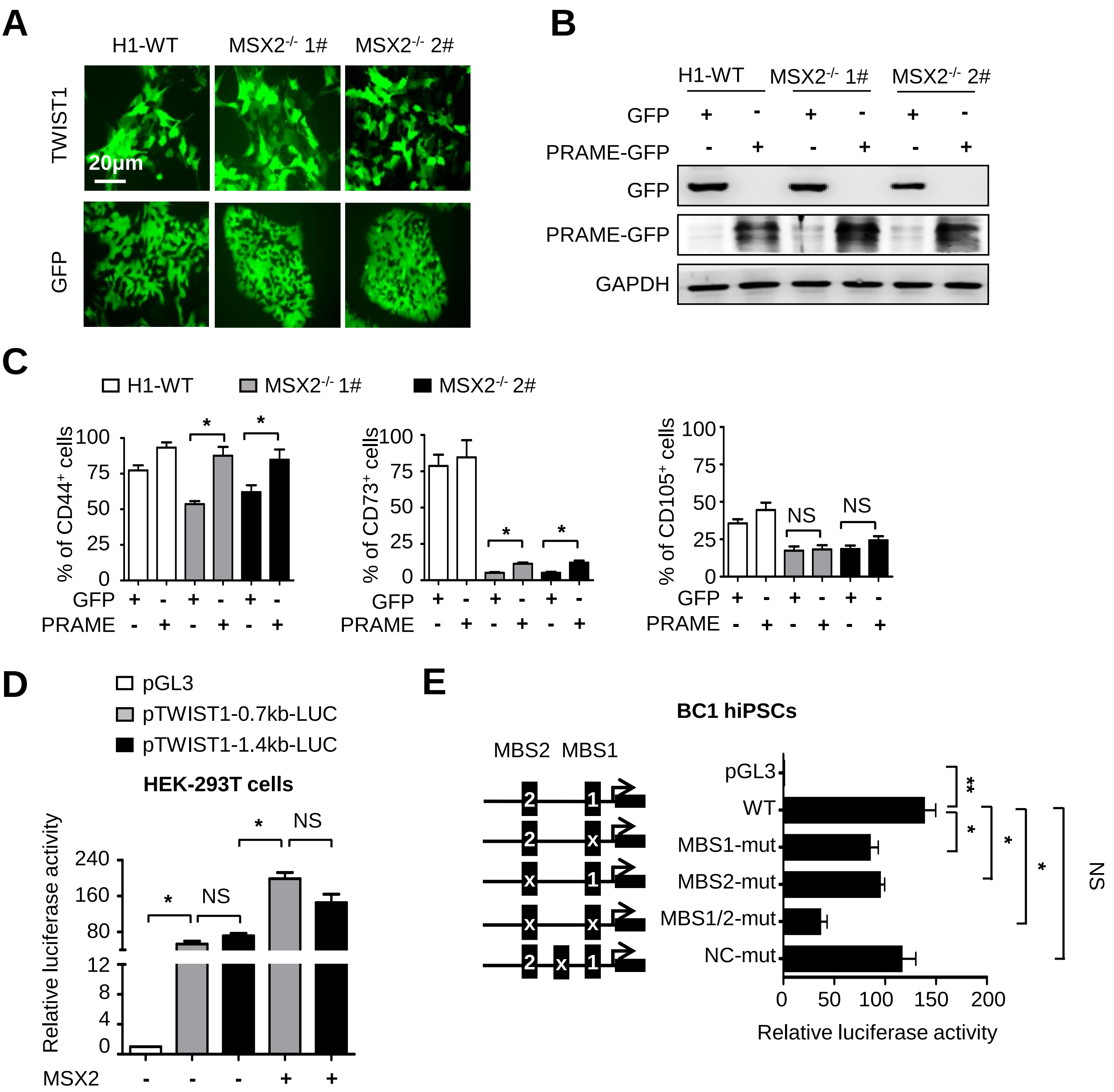
A
H1-WT
MSX2-/- 1#
MSX2-/- 2#
TWIST1
GFP
20μm
B
H1-WT
MSX2-/- 1#
MSX2-/- 2#
GFP
PRAME-GFP
GAPDH
C
H1-WT
MSX2-/- 1#
MSX2-/- 2#
% of CD44+ cells
% of CD73+ cells
% of CD105+ cells
NS
GFP
PRAME
D
pGL3
pTWIST1-0.7kb-LUC
pTWIST1-1.4kb-LUC
HEK-293T cells
Relative luciferase activity
MSX2
E
BC1 hiPSCs
MBS2
MBS1
pGL3
WT
MBS1-mut
MBS2-mut
MBS1/2-mut
NC-mut
Relative luciferase activity

Figure S7. Relative to Figure 7. MSX2 directly targets TWIST1 during mesenchymal differentiation. (A) Fluorescence images of WT H1 and MSX2-deleted H1 cells ($MSX2^{-/-}$ 1#; $MSX2^{-/-}$ 2#) infected with a GFP or TWIST1-GFP vector at day 7 of MSC induction under SMs conditions. Scale bar=20μm. (B) Western blotting analysis of exogenous PRAME or GFP in WT H1 and MSX2-deleted H1 cells ($MSX2^{-/-}$ 1#; $MSX2^{-/-}$ 2#) at day 7 of MSC induction under SMs conditions. GAPDH was used as a loading control. (C) FCM analysis of MSC markers (CD44, CD73, CD105) in H1 and MSX2-deleted H1 cells ($MSX2^{-/-}$ 1#; $MSX2^{-/-}$ 2#) without or with PRAME overexpression at day 7 of MSC induction under SMs conditions. All data are shown as mean ± SEM (N=3). *, $P<0.05$; NS, not significant. (D) Relative luciferase activity in HEK-293T cells co-transfected with psin-EF1α-MSX2 vector and pGL3 basic luciferase construct or pGL3 construct containing TWIST1 promoter (pTWIST1-0.7kb-LUC, pTWIST1-1.4kb-LUC). Renilla plasmid was co-transfected as an internal control. All values are normalized to the level (= 1) of the luciferase activity in cells transfected with pGL3 empty vector. All data are shown as means ± SEM (N=3), *, $P<0.05$; NS, not significant. (E) Relative luciferase activity in GFP-MSX2 BC1 hiPSCs transfected with WT or MSX2-binding site mutated (MBS1-mut, MBS1-mut, MBS1/2-mut) TWIST1 promoter-luciferase reporter constructs with DOX (3 μg/ml) treatment for 3 days. A non-specific mutant in TWIST1 5′ flanking region was used as a negative control (NC-mut). All values were normalized to the level (= 1) of the luciferase activity in cells transfected with pGL3 basic vector. Results are shown as mean ± SEM (N=3). *, P<0.05; **, P<0.001; NS, not significant.

Supplemental Tables

Table S1. Antibodies Used in This Study. Related to Fig. 1-7 and Fig. S1-S7.

Antibodies for FCM. Related to Fig. 1-7 and Fig. S1, S2, S4, S6.

Antibody	Cat. NO.	Source
Anti-human CD44-PE	550989	BD Pharmigen
Anti- human CD90-APC	328113	BioLegend
Anti- human CD73-PercP CY5.5	46-0739-42	eBioscience
Anti- human CD73-PE	550257	BD Pharmigen
Anti- human CD105-APC	323208	BioLegend
Anti- human CD31-PE	555446	BD Pharmigen
Anti- human CD34-APC	555824	BD Pharmigen
Anti- human CD45-PE	560975	BD Pharmigen
Anti- human CD271-PE	557196	BD Pharmigen
Anti- human CD271-APC	345107	BioLegend
Anti-mouse CD3e-PE	100307	BioLegend
Anti- mouse CD4	100516	BioLegend
Anti- mouse CD8a	100721	BioLegend
Anti- mouse CD8-APC	47-0081-82	eBioscience
Anti-mouse-CD3ε	100314	BioLegend
Anti-mouse-CD28	16-0281-85	eBioscience
Molecular proles Sulfate latex	A37302	Invitrogen

Antibodies for Western-blotting assay. Related to Fig. 5-7 and Fig. S1, S5, S7.

Name	Company	Catalog	Host	Dilution
MSX2	SANTA CRUZ	Sc-15396	Rabbit	1:300
TWIST1	GeneTex	GTX50821	Rabbit	1:500
PRAME	SANTA CRUZ	sc-137188	Mouse	1:1000
GFP	ProteinTech	66002-1-Ig	Mouse	1:2000
α-Tubulin	Abcam	Ab11304	Mouse	1:10000
GAPDH	Proteintech	10494-1-AP	Rabbit	1:2000

Table S2. Primers Used in This Study. Related to Fig. 1-2, 4-7 and Fig S1- S7.

qRT-PCR primer sequences. Related to Fig. 1-2, 4-7 and Fig. S1-S7.

Gene	Forward Primer	Reverse Primer
ACTIN	CTCTTCCAGCCTTCCTTCCT	AGCACTGTGTGTTGGCGTACAG
POU5F1	CTTGAATCCCGAATGGAAAGGG	GTGTATATCCCAGGGTGATCCTC
SOX2	GCCGAGTGGAAACTTTTGTCG	GGCAGCGTGTACTTATCCTTCT
NANOG	TTTGTGGGCCTGAAGAAAACT	AGGGCTGTCCTGAATAAGCAG
MSX2	TTACCACATCCCAGCTCCTC	CCTGGGTCTCTGTGAGGTTC
TWIST1	GTCCGCAGTCTTACGAGGAG	GCTTGAGGGTCTGAATCTTGCT
PRAME	AGCCTTTGACGGGAGACAC	GAGTTCTTCCGTAAATCCAGCA
RUNX2	CTCACTACCACACCTACCTG	TCAATATGGTCGCCAAACAGATTC
BGLAP	GGCGCTACCTGTATCAATGG	TCAGCCAACTCGTCACAGTC
SOX9	AATGGAGCAGCGAAATCAAC	CAGAGAGATTTAGCACACTGATC
COL2A1	TGGACGCCATGAAGGTTTTCT	TGGGAGCCAGATTGTCATCTC
PPAR-γ	GCTGGCCTCCTTGATGAATA	TGTCTTCAATGGGCTTCACA
FABP4	TATGAAAGAAGTAGGAGTGGGC	CCACCACCAGTTTATCATCCTC
ADIPOQ	TGGTCCTAAGGGAGACATCG	TGGAATTTACCAGTGGAGCC
ACAN	CCCCTGCTATTTCATCGACCC	GACACACGGCTCCACTTGAT
NT5E	GGGCGGAAGGTTCCTGTAG	GAGGAGCCATCCAGATAGACA
ENG	AGCCCCACAAGTCTTGCAG	GCTAGTGGTATATGTCACCTCGC
VIM	GAAGAGAACTTTGCCGTTGAAG	GAAGGTGACGAGCCATTT
FN1	TGACCCCTACACAGTTTCCCA	TGATTCAGACATTCGTTCCCAC
GAPDH	GAGGTGTGAGTGGGATGGTGG	GCCTGCTTCACCACCTTCTTG
SOX9	AGCGAACGCACATCAAGAC	CTGTAGGCGATCTGTTGGGG
SOX10	CCTCACAGATCGCCTACACC	CATATAGGAGAAGGCCGAGTAGA
CD271	CCTACGGCTACTACCAGGATG	CACACGGTGTTCTGCTTGT
FOXD3	GCAACTACTGGACCCTGGAC	CTGTAAGCGCCGAAGCTCT
IL-6	ACTCACCTCTTCAGAACGAATTG	CCATCTTTGGAAGGTTCAGGTTG
IDO1	GCCAGCTTCGAGAAAGAGTTG	ATCCCAGAACTAGACGTGCAA
TGFβ	GGCCAGATCCTGTCCAAGC	GTGGGTTTCCACCATTAGCAC
TROP2	GAGATTCCCCCGAAGTTCTC	AACTCCCCCAGTTCCTTGAT
CGA	TGCCCAGAATGCACGCTAC	TTGGACCTTAGTGGAGTGGGA
CGB	TACTGCCCCACCATGACCC	GGTAGTTGCACACCACCTGA
SNAI1	TCGGAAGCCTAACTACAGCGA	AGATGAGCATTGGCAGCGAG
SNAI2	CGAACTGGACACACATACAGTG	CTGAGGATCTCTGGTTGTGGT
T	CTGGGTACTCCCAATGGGG	GGTTGGAGAATTGTTCCGATGA
NODAL	CTGCTTAGAGCGGTTTCAGATG	CGAGAGGTTGGAGTAGAGCATAA
MIXL1	TTTGGCTAGGCCGGAGATTAT	GGGCTTCAGACATTTCGTTTCAG

shRNA sequences and their targets. Related to Figure 4-6 and Fig. S5-S6.

Target	shRNA	Sequence
MSX2	shRNA-1-F	CCGGCCGTTCCATAGACCTGTGCTTCTCGAGAAGCAC AGGTCTATGGAACGGTTTTT
MSX2	shRNA-1-R	AATTCAAAAACCGTTCCATAGACCTGTGCTTCTCGAG AAGCACAGGTCTATGGAACGG
MSX2	shRNA-2-F	CCGGTGCAGGCAGCGTCCATATATGCTCGAGCATATAT GGACGCTGCCTGCATTTTTG
MSX2	shRNA-2-R	AATTCAAAAATGCAGGCAGCGTCCATATATGCTCGAG CATATATGGACGCTGCCTGCA
TWIST1	shRNA-1-F	CCGGGCATTCTGATAGAAGTCTGAACTCGAGTTCAG ACTTCTATCAGAATGCTTTTT
TWIST1	shRNA-1-R	AATTCAAAAAGCATTCTGATAGAAGTCTGAACTCGA GTTCAGACTTCTATCAGAATGC
TWIST1	shRNA-2-F	CCGGCCTGAGCAACAGCGAGGAAGACTCGAGTCTT CCTCGCTGTTGCTCAGGTTTTT
TWIST1	shRNA-2-R	AATTCAAAAACCTGAGCAACAGCGAGGAAGACTC GAGTCTTCCTCGCTGTTGCTCAGG
PRAME	shRNA-1-F	CCGGCCTGTGATGAATTGTTCTCCTCTCGAGAGGAG AACAATTCATCACAGGTTTTTG
PRAME	shRNA-1-R	AATTCAAAAACCTGTGATGAATTGTTCTCCTCTCGAG AGGAGAACAATTCATCACAGG
PRAME	shRNA-2-F	CCGGATGTTGACTTGAGGAGTTAATCTCGAGATTAAC TCCTCAAGTCAACATTTTTTG
PRAME	shRNA-2-R	AATTCAAAAAATGTTGACTTGAGGAGTTAATCTCGA GATTAACTCCTCAAGTCAACA
SOX10	shRNA-1-F	CCGGCCTCATTCTTTGTCTGAGAAACTCGAGTTTCTC AGACAAAGAATGAGGTTTTTG
SOX10	shRNA-1-R	AATTCAAAAACCTCATTCTTTGTCTGAGAAACTCGAG TTTCTCAGACAAAGAATGAGG
SOX10	shRNA-2-F	CCGGGCTGCTGAACGAAAGTGACAACTCGAGTTGTC ACTTTCGTTCAGCAGCTTTTTG
SOX10	shRNA-2-R	AATTCAAAAAGCTGCTGAACGAAAGTGACAACTCGAG TTGTCACTTTCGTTCAGCAGC

Vector clone primer sequences. Related to Figure 7 and Figure S7.

	Primer Sequence
pTWIST1-1.4kb-LUC-F	CCGCTCGAGCAGAAATGCTTAAAATGTTA
pTWIST1-0.7kb-LUC-F	CCGCTCGAGGTCAGACTGGGTCGTTGTAG
pTWIST1-LUC-R	CCCAAGCTTGAGGAGAGAGCAGGAGGACG
pTWIST1-MBS1-mut-LUC-F	CTCACGTCAGGTACCGACAACTGCTGCCCC
pTWIST1-MBS1-mut-LUC-R	GGGGCAGCAGTTGTCGGTACCTGACGTGAG
pTWIST1-MBS2-mut-LUC-F	GGTTTGGGAGGAACCCGGACTAGACCCCGA
pTWIST1-MBS2-mut-LUC-R	TCGGGGTCTAGTCCGGGTTCCTCCCAAACC
pTWIST1-NC-mut-LUC-F	ACGGGGGAGGGCCTGCCCTAAGCGGAAACT
pTWIST1-NC-mut-LUC-R	AGTTTCCGCTTAGGGCAGGCCCTCCCCCGT
GFP-PRAME-F	GGACTAGTGCCACCATGGACTACA<br>AGGACGACGATGACAAGGAACGAAGGCGTTTGTG
GFP-PRAME-R	CTAATTAGGCATGAAACAGG

ChIP-qPCR primer sequences. Related to Figure 7.

MBS	Forward Primer	Reverse Primer
MBS1	GGCCGCCCGGGCCAGGTCGT	CTCCGTGCAGGCGGAAAGTT
MBS2	GGCCGCCCGGGCCAGGTCGT	GGAGGAGGGACTTTTCGAAG
MBS-NC	GGCACCGTTGCCTCGCGCCC	AACGGTCCTTACCCGTGACC

Table S3. Chemical compounds. Related to Figure 1-2, 4-7 and Figure S1-S7

Reagent	Cat. NO.	Source
TGF-β1	100-21	PEPROTECH
TGF-β3	100-36E	PEPROTECH
bFGF	100-18b	PEPROTECH
CHIR99021	S2924	Selleck
SU5402	572630-500UGCN	Millipore
DKK1	120-30	PEPROTECH
IWP2	I0536	Sigma-Aldrich
Wnt3a	5036-WN-010	R&D SYSTEMS
Wnt5a	645-WN-010/CF	R&D SYSTEMS
PDGFR Inhibitor IV	521233	EMD Millipore
Activin A	120-14	PEPROTECH
LEFTYA	746-LF-025/CF	R&D SYSTEMS
SB431542	S1067	SELLECK
BMP-4	120-05	PEPROTECH
Y-27632	S1049	SELLECK
DAPT	D5942	Sigma-Aldrich
IFNγ	300-02	PEPROTECH
Imatinib	CDS022173	Sigma-Aldrich
RA	R2625	Sigma-Aldrich
DAC	A3656	Sigma-Aldrich
BIO	B1686	Sigma-Aldrich

Supplemental Experimental Procedures

Cell culture

hESC lines (H1 and H9) and hiPSC (BC1 and Z-15) were maintained in E8 Essential medium (Gibco) on Matrigel (Stem Cell Technologies) and passaged at a dilution of 1:6 every 4-6 days. The HEK-293T cells were cultured in DMEM (Invitrogen) containing 10% fetal bovine serum (FBS) (Hyclone) and were passaged every 2-3 days. hBM-MSCs were a kind gift from professor Zhongchao Han, which were derived from fresh bone marrow aspirates donated by healthy adults (range 20–56 years) under approval of research ethics (approval no. KT2014005-EC-1) as described previously(Lu et al., 2017). BM-MSCs were cultured in DMEM-F12 (Gibco) media containing 10% FBS, 1% L-Glutamine (Gibco) and 1% NEAA (Gibco). BM-MSCs were passaged every 3-5 days. BM-MSCs at passages 3-8 were used throughout the study.

Western Blotting Analysis

Western blotting analysis was performed as describe previously(Wu et al., 2015). For the detection of target genes expression in protein level, 5×10^6 were lysed with 150μl laemmli sample buffer (BioRad) and inactivated in 100℃ for 5min as we previously did. The samples were electrophoresed on a 10% SDS-PAGE gel and then transferred onto a PVDF membrane (GE Healthcare Life Sciences). The membranes were blocked using 5% nonfat milk (BD) diluted in TBST (PBS containing 0.1% Tween 20) at room temperature for 1 h before being incubated with primary antibody at 4 °C overnight. After being washed with TBST for three times (5 min each), the membranes were incubated with HRP-conjugated secondary antibody (GE Healthcare) at room temperature for 2 h. After three washing using TBST, proteins were detected using the ECL Detection Reagent (Thermo). Finally, the blots were developed by using the Super-Signal West Pico Chemiluminescent Substrate (Pierce). Antibodies used are listed in Supplemental Table S1.

Flow Cytometric Analysis

Differentiated cells were dissociated into single cells using 0.05% Trypsin/EDTA and the cells were stained with the indicated antibodies (listed in Supplemental Table S1) in 0.2% BSA. After incubation for 30 min at room temperature in dark, the cells were washed twice with cold PBS and analyzed using FACS Canto II (BD Biosciences). 7-aminoactinomycin D (7AAD) staining was used for dead cells

exclusion. Data were analyzed with FlowJo software (Tree Star, Ashland, OR). Antibodies used are listed in Supplemental Table S1.

qRT-PCR

Total RNA was harvest with TRIzol reagent (Thermo) according to the manufacturer's instruction and quantified by using NanoDrop 2000 (Thermo). cDNA was synthesized by reverse transcriptase kit (Promaga). qRT-PCR assay was performed with SYBR Green (Qiagen) using the ABI PRISM 7900 (Applied Biosystems). The primer sequences are listed in Supplemental Table S2.

Standard Colony Forming Assay (CFU-F) of MSCs

Standard Colony Forming Assay (CFU-F) of MSCs was performed as previously reported(Meng et al., 2013). Briefly, hBM-MSCs or MSCs derived from hPSCs were detached with 0.05% trypsin and seeded at a density of 100 cells per 10cm dish and cultured in DMEM-F12 (Gbico) supplemented with 10% FBS, 1% L-Glutamine (Gibco) and 1% NEAA (Gibco), 0.5μM CHIR99021 (Selleck). 2 weeks later, the colonies were washed with 1×PBS, fixed with 4% PFA (Gbico) before staining with 0.2% Crystal Violet Solution (Solarbio). The colonies in 10 cm dish was scanned (Canon).

Tri-lineage Differentiation of MSCs

MSCs derived from hPSCs and BM-MSCs were used for adipogenic, osteogenic, and chondrogenic differentiation potential analyses were performed as described previously (Vodyanik et al., 2010; Zhang et al., 2017). All qRT-PCR primer sequences are available in Supplemental Table S2.

For adipogenic differentiation, MSCs derived from hPSCs or BM-MSCs were cultured in adipogenic differentiation medium, which contains IMDM (Gibco), 10% FBS (Hyclone), 1mM Dexamethasone (Sigma), 500μM IBMX (Sigma), 10μg/ml Insulin, 60μM Indomethacin and 2mM L-Glutamine (Gibco). After 3 weeks of culture with medium changed every 3 days, the cells were analyzed using Oil Red O staining and qRT-PCR.

For osteogenic differentiation, MSCs derived from hPSCs or BM-MSCs were maintained in osteogenic differentiation medium containing IMDM (Gibco), 10% FBS (Hyclone), 100nM Dexamethasone (Sigma), 50μg/ml Ascorbic acid (Sigma), 10μM β-Glycerophosphate (Sigma), 2mM L-Glutamine (Gibco) and 1× Nonessential amino acids (Gibco). The medium was changed every 3 days. After 3

weeks of culture, the cells were subjected to Von Kossa staining and harvested for qRT-PCR analysis. For chondrogenic differentiation, MSCs derived from hPSCs or BM-MSCs were cultured in chondrogenic differentiation medium consisting of IMDM (Gibco) supplemented with 10% FBS (Hyclone), 100nM Dexamethasone (Sigma), 50μg/ml Ascorbic acid (Sigma), 40μg/ml Proline, 10ng/ml TGF-β3, 100μg/ml Sodium Pyruvate, 2mM Insulin-Transfer-S (Gibco), 53.5μg/ml Lindeic acid, Glycerophosphate (Sigma) and 12.5μg/ml BSA (Sigma). The medium was changed every 3 days. After 3 weeks, the cells were used for Alcian Blue staining assay and collected for qRT-PCR analysis.

Transplantation of MSCs into Immunocompromised Mice

The mouse model of *in vivo* bone formation by MSCs was performed as previously reported(Deng et al., 2016) with minor modification(Deng et al., 2016). All animal studies were approved (approval no. KT2016011-EC-1) by Laboratory Animal Center of Institute of Hematology & Blood Diseases Hospital, Chinese Academy of Medical Sciences & Peking Union Medical College (license no. SCXK & SYXK 2005-0001, Tianjin). Briefly, 2×10^6 hBM-MSCs or MC-MSCs derived from DOX-inducible GFP-MSX2 overexpressing H1 hESCs under DOX+SMs conditions at day 7 of hPSC-MSCs were mixed with hydroxyapatite/tricalcium phosphate (HA/TCP) scaffolds. Then, the hBM-MSCs and MC-MSCs were transplanted into the left and right dorsal subcutaneous sites of 6-week nude mice, respectively. 8 weeks later, all of the mice were euthanized and the transplants of each mouse were collected and fixed with 4% formaldehyde for 48h. The formed bone *in vivo* was identified by haematoxylin and eosin (H&E) staining and photographed by inverted microscope (Nikon).

Population Doublings Assay

MSCs derived from hPSCs and BM-MSCs were serially passaged in six-well plates for 10 passages. Briefly, cells cultured in six-well plates were trypsinized and re-cultured in a new six-well plate at 1.0×10^4 cells/cm^2 when they reached 90% confluency. Population doublings (PD) was calculated as follows(Meng et al., 2013; Umeda et al., 2015; Wei et al., 2012): NPD= (log10 [Np] - log10[Ns])/log10, where Np represents the harvested cell number before passage and Ns represents the seeded cell number at each passage, respectively.

Inflammatory Response of MSCs to IFNγ *in vitro*

To evaluate the inflammatory response of MSCs to inflammatory factor IFNγ, MC-MSCs or hBM-MSCs were detached with 0.05% trypsin and cultured in 24-well plate with DMEM-F12 (Gbico) containing 10% FBS, 1% L-Glutamine (Gibco) and 1% NEAA (Gibco). After being treated with 10ng/ml human IFN-γ (PeproTech) for 24h, the cells were collected for RNA isolation with TRIzol reagent (ThermoFisher). The expression level of inflammatory factors was detected by Real-time PCR with the SYBR reagent (Life) and 7900HT Fast Real-time PCR System (ThermoFisher). Primers for detection of IL-6, IDO1 and TGFβ were synthetized (Invitrogen) and listed in Table S2.

In Vitro Assays of T Cell Proliferation

The inhibition analysis of T lymphocyte proliferation by MSCs *in vitro* was performed as previously described(Wang et al., 2016) with minor modification. T lymphocytes were isolated from spleen and mesenteric lymph nodes of 6-week male C57 BL/6 mice. Briefly, the total cells were labelled with PE-CD3e (BioLegend) and T lymphocyte were enriched by fluorescence-activated cell sorter (BD FACSAria IIIu). Then, the sorted $CD3^+$ T lymphocytes were labeled with 5-μM FITC-CFSE (Invitrogen) 2×10^4 MC-MSC or BM-MSCs were mixed with 1×10^5 CFSE labelled $CD3^+$ T cells per well in a 96-well plateand cultured in 1640 basal medium (Gibco) supplemented with 10% FBS (Austria) at 100 μl/well.. For $CD4^+$ T lymphocyte proliferation analysis, the $CD3^+$ T cells were stimulated with plate-bound 1 μg/ml anti-CD3 antibody (BD Bioscience) and 2 μg/ml anti-CD28 antibody (eBioscience). For $CD8^+$ T lymphocyte proliferation analysis, the $CD3^+$ T cells were stimulated with molecular probes sulfate latex (Invitrogen). After stimulation for 72 h, the cells were harvested and stained with anti-CD4 and Anti-CD8 antibodies (BD Bioscience). The $CD4^+$ or $CD8^+$ population was gated for CFSE dilution analysis.

Mouse Model of Dextran Sulfate Sodium (DSS)-induced Colitis

The DSS-induced colitis model was performed as previously described(Wang et al., 2016). Briefly, 8-week male C57BL/6 mice were administered with drinking water containing 2% DSS (BioWest) for 6 days. 1×10^6 human BM-MSCs or MC-MSCs derived from hPSCs in PBS or PBS alone (a vehicle control) were injected i.p. into animals on day 2 and 3 after the start of the DSS treatment. The mice untreated drinking water were used as control. Body weight of the mice was monitored every day from

day 0 to day 13. The stool consistency, rectal bleeding and body weight (Wang et al., 2016) of mice were used to evaluate the severity of colitis as follows: 0, normal; 1, soft; 2, soft but formed; 3, liquid. All of the mice were euthanized at day 13. Upon necropsy, the colon of each mouse was dissected from each mouse and its length was measured. After being flushed with 1 × DPBS, the colons were fixed with 4% formaldehyde for at least 48h. The distal part of the colon was used for haematoxylin and eosin (H&E) staining.

MSC Potential Analysis of $CD271^{+}$ NCCs or $CD271^{-}$ cells from hPSCs

To evaluate the MSC potential of NCCs, the isolated $CD271^{+}$ NCCs or $CD271^{+}$ cells at day 3 of MSC induction were seeded into 12-well plates coated with growth factor-reduced gel (Stem Cell Technologies) at a density of 2×10^{4}/ml and cultured in DMEM/F12 media supplemented with 2% FBS, 4 ng/ml TGFβ1, 4 ng/ml bFGF, 0.5 μM CHIR99021 and 20 nM Decitabine) and 3 μg/ml DOX for 4 days. Then, the cells were dissociated into single cells using 0.05% Trypsin/EDTA and stained with the indicated antibodies (listed in Supplemental Table S1) in 0.2% BSA for FCM analysis.

Differentiation of hPSCs into Mesoendoderm-derived MSCs

Differentiation of hPSCs into mesoendoderm-derived MSCs was performed as previously reported(Tran et al., 2012) with minor modification. Briefly, to induce mesoendoderm, hPSCs clumps were seeded onto GFR-coated dishes in Custom TeSR (Stem Cell Tech) containing 5ng/ml Activin A (Peprotech), 2μM BIO (Sigma) and 20ng/ml BMP4 (Peprotech) for 3 days. Then, the derived cells were further induced into MSCs with MSC-induction medium containing 90% α-MEM (Gibco), 10% FBS (Hyclone), 10ng/ml EGF (Peprotech) and 10ng/ml bFGF (Peperotech) for 10 days. Finally, the cells were dissociated into single cells using 0.05% Trypsin/EDTA and stained with the indicated antibodies (listed in Supplemental Table S1) in 0.2% BSA for FCM analysis.

Differentiation of hPSCs into Trophoblast-derived MSCs (TB-MSCs)

Differentiation of hPSCs into mesoderm-derived MSCs was performed as previously reported(Wang et al., 2016) with minor modification. Briefly, for trophoblast (TB) induction, hPSCs clumps were seeded onto GFR-coated dishes in Custom TeSR (Stem Cell Tech) containing 10μM SB431542 (Selleck) and 10ng/ml BMP4 (Peprotech) for 7 days. Then, the derived cells were further induced into MSCs with 80%

α-MEM (Gibco), 20% FBS (Hyclone), 1×NEAA(Gibco) and 1% L-Glutamine (Gibco) for 6 days. Finally, the cells were dissociated into single cells using 0.05% Trypsin/EDTA and stained with the indicated antibodies (listed in Supplemental Table S1) in 0.2% BSA for FCM analysis.

Bioinformatics Analysis

Heatmap was generated using HemI heatmap illustrator software (GPS) or R language. Gene ontology (GO) was conducted using the online tool (http://geneontology.org/ or http://david.abcc.ncifcrf.gov/). Pathway enrichment analysis was performed using Ingenuity Pathway Analysis (IPA 1.0 version) software or KEGG website (http://www.kegg.jp/). Principal component analysis (PCA) were completed in ClustVis website (http://biit.cs.ut.ee/clustvis/). The Venn map was generated in Venny 2.1 website (http://bioinfogp.cnb.csic.es/tools/venny/index.html).

Lentivirus Production

For knockdown of gene expression, shRNAs targeting MSX2, TWIST1, PRAME and SOX10 were cloned into pLKO.1 vector (Addgene), while a scramble shRNA (Scr) was used as a control. For overexpression of genes, the coding sequence of PRAME or TWIST1 was inserted into psin-EF1a-GFP vector. The lentivirus was packed and collected as described previously(Wu et al., 2015). The sequences of shRNAs are listed in Supplemental Table S2.

Construct Generation

For sgRNA-mediated MSX2 knockout by CRISPR/Cas9 system, lentivirus-based CRISPR-Cas9-Lenti-V2 vector containing sgRNAs designed with the CRISPR design website (http://tools.genome-engineering.org) were used.

For shRNA-mediated MSX2, TWIST1, PRAME and SOX10 knock-down, lentivirus-based pLKO.1 vectors (Addgene) containing various shRNA target sequences (Sigma-Aldrich) (See Supplemental Table S2) were used to package virus according to the manufacturer's instructions (Viralpower Lentivirus Packaging System, Invitrogen).

Construct carrying PRAME were generated from the coding sequence of PRAME (from cDNA of H1 hESC-derived MSC-like cells by exogenous MSX2 overexpression for 7 days). The coding sequence was finally cloned into SpeI and BmtI double digested psin-EF1a-GFP vector for further

experiment.

The reporter constructs of pTWIST1-0.7kb-LUC and pTWIST1-1.4kb-LUC were cloned from H1 hESC genomic DNA, then cloned into Xho I and Hind III double digested pGL3 vector (Promega). The two MSX2 binding sites (MBS), MBS1 (CCAATGAC) and MBS2 (CGAATTGT) on pTWIST1-0.7kb-LUC construct were predicted by JASPAR website (http://jaspar.genereg.net/cgi-bin/jaspar_db.pl). The pTWIST1-0.7kb-LUC construct containing MSX2 binding-site (MBS) mutants were generated by recombinant PCR. Primer sequences used for construct generation are provided in Supplemental Table S2.

Reporter Assay

For MSX2 target analysis, HEK-293T cells, H1 hESCs or BC1 hiPSCs were co-transfected with 0.5μg of pTWIST1-LUC reporter construct and 0.5μg of psin-EF1a-MSX2 using FuGene HD transfection reagent (Promega). 0.05μg of Renilla plasmid was co-transfected as an internal control. 48 h or 72 h after transfection, cells were harvested for luciferase activity measurement. Luciferase activity was determined using Dual-Luciferase assay (Promega) according to the Manufacturer's Instruction. All assays were carried out in triplicate. Details for the construction of pTWIST1-LUC, MSX2 binding site mutants are available in Supplemental Table S2.

Chromatin immunoprecipitation (ChIP) assay

3×10^7 GFP-MSX2 H1 hESCs were collected for ChIP assay after treatment with treated with DOX (3μg/ml) for 7 days. ChIP assay was performed using the Magna ChIP™ A/G kit (Millipore) following the Manufacturer's Instructions. The primers designed to detect the enrichment of the fragments are available in Supplemental Information, Supplemental Table S2.

Supplemental References

Deng, P., Zhou, C., Alvarez, R., Hong, C., and Wang, C.Y. (2016). Inhibition of IKK/NF-kappaB Signaling Enhances Differentiation of Mesenchymal Stromal Cells from Human Embryonic Stem Cells. Stem cell reports *6*, 456-465.

Lu, S., Ge, M., Zheng, Y., Li, J., Feng, X., Feng, S., Huang, J., Feng, Y., Yang, D., Shi, J.*, et al.* (2017). CD106 is a novel mediator of bone marrow mesenchymal stem cells via NF-kappaB in the bone marrow failure of acquired aplastic anemia. Stem cell research & therapy *8*, 178.

Meng, X., Su, R.J., Baylink, D.J., Neises, A., Kiroyan, J.B., Lee, W.Y., Payne, K.J., Gridley, D.S., Wang, J., Lau, K.H.*, et al.* (2013). Rapid and efficient reprogramming of human fetal and adult blood CD34+ cells into mesenchymal stem cells with a single factor. Cell research *23*, 658-672.

Tran, N.T., Trinh, Q.M., Lee, G.M., and Han, Y.M. (2012). Efficient differentiation of human pluripotent stem cells into mesenchymal stem cells by modulating intracellular signaling pathways in a feeder/serum-free system. Stem cells and development *21*, 1165-1175.

Umeda, K., Oda, H., Yan, Q., Matthias, N., Zhao, J., Davis, B.R., and Nakayama, N. (2015). Long-term expandable SOX9+ chondrogenic ectomesenchymal cells from human pluripotent stem cells. Stem cell reports *4*, 712-726.

Vodyanik, M.A., Yu, J., Zhang, X., Tian, S., Stewart, R., Thomson, J.A., and Slukvin, II (2010). A mesoderm-derived precursor for mesenchymal stem and endothelial cells. Cell stem cell *7*, 718-729.

Wang, X., Lazorchak, A.S., Song, L., Li, E., Zhang, Z., Jiang, B., and Xu, R.H. (2016). Immune modulatory mesenchymal stem cells derived from human embryonic stem cells through a trophoblast-like stage. Stem cells *34*, 380-391.

Wei, H., Tan, G., Manasi, Qiu, S., Kong, G., Yong, P., Koh, C., Ooi, T.H., Lim, S.Y., Wong, P.*, et al.* (2012). One-step derivation of cardiomyocytes and mesenchymal stem cells from human pluripotent stem cells. Stem cell research *9*, 87-100.

Wu, Q., Zhang, L., Su, P., Lei, X., Liu, X., Wang, H., Lu, L., Bai, Y., Xiong, T., Li, D.*, et al.* (2015). MSX2 mediates entry of human pluripotent stem cells into mesendoderm by simultaneously suppressing SOX2 and activating NODAL signaling. Cell research *25*, 1314-1332.

Zhang, X., Yang, Y., Zhang, L., Lu, Y., Zhang, Q., Fan, D., Zhang, Y., Zhang, Y., Ye, Z., and Xiong, D. (2017). Mesenchymal stromal cells as vehicles of tetravalent bispecific Tandab (CD3/CD19) for the treatment of B cell lymphoma combined with IDO pathway inhibitor D-1-methyl-tryptophan. Journal of hematology & oncology *10*, 56.
