# Supplementary material for: MSX2 Initiates and Accelerates Mesenchymal Stem/Stromal Cell Specification of hPSCs by Regulating TWIST1 and PRAME
Source: Stem Cell Reports. 2018 Jul 19;11(2):497–513. doi: 10.1016/j.stemcr.2018.06.019 (PMC6092836; doi:10.1016/j.stemcr.2018.06.019)
Supplement: Document S2. Article plus Supplemental Information [file mmc4.pdf]

Stem Cell Reports
Article
OPEN ACCESS

MSX2 Initiates and Accelerates Mesenchymal Stem/Stromal Cell Specification of hPSCs by Regulating TWIST1 and PRAME

Leisheng Zhang,[1,2,4] Hongtao Wang,[1,2,4] Cuicui Liu,[1,2] Qingqing Wu,[1,2] Pei Su,[1,2] Dan Wu,[1,2] Jiaojiao Guo,[3] Wen Zhou,[3] Yuanfu Xu,[1,2] Lihong Shi,[1,2] and Jiaxi Zhou[1,2,*]
[1]State Key Laboratory of Experimental Hematology, Institute of Hematology & Blood Diseases Hospital, Chinese Academy of Medical Sciences & Peking Union Medical College, Tianjin 300020, China
[2]Center for Stem Cell Medicine, Chinese Academy of Medical Sciences & Department of Stem Cells and Regenerative Medicine, Peking Union Medical College, Tianjin 300020, China
[3]School of Basic Medical Science and Cancer Research Institute, Central South University, Changsha 410013, China
[4]Co-first author
*Correspondence: zhoujx@ihcams.ac.cn
https://doi.org/10.1016/j.stemcr.2018.06.019

SUMMARY

The gap in knowledge of the molecular mechanisms underlying differentiation of human pluripotent stem cells (hPSCs) into the mesenchymal cell lineages hinders the application of hPSCs for cell-based therapy. In this study, we identified a critical role of muscle segment homeobox 2 (MSX2) in initiating and accelerating the molecular program that leads to mesenchymal stem/stromal cell (MSC) differentiation from hPSCs. Genetic deletion of MSX2 impairs hPSC differentiation into MSCs. When aided with a cocktail of soluble molecules, MSX2 ectopic expression induces hPSCs to form nearly homogeneous and fully functional MSCs. Mechanistically, MSX2 induces hPSCs to form neural crest cells, an intermediate cell stage preceding MSCs, and further differentiation by regulating TWIST1 and PRAME. Furthermore, we found that MSX2 is also required for hPSC differentiation into MSCs through mesendoderm and trophoblast. Our findings provide novel mechanistic insights into lineage specification of hPSCs to MSCs and effective strategies for applications of stem cells for regenerative medicine.

INTRODUCTION

Mesenchymal stem/stromal cells (MSCs) are promising sources for cell-based therapies due to their self-renewal capacity, multi-lineage differentiation potential, and immunomodulatory properties (Friedenstein et al., 1968; Nombela-Arrieta et al., 2011). There are more than 300 clinical trials underway to evaluate the utility of MSCs in a variety of diseases, including autoimmune disorders, wound healing, and neurological disorders (Keating, 2012; Salem and Thiemermann, 2010). Currently, bone marrow-derived MSCs (BM-MSCs) are the most commonly used source for MSCs in clinical trials (Ankrum and Karp, 2010). However, these cell sources have some limitations, including limited cell proliferative capacity, declined therapeutic potency after *in vitro* expansion, donor-dependent variability in quality, and the risk of pathogen transmission (Wang et al., 2016). These shortcomings hamper their clinical applications. Therefore, there is an urgent need to find alternative inexhaustible sources of MSCs.

Human pluripotent stem cells (hPSCs), including human embryonic stem cells (hESCs) and human induced pluripotent stem cells (hiPSCs), have the capacity to self-renew indefinitely and give rise to almost all human cell types (Lund et al., 2012) and therefore have emerged as an alternative source for MSCs. Considerable progress has been made in differentiating hPSCs into MSCs with immune-phenotype and biological functions similar to those of BM-MSCs (Kimbrel et al., 2014; Wang et al., 2014). The use of hPSCs as a source for MSCs has many advantages, including generating unlimited amounts of early-passage MSCs with consistent high quality and deriving patient-derived induced pluripotent stem cells (iPSCs) for autologous therapy through gene correction (Frobel et al., 2014; Sabapathy and Kumar, 2016).

Since 2005, several groups have developed a number of protocols to differentiate hPSCs into MSCs with an immunophenotype and biological function similar to those of BM-MSCs. These methods include OP9 co-culture (Barberi et al., 2005; Olivier et al., 2006), three-dimensional embryoid body (EB) induction (Brown et al., 2009; Wei et al., 2012), and differentiation on two-dimensional monolayer (Gonzalo-Gil et al., 2016; Harkness et al., 2011). Despite these encouraging advances, limitations remain in the existing protocols. For example, most strategies require laborious manipulations, which include scraping, handpicking, sorting of cells, or serial passages (Fukuta et al., 2014; Gibson et al., 2017; Kopher et al., 2010; Lian et al., 2007; Sanchez et al., 2011). In addition, the current differentiation procedures are time consuming and usually take several weeks to obtain homogeneous MSCs (Boyd et al., 2009; Wang et al., 2016). Thus, the development of simple, rapid, and efficient methods directing the differentiation of hPSCs into MSCs becomes crucial.

In contrast to the advances in the development of differentiation strategies, little is known about the molecular

This is an open access article under the CC BY license (http://creativecommons.org/licenses/by/4.0/).

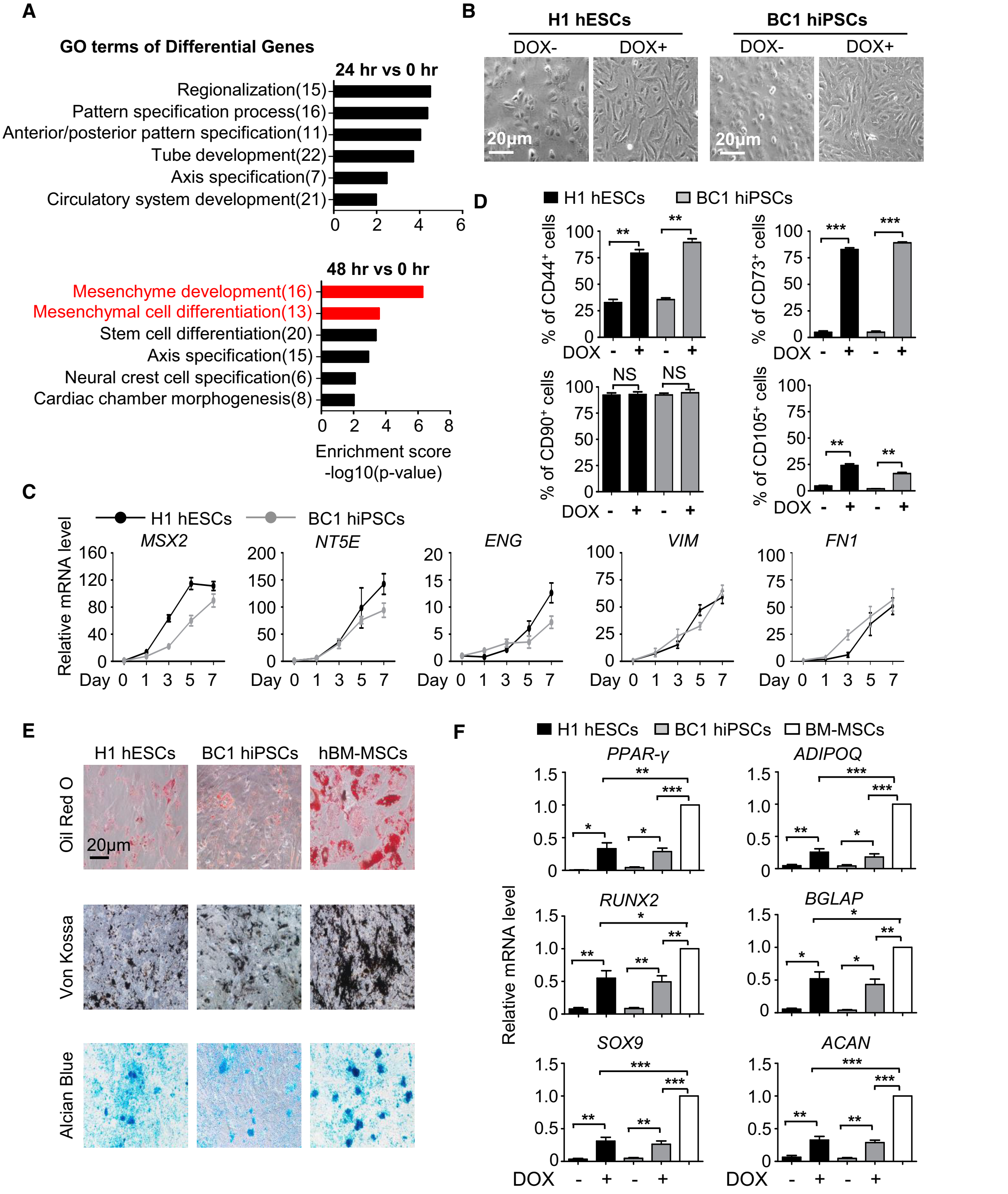

Figure 1. MSX2 Initiates Mesenchymal Differentiation in hPSCs

(A) Gene ontology (GO) analysis of upregulated genes in DOX-inducible GFP-MSX2 H1 hESCs with 3 μg/mL DOX.

(B) Images of GFP-MSX2 hPSCs (H1, BC1) cultured in DMEM/F12 containing 2%FBS ± DOX (3 μg/mL) for 7 days. Scale bar, 20 μm.

(C) qRT-PCR analysis of MSX2 and MSC markers in GFP-MSX2 hPSCs (H1, BC1) with 3 μg/mL DOX (mean ± SEM, N = 3). Values are normalized to day 0 (=1) before adding DOX.

(D) Flow cytometry (FCM) analysis of MSC markers of GFP-MSX2 hPSCs (H1, BC1) cultured in DMEM/F12 containing 2%FBS ± DOX (3 μg/mL) for 7 days (mean ± SEM, N = 3). $^{**}p < 0.01$; $^{***}p < 0.001$; NS, not significant.

(legend continued on next page)

signatures and mechanisms underlying the differentiation process (Deng et al., 2016; Luzzani and Miriuka, 2017). This can be largely attributed to the fact that most differentiation methods require several weeks to generate homogeneous MSCs from hPSCs, making it unfeasible to dissect the underlying molecular program. Recently, it was reported that inhibition of nuclear factor kappa B (NF-kB) signaling or EZH2 enhances differentiation of hPSCs to MSCs (Deng et al., 2016; Yu et al., 2017). Inhibition of transforming growth factor β (TGF-β) signaling with SB431542 also enhances the generation of MSCs (Fukuta et al., 2014; Mahmood et al., 2010). Besides these studies, little is known about the molecular mechanism for MSC differentiation. Thus, it is of great importance to establish an improved model for dissecting the molecular mechanism underlying hPSC differentiation toward MSCs. In this study, by combining MSX2 ectopic expression with a soluble-molecule (SM) cocktail, we developed a rapid and efficient strategy to generate near-homogeneity in MSCs from hPSCs within a week. The MSCs are functional and display multi-lineage differentiation potential and function in preventing colitis *in vivo* comparable with that of BM-MSCs. By conducting transcriptomic analysis, we uncovered multiple key signaling pathways and molecules involved in MSC differentiation from hPSCs. Furthermore, we identified TWIST1 and PRAME as crucial regulators of MSC differentiation.

RESULTS

MSX2 Initiates Mesenchymal Differentiation in hPSCs

We recently reported that MSX2 mediates the entry of hPSCs into mesendoderm during early fate specification (Wu et al., 2015). From the RNA sequencing (RNA-seq) data of hPSCs with MSX2 ectopic expression, we found rapid upregulation of multiple mesenchyme development and mesenchymal cell differentiation-associated genes in cells 48 hr and 72 hr after MSX2 overexpression, even under pluripotency-supporting conditions (Figures 1A and S1A). In contrast, early pattern specification and regionalization-associated genes were enriched mainly 24 hr after MSX2 overexpression (Figure 1A). These observations led us to speculate that MSX2 itself might be capable of initiating mesenchymal differentiation in hPSCs. To test this, we took advantage of a previously described DOX-inducible system to induce MSX2 ectopic expression under basal medium (DMEM/F12) containing 2% FBS, 1% L-glutamine (Gibco), and 1% non-essential amino acid (NEAA) (Gibco) known to support mesenchymal cells and then determined whether mesenchymal differentiation could be induced (Boyd et al., 2009). Overexpression of MSX2 in hPSCs (H1, H9, BC1, and Z-15), which could be monitored by the emergence of GFP fluorescence (Figure S1B), caused profound morphological changes, including from aggregates of cobblestone-shaped cells to separated cells with elongated, spindle-like shapes (Figures 1B and S1C), highly reminiscent of MSCs. To further characterize those cells, we measured the expression of CD44, CD73, CD90, CD105, and other markers of MSCs with qRT-PCR and flow cytometry assays (Barberi et al., 2005; Chen et al., 2012; Mani et al., 2008). Indeed, a gradual upregulation of NT5E (also known as CD73), ENG (also known as CD105), VIM, and FN1, and the concomitant sharp decrease of pluripotency markers, including POU5F1 (also known as OCT4), SOX2, and NANOG, were observed (Figures 1C and S1D). With MSX2 ectopic expression, almost 90% of cells were positive for CD44, CD73, and CD90 within 7 days of induction (Figures 1D, S1E, and S1F). In contrast, little expression of CD31, CD34, and CD45 was detected, indicating that the cells are neither endothelial nor hematopoietic cells (Figure S1E). Compared with MSCs from the bone marrow, CD105 expression levels were lower in MSX2 overexpressed cells (Figures 1D and S1F). This is an interesting distinction between MSX2-induced MSCs and those from the bone marrow (BM-MSCs).

We next determined the multi-lineage differentiation potential of the cells denoted as MSX2 programmed cells (M-MSCs), including to adipogenic, osteogenic, and chondrogenic cells (Zhang et al., 2017). After 3–4 weeks of differentiation, a portion of cells were stained positive for oil red O, von Kossa, and alcian blue, respectively (Figure 1E). However, compared with BM-MSCs, the differentiation potential of the MSX2-overexpressing cells was much lower. Experiments with qRT-PCR analysis of the multiple lineage differentiation markers (Vodyanik et al., 2010), including PPAR-γ, ADIPOQ, RUNX2, BGLAP, SOX9, and ACAN, further confirmed the observations (Figure 1F). Thus, although MSX2 ectopic expression suffices to induce entry of hPSCs to the mesenchymal fate, the differentiated cells appear to be immature and only exhibit some degree of adipogenic, osteogenic, and chondrogenic potential and partial expression of CD105 compared with BM-MSCs.

(E) Adipogenic, osteogenic, or chondrogenic differentiation potential of the MSCs derived from GFP-MSX2 hPSCs (H1, BC1) and human bone marrow-derived (hBM) MSCs for the indicated lineages. Scale bar, 20 μm.
(F) qRT-PCR analysis of adipogenic (upper), osteogenic (middle), chondrogenic (bottom) markers after induction for the indicated lineages (mean ± SEM, N = 3). *p < 0.05; **p < 0.01; ***p < 0.001.
See also Figure S1.

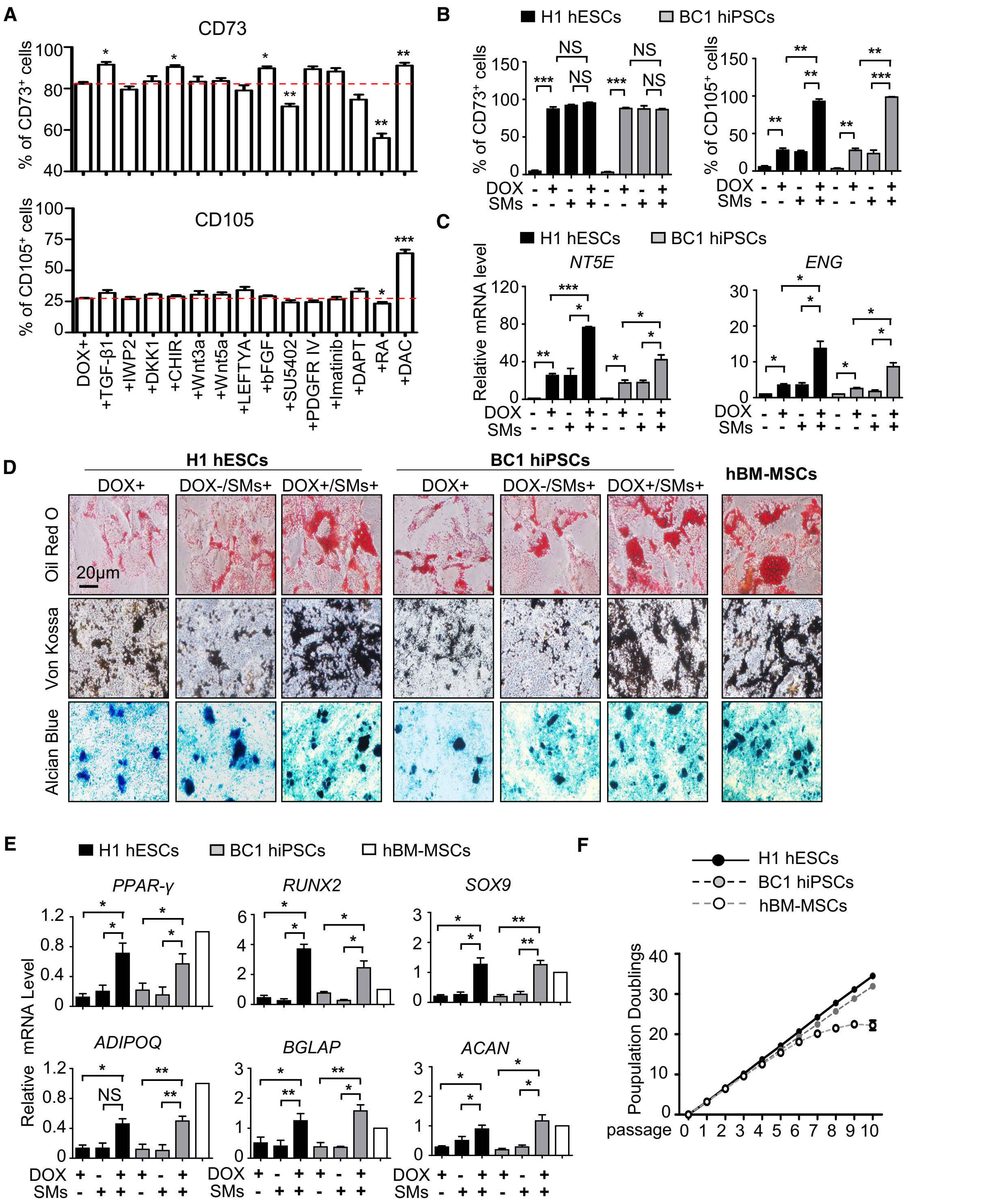

Figure 2. Rapid and High-Efficiency Derivation of MSCs

(A) FCM analysis for MSC markers of GFP-MSX2 H1 hESCs with 3 μg/mL DOX alone or indicated chemical compound addition (see Table S3) for 7 days (mean ± SEM, N = 3). $*p < 0.05$; $**p < 0.01$; $***p < 0.001$.

(B) FCM analysis of MSC markers in GFP-MSX2 hPSCs (H1, BC1) with indicated treatments for 7 days (mean ± SEM, N = 3). $**p < 0.01$; $***p < 0.001$; NS, not significant.

(legend continued on next page)

Rapid and High-Efficiency Derivation of MSCs

We and others have shown that chemical compounds are powerful tools for large-scale derivation of progenitor cells and terminal differentiated functional cells from hPSCs (Loh et al., 2016; Yang et al., 2016; Zhou et al., 2010). To identify conditions that allow for the development of more mature MSCs, we conducted a small-scale screening of chemical compounds and growth factors associated with the Wnt, fibroblast growth factor (FGF), TGF-βsignaling pathways as well as several known epigenetic regulators, all of which have been implicated in mesenchymal morphogenesis (Meng et al., 2013; Nieto et al., 2016; Thiery et al., 2009; Williams and Hare, 2011). The screening experiments allowed us to identify TGF-β1, CHIR99021, basic FGF (bFGF), and DAC (decitabine), a chemical compound that inhibits DNA methyltransferase, which enhanced the generation of $CD44^+$, $CD73^+$, or $CD105^+$ cells when combined with MSX2 ectopic expression (Figures 2A and S2A–S2C). We subsequently applied all four SMs to MSX2-overexpressing cells (MC-MSCs) and found that the derived MC-MSCs showed typical elongated, spindle-like shapes more strikingly (Figure S2D). Moreover, $CD73^+$ or $CD105^+$ cells could be derived nearly homogeneously, much higher than MSX2 ectopic expression and growth factors/chemical compound treatment alone (Figures 2B and S2E–S2G). Consistent with these findings, the levels of NT5E, ENG, VIM, and FN1 mRNA were much higher in MC-MSCs (Figures 2C and S2H). In contrast, little difference of CD44, CD90, was detected between the different groups (Figure S2E). Furthermore, the colony-forming ability of MC-MSCs was much higher than that of M-MSCs and SM-MSCs, reaching almost the same level as BM-MSCs (Figure S2I).

We next examined the adipogenic, osteogenic, and chondrogenic potential of MC-MSCs. Indeed, the differentiation potential was significantly improved over the M-MSCs (Figure 2D), reaching the same level as BM-MSCs. Furthermore, elevated mRNA levels of adipogenic markers including PPAR-γ and ADIPOQ, osteogenic markers including RUNX2 and BGLAP, and chondrogenic markers including SOX9 and ACAN were also observed in MC-MSCs after differentiation into respective lineages (Figure 2E). Additionally, like BM-MSCs, MC-MSCs were capable of forming bone *in vivo* (Figure S2J). Importantly, unlike BM-MSCs, which have limited proliferation potential (Wei et al., 2012), the MC-MSCs could be cultivated consecutively for more than 10 passages (Figure 2F). In summary, MSX2 ectopic expression, aided with a cocktail of small molecules and soluble factors, allows us to accomplish rapid and near-homogeneous derivation of mature MSCs from hPSCs.

MC-MSCs Resemble BM-MSCs and Are Functional

To further characterize MC-MSCs at a molecular level, we conducted genome-wide RNA profiling to compare MC-MSCs with BM-MSCs. Unsupervised clustering revealed grouping of MC-MSCs with BM-MSCs (Figure 3A). Furthermore, MC-MSCs have much more similarities in global gene expression to BM-MSCs as opposed to M-MSCs or hPSCs (Figure 3B). Expectedly, multiple pluripotency-associated genes showed minimal expression in MC-MSCs, while mesenchymal development/differentiation-associated genes were highly expressed with levels comparable with those in BM-MSCs (Figure 3B). Gene set enrichment analysis (GSEA) also showed high enrichment of genes, including mesenchymal cell markers and genes involved in mesenchymal cell differentiation, mesenchyme development, and positive regulation of mesenchymal cell proliferation in MC-MSCs (Figures 3C and S3A).

The similarities between MC-MSCs and BM-MSCs led us to ask whether MC-MSCs are functional. We first examined whether MC-MSCs exhibit immunomodulatory activity *in vitro*. After interferon γ treatment for 24 hr, the expression of anti-inflammatory gene IDO1 and pre-inflammatory gene IL-6 dramatically elevated in both MC-MSCs and BM-MSCs, while TGF-β expression was mostly affected (Figure S3B). Furthermore, like BM-MSCs, MC-MSCs inhibited proliferation of $CD4^+$ T lymphocytes when stimulated with anti-CD3 and anti-CD28 antibody and $CD8^+$ T lymphocytes when stimulated with Molecular Probes sulfate latex (Figure 3D). To further explore the immunomodulatory activity of MC-MSCs *in vivo*, we took advantage of a recently described dextran sulfate sodium (DSS)-induced acute colitis model (Wang et al., 2016) to assess whether colitis-caused tissue damage and decrease in body weight could be treated with MC-MSCs. In this

(C) qRT-PCR analysis of MSC markers in GFP-MSX2 hPSCs (H1, BC1) with indicated treatments for 7 days (mean ± SEM, N = 3). $^*p < 0.05$; $^{**}p < 0.01$; $^{***}p < 0.001$. Values are normalized to the Dox− group (=1).
(D) Tri-lineage differentiation potential of MC-MSCs derived from GFP-MSX2 hPSCs (H1, BC1) and hBM-MSCs for the indicated lineages. Scale bar, 20 μm.
(E) Relative expression levels of genes associated with tri-lineage differentiation of the MC-MSCs derived from GFP-MSX2 hPSCs (H1, BC1) (mean ± SEM, N = 3). $^*p < 0.05$; $^{**}p < 0.01$. Values are normalized to the hBM-MSCs group (=1).
(F) Expansion potential of MC-MSCs derived from GFP-MSX2 hPSCs (H1, BC1) and hBM-MSCs in MSC culture media with CHIR99021 (0.5 μM) for 10 passages by population doubling assay (mean ± SEM, N = 3).
See also Figure S2.

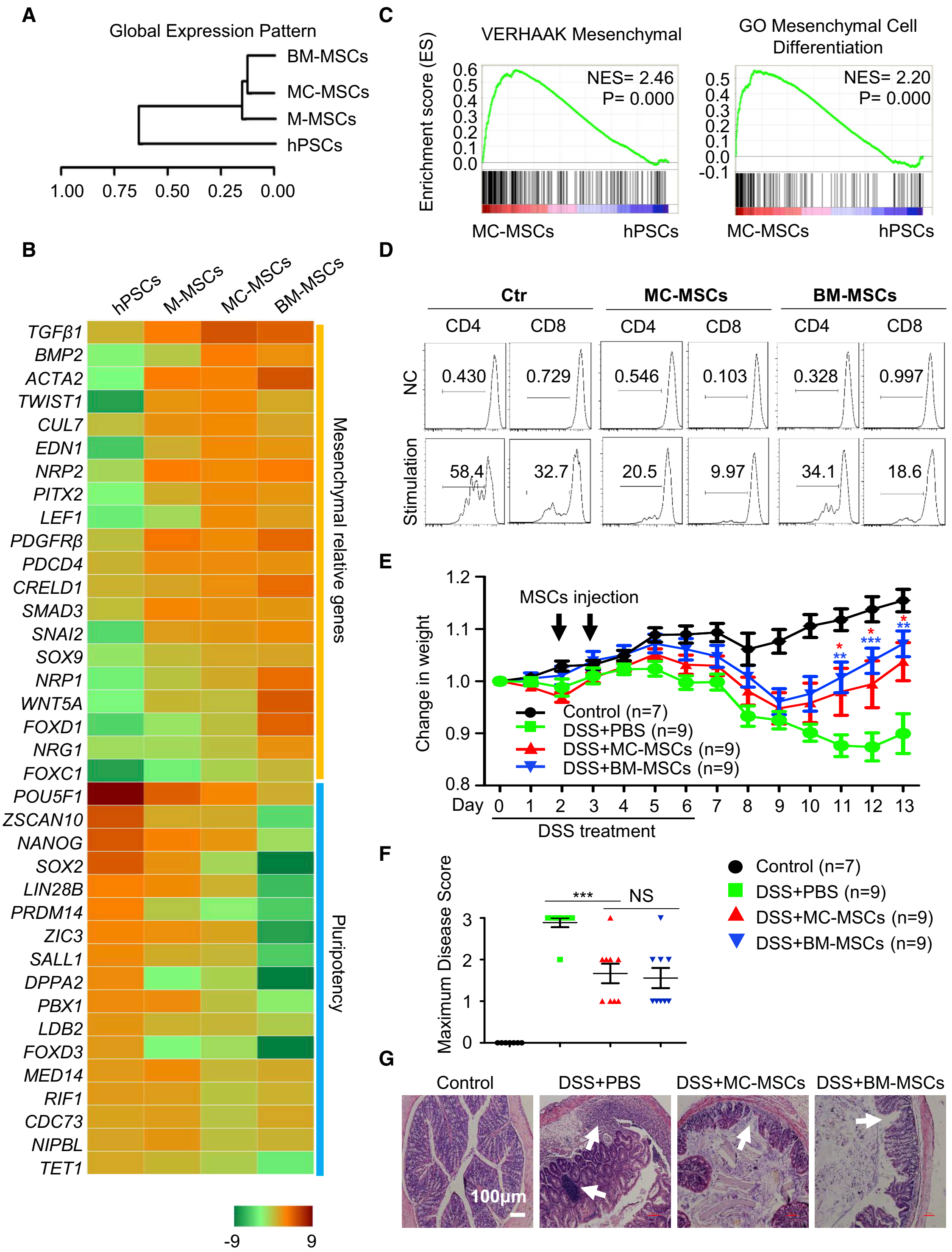

Figure 3. MC-MSCs Resemble BM-MSCs and Are Functional

(A) Hierarchical clustering analysis of hPSCs (H1 hESCs), M-MSCs, MC-MSCs, BM-MSCs.

(B) Heatmap illustrating expression of mesenchymal development/differentiation and pluripotency-associated genes for hPSCs (H1 hESCs), M-MSCs, MC-MSCs, BM-MSCs.

(C) GSEA comparing MC-MSCs and hPSCs (H1 hESCs). The NES and p values are shown.

(legend continued on next page)

model, the decrease of body weight occurred in the window between day 4 and day 9 after DSS treatment, and the mice soon began to gain weight from day 10 after application of MC-MSCs or BM-MSCs (Figure 3E). The maximum weight loss measurement also showed significant improvement after MC-MSC treatment (Figure S3C, $p < 0.05$). No significant difference was found between the groups of MC-MSCs or BM-MSCs. The maximum disease score was much lower after MC-MSC treatment (Figure 3F, $p < 0.001$). Also, application of MC-MSCs prevented the decrease of colon length (Figures S3D and S3E, $p < 0.001$), while no significant difference was seen between animals treated with MC-MSCs or BM-MSCs. Compared with the DSS + PBS group, MC-MSCs reduced the maximum severity of DSS-induced colitis (Figure 3F). As expected, less damage to the epithelial cells was also observed after MC-MSC treatment (Figure 3G). Thus, MC-MSCs exhibit healing effects similar to BM-MSCs in the *in vivo* colitis model and therefore can be potentially used as tools for cell-based therapy and other regenerative medicine-related purposes.

Neural Crest as the Intermediate Stage between Pluripotency and Mesenchymal Fate

The high-efficiency and rapid directed differentiation method we have developed makes it feasible to dissect how cell fate changes occur from the pluripotent state to the mesenchymal fate. We conducted time course RNA-seq analysis of cells undergoing the fate changes (i.e., from day 0 to day 7). To elucidate the differentiation route, 2,782 highly expressed genes at different time points were clustered by using hierarchical cluster analysis (Figure 4A). Consistent with the initial loss of pluripotency and onset of early differentiation, genes associated with primary germ layer formation were highly enriched in the cell population of day 1 (Figure 4A). Overall, the principal component analysis (PCA) of the transcriptome showed a clear stepwise differentiation process from pluripotency to mesenchymal cells (Figure 4B).

Where do mesenchymal cells originate during early human development? There have been different reports suggesting that they might arise from mesoderm, endoderm, or the neural crest based on *in vivo* or *in vitro* models (Fukuta et al., 2014; Motohashi et al., 2007; Slukvin and Vodyanik, 2011; Vodyanik et al., 2010). Recently, trophoblasts were reported as the potential origin for MSCs during hPSC differentiation (Wang et al., 2016). To explore this in our differentiation model, we clustered those 14,453 differentially expressed genes into different categories and tested their potential overlaps using Venn map analysis. Surprisingly, no overlap of MSC-associated genes with trophoblasts was found (0/5) (Figure 4C). In contrast, neural crest-associated genes exhibited nearly perfect overlap with those of MSCs (39/40) (Figure 4C), suggesting that the neural crest may be the main intermediate stage during MSC induction in our method. To test this, we explored the dynamic expression of neural crest-related genes during MSC induction. The results from RNA-seq showed that neural crest-associated genes quickly upregulated at day 1 of MSC induction, peaked at day 3, and then began to decrease (Figure 4D). Real-time PCR analysis of the neural crest-related genes, including SOX9, SOX10, FOXD3, and CD271, further confirmed these observations (Figure 4E). Consistently, flow cytometry analysis showed that $CD271^+$ cells began to appear at day 1 of MSC induction, peaked at day 3, became $CD73^+$ cells at day 5, and disappeared at day 7 (Figure S4A). Importantly, the isolated CD271-positive cells at day 3 of MSC induction could further differentiate to MSCs (Figure 4F). Furthermore, knockdown of SOX10, a master regulator of neural crest genesis (Gammill and Bronner-Fraser, 2003), severely impaired neural crest induction and MSC induction (Figures 4G–4I), strongly indicating that neural crest serves as the main intermediate stage during the cell fate transition from pluripotency to mesenchymal cells. Interestingly, we noticed that the isolated $CD271^-$ cells could also partially differentiate into MSCs (Figure S4B). The increase of mesendoderm-associated genes T and MIXL1 was observed (Figures 4D and S4C), although their expression

(D) The sorted $CD3^+$ T lymphocytes were stimulated with plate-bound anti-CD3 antibody and anti-CD28 antibody or with Molecular Probes sulfate latex for 72 hr. Then, the lymphocytes were stained with anti-CD4 or anti-CD8 antibodies for CFSE dilution analysis. One of three independent experiments is shown. Ctr, control.

(E) Mice were given untreated drinking water (control) or 2% DSS in drinking water (DSS) for 6 days (Wang et al., 2016). Then, all mice were given untreated drinking water for the next 7 days. On days 2 and 3, mice treated with DSS were injected intraperitoneally (i.p.) with PBS, MC-MSCs or BM-MSCs. The control group mice were injected i.p. with PBS. The change in body weight of mice was measured. Data are analyzed by multiple t test and shown as mean ± SEM (N = 3). *p < 0.05; **p < 0.01, ***p < 0.001.

(F) The maximum colitis severity of each animal in the four groups was quantified by maximum disease score, which is typically assessed based on the stool consistency, rectal bleeding, and body weight together (Wang et al., 2016). Data are analyzed by students' t test and shown as mean ± SEM. ***p < 0.001; NS, not significant.

(G) Histopathologic analysis of colons by H&E staining. The white arrows indicate typical morphology of the damage. Scale bar, 100 μm. See also Figure S3.

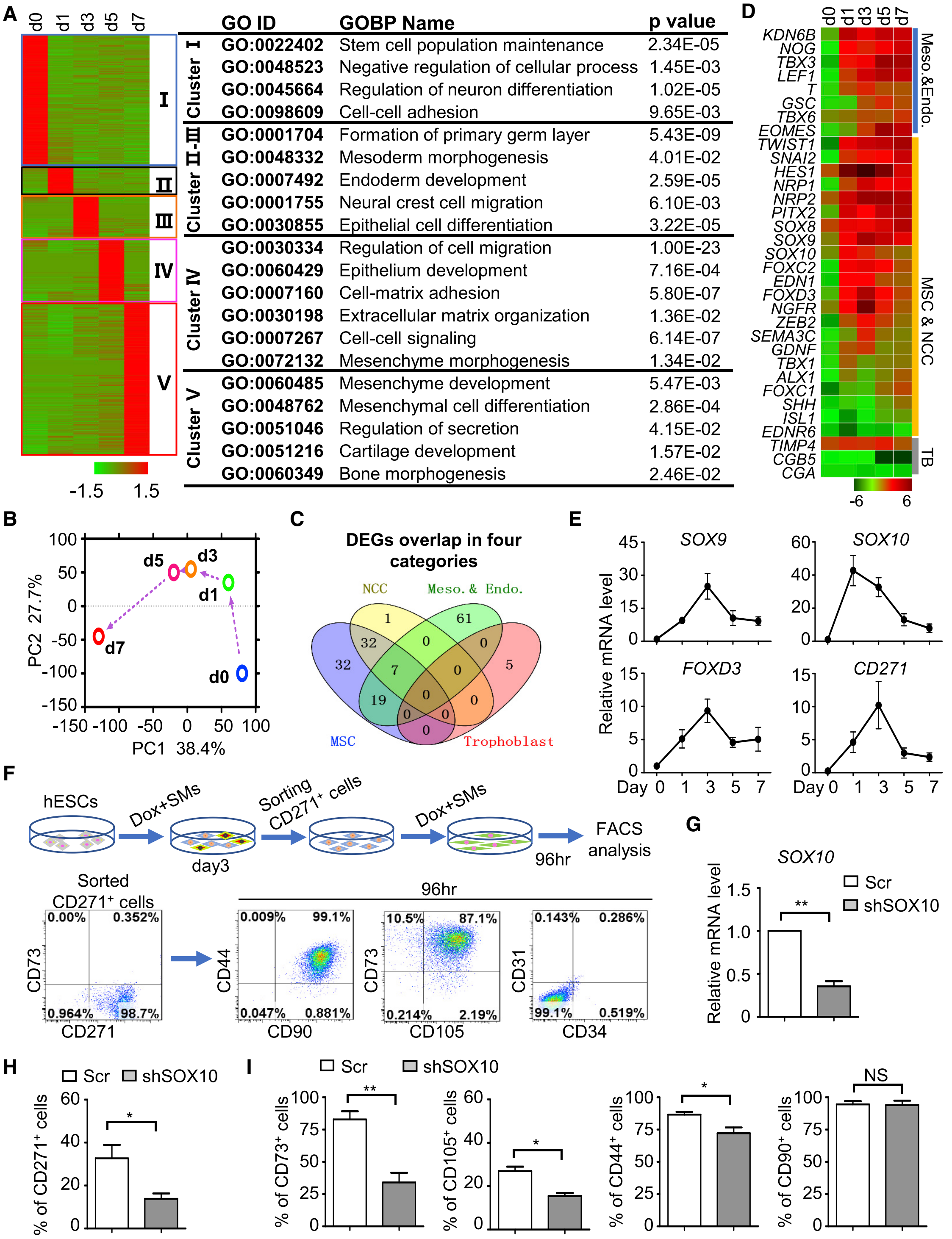

Figure 4. Neural Crest as the Intermediate Stage between Pluripotency and Mesenchymal Fate

(A) Hierarchical cluster analysis of 2,782 differentially expressed genes (DEGs) (fold change >2, fragments per kilobase of transcript per million mapped reads >0.2) in whole-transcriptome level (left) and GO biological process (GOBP) analysis with p value (right).

(B) PCA of samples of MC-MSC induction from GFP-MSX2 H1 hESCs. PC1, principal component 1; PC2, principal component 2.

(C) Venn diagram shows the overlap among MSC, NCC, mesoderm and endoderm, and trophoblast-associated genes enriched in 14,453 DEGs.

(legend continued on next page)

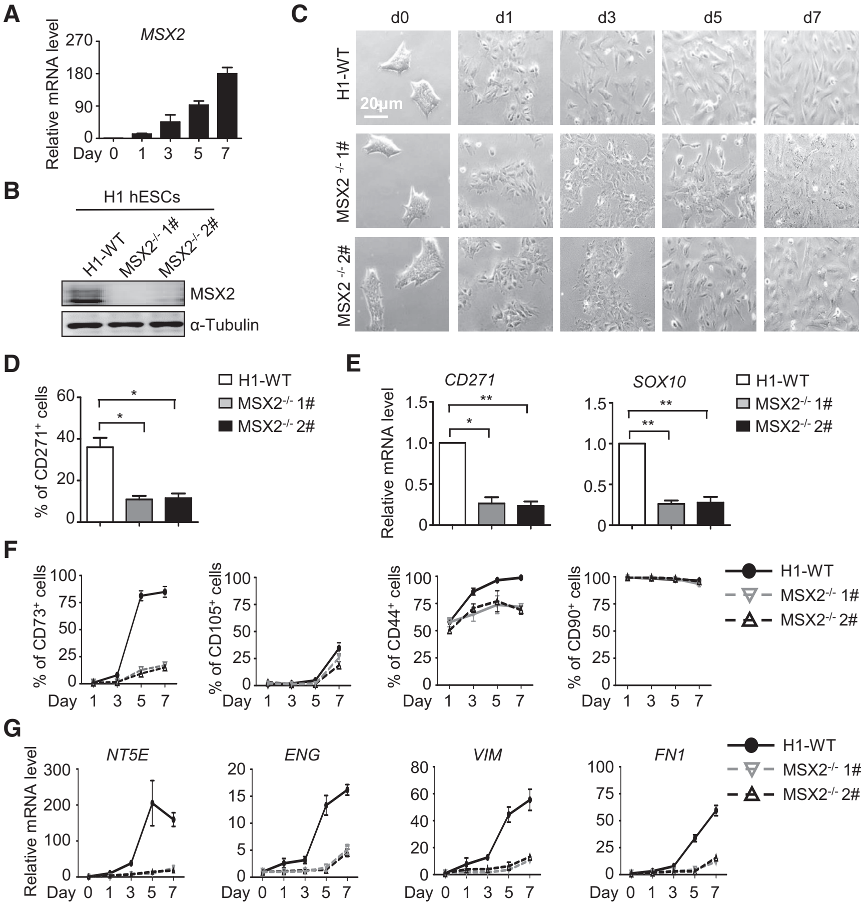

Figure 5. MSX2 Is Critical for Mesenchymal Differentiation

(A) Time course analysis of MSX2 gene expression in H1 hESCs under SMs conditions for indicated time by qRT-PCR (mean ± SEM, N = 3).

(B) Western blotting analysis confirmed the expression of MSX2 in wild-type H1 (H1-WT) and MSX2-deleted H1 hESCs after MSC induction for 7 days under SMs conditions. α-Tubulin was used as a loading control.

(C) Images of H1-WT and MSX2-deleted H1 hESCs under SMs conditions for indicated time points. Scale bar, 20 μm.

(D and E) FCM analysis (D) and qRT-PCR analysis (E) for NCC marker in H1-WT and MSX2-deleted H1 hESCs after MSC induction at day 3 under SMs conditions (mean ± SEM, N = 3). $^{*}p < 0.05$; $^{**}p < 0.01$.

(F and G) FCM analysis (F) and qRT-PCR analysis (G) for MSC markers for indicated times in H1-WT and MSX2-deleted H1 hESCs after MSC induction under SMs conditions (mean ± SEM, N = 3).

was much lower than those of neural crest-related genes, and these data implied that mesendoderm may act as another potential origin for MSC differentiation. Together, these data suggest that neural crest serves as the main intermediate cell type during MSC induction with our current methods (Figures S4D–S4H).

MSX2 Is Critical for Mesenchymal Differentiation

Because overexpression of MSX2 sufficed to initiate the MSC differentiation from hPSCs (Figures 1B–1F), we asked whether MSX2 is necessary for human mesengenesis. The use of small molecules, albeit not optimal, already enabled us to achieve consistent generation of $CD73^{+}$ and $CD105^{+}$ MSCs (Figures 2A–2C). Indeed, upregulation of endogenous MSX2 was observed in this differentiation system (Figure 5A). We next assessed the effect of MSX2 deletion in H1 hESCs (established previously; Wu et al., 2015) and BC1 hiPSCs (established herein) on mesenchymal differentiation. First, MSX2 deletion significantly delayed the morphological changes of hPSCs to MSCs (Figures 5B and S5A). On day 3, while all wild-type cells became elongated and turned into spindle-like separated cells, MSX2-deleted

(D) Heatmap illustration shows the expression changes of mesendoderm, MSC and NCC, and TB (trophoblast)-associated genes during MC-MSC induction.

(E) qRT-PCR analysis of the dynamic expression for NCC-associated genes during MSC induction from GFP-MSX2 H1 hESCs with DOX + SM (mean ± SEM, N = 3).

(F) MSC potential analysis of $CD271^{+}$ NCCs derived from GFP-MSX2 H1 hESCs. $CD271^{+}$ cells were isolated at day 3 of differentiation and cultured with DOX + SM for 96 hr, followed by FCM analysis.

(G) qRT-PCR analysis showing the depletion of SOX10 by small hairpin RNAs (shRNAs; a mixture of shSOX10-1, shSOX10-2) (mean ± SEM, N = 3). $^{**}p < 0.01$.

(H and I) FCM analysis for $CD271^{+}$ NCCs at day 3 (H) or MSC markers at day 7 (I) in H1 hESCs (Scr, shSOX10) under SMs conditions, respectively (mean ± SEM, N = 3). $^{*}p < 0.05$; $^{**}p < 0.01$; NS, not significant.

See also Figure S4.

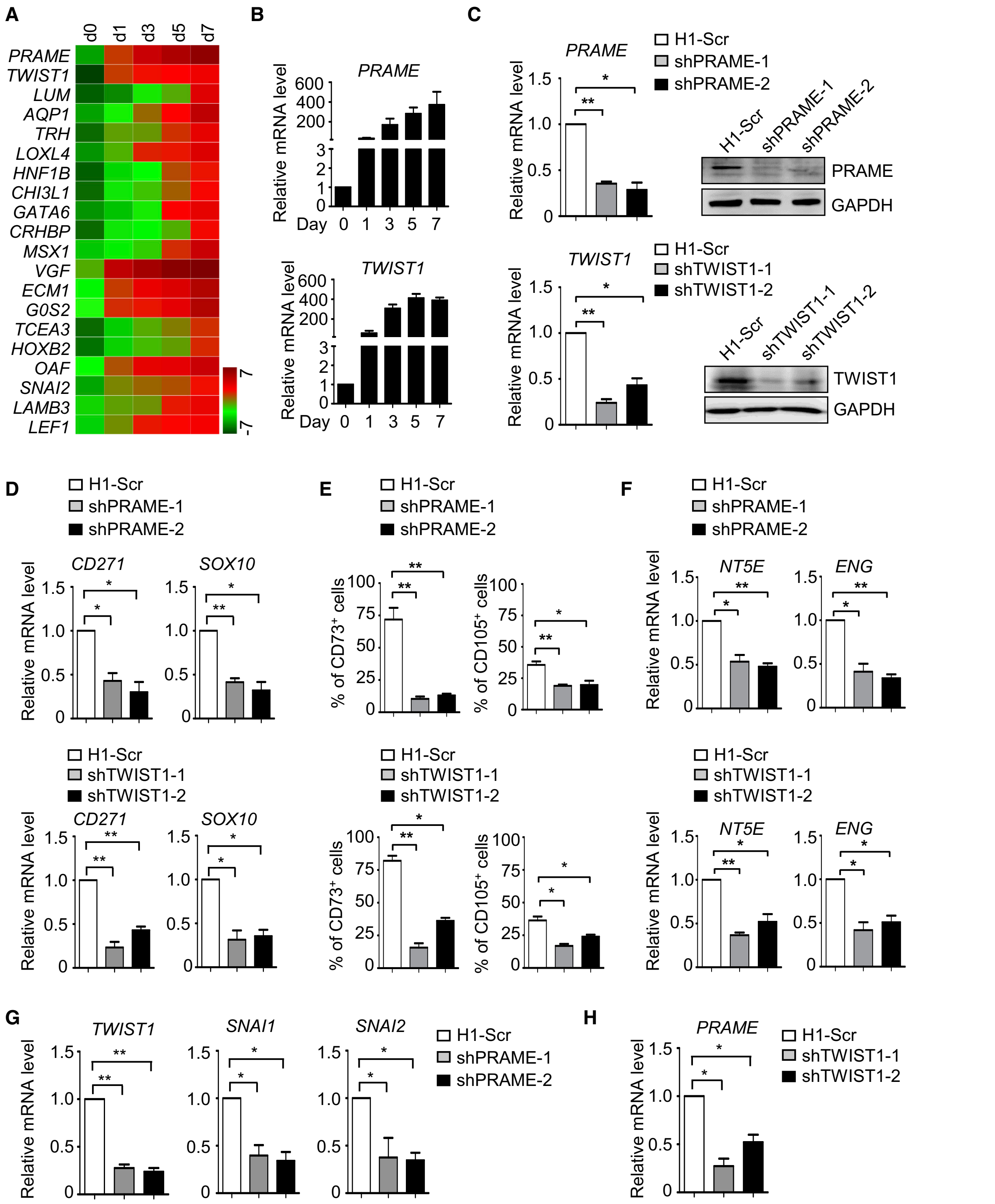

Figure 6. PRAME and TWIST1 Are Essential for MSC Generation from hPSCs

(A) Heatmap illustration shows top 20 DEGs continuously upregulated according to the fold change during hPSC-MSC induction with DOX + SMs.

(B) Time course analysis of PRAME and TWIST1 mRNA levels in GFP-MSX2 H1 hESCs during MC-MSC induction by qRT-PCR (mean ± SEM, N = 3). Values are normalized to day 0 (=1) before adding DOX.

(legend continued on next page)

cells still retained the morphology of cell aggregates (Figure 5C). Consistently, compared with the wild-type cells, neural crest cell (NCC) generation was severely impaired in MSX2-deleted cells (Figures 5D and 5E). Furthermore, much lower percentages of $CD73^+$ and $CD105^+$ cells were produced with MSX2 deletion compared with wild-type H1 hESCs and BC1 hiPSCs (Figures 5F and S5B). Consistently, expressions of NT5E, ENG, VIM, and FN1 were also severely attenuated after MSX2 deletion (Figures 5G and S5C). Thus, MSX2 is essential for MSC differentiation of hPSCs.

As mentioned above, mesendoderm and trophoblasts were also reported as the alternative origins for MSCs during hPSC differentiation. We thus also tested whether MSX2 deletion had any impact on hPSC differentiation into MSCs through mesendoderm and trophoblast by taking advantage of previously reported strategies (Tran et al., 2012; Wang et al., 2016). Indeed, we found MSX2 deletion severely decreased the expression of mesendoderm and trophoblast-associated genes (Figures S5D and S5E) and the percentage of $CD44^+$, $CD73^+$, and $CD105^+$ cells (Figures S5F and S5G). Markers of mesenchymal cells, such as NT5E, ENG, VIM, and FN1, were also found to be much lower after MSX2 depletion (Figures S5H and S5I). Thus, MSX2 is also required for hPSC differentiation into MSCs through mesendoderm and trophoblast. Altogether, these data revealed MSX2 is a general effector mediating MSC differentiation from hPSCs.

PRAME and TWIST1 Are Essential for MSC Generation of hPSCs

To further dissect the molecular mechanisms underlying human mesenchymal differentiation, we sought to identify genes essential for the differentiation process. First, we selected the top 20 genes with consecutive upregulation during differentiation (Figure 6A), allowing us to discover PRAME (preferentially expressed antigen in melanoma) (Epping et al., 2005) and TWIST1 (twist family bHLH transcription factor 1) (Qin et al., 2012), both of which exhibited robust and rapid upregulation at the early stage of MSC differentiation from hPSCs under the above optimal or sub-optimal circumstances (Figures 6B and S6A). We next asked whether they had a functional role in mesenchymal differentiation. Small hairpin RNA-mediated knockdown successfully depleted PRAME or TWIST1, as shown by decreased mRNA and protein levels (Figure 6C). Indeed, depletion of PRAME or TWIST1 severely impaired the differentiation of neural crest, as assessed by real-time PCR analysis of neural crest-associated genes CD271 and SOX10 (Figure 6D). Furthermore, the percentage of $CD44^+$, $CD73^+$, and $CD105^+$ cells (Figures 6E and S6B) and mRNA levels of NT5E, ENG, VIM, and FN1 were significantly reduced (Figures 6F and S6C). Interestingly, we also found mutual regulation between TWIST1 and PRAME during MSC induction (Figures 6G and 6H). Thus, PRAME and TWIST1 are critical for hPSC differentiation to MSCs.

MSX2 Directly Targets TWIST1 during Mesenchymal Differentiation

Having identified PRAME and TWIST1 as key regulators of hPSC mesenchymal differentiation, we asked whether they could be modulated by MSX2. We first determined whether PRAME or TWIST1 overexpression could rescue the defects caused by MSX2 knockout. We confirmed ectopic PRAME and TWIST1 expression by using western blotting or emergence of GFP fluorescence (Figures 7A, S7A, and S7B). Interestingly, overexpression of TWIST1, but not PRAME, rescued the decrease of $CD73^+$ and $CD105^+$ cells caused by MSX2 knockout (Figures 7B and S7C). qRT-PCR analysis further showed that the mRNA levels of NT5E, ENG, VIM, and FN1 were restored upon TWIST1 overexpression (Figure 7C). Thus, TWIST1 may serve as a downstream target of MSX2 during MSC differentiation of hPSCs.

To assess whether MSX2 directly targets TWIST1, we isolated the *TWIST1* 5′ flanking sequence of various lengths (0.7 and 1.4 kb) and tested their responses to MSX2 ectopic expression using a luciferase-based reporter assay. Indeed, the two *TWIST1* promoter fragments responded to MSX2 overexpression by increasing the luciferase activity (Figures 7D and S7D). The 0.7 kb fragment was used for further study because no significant difference was seen between the two fragments (Figure S7D).

(C) qRT-PCR and western blotting analysis showing the successful depletion of PRAME or TWIST1 by shRNA targeting PRAME or TWIST1 (mean ± SEM, N = 3). *p < 0.05; **p < 0.01.
(D) qRT-PCR analysis of NCC markers in H1 hESCs (Scr, shPRAME, shTWIST1) after MSC induction for 3 days under SMs conditions (mean ± SEM, N = 3). *p < 0.05; **p < 0.01.
(E) FCM analysis for MSC markers in H1 hESCs (Scr, shPRAME, shTWIST1) after MSC induction for 7 days under SMs conditions (mean ± SEM, N = 3). *p < 0.05; **p < 0.01.
(F) qRT-PCR analysis of MSC markers in H1 hESCs (Scr, shPRAME, shTWIST1) after MSC induction for 7 days under SMs conditions (mean ± SEM, N = 3). *p < 0.05; **p < 0.01.
(G and H) qRT-PCR analysis of TWIST1-associated genes or PRAME in H1 hESCs (Scr, shPRAME, shTWIST1) after MSC induction for 3 days under SMs conditions (mean ± SEM, N = 3). *p < 0.05; **p < 0.01.
See also Figure S6.

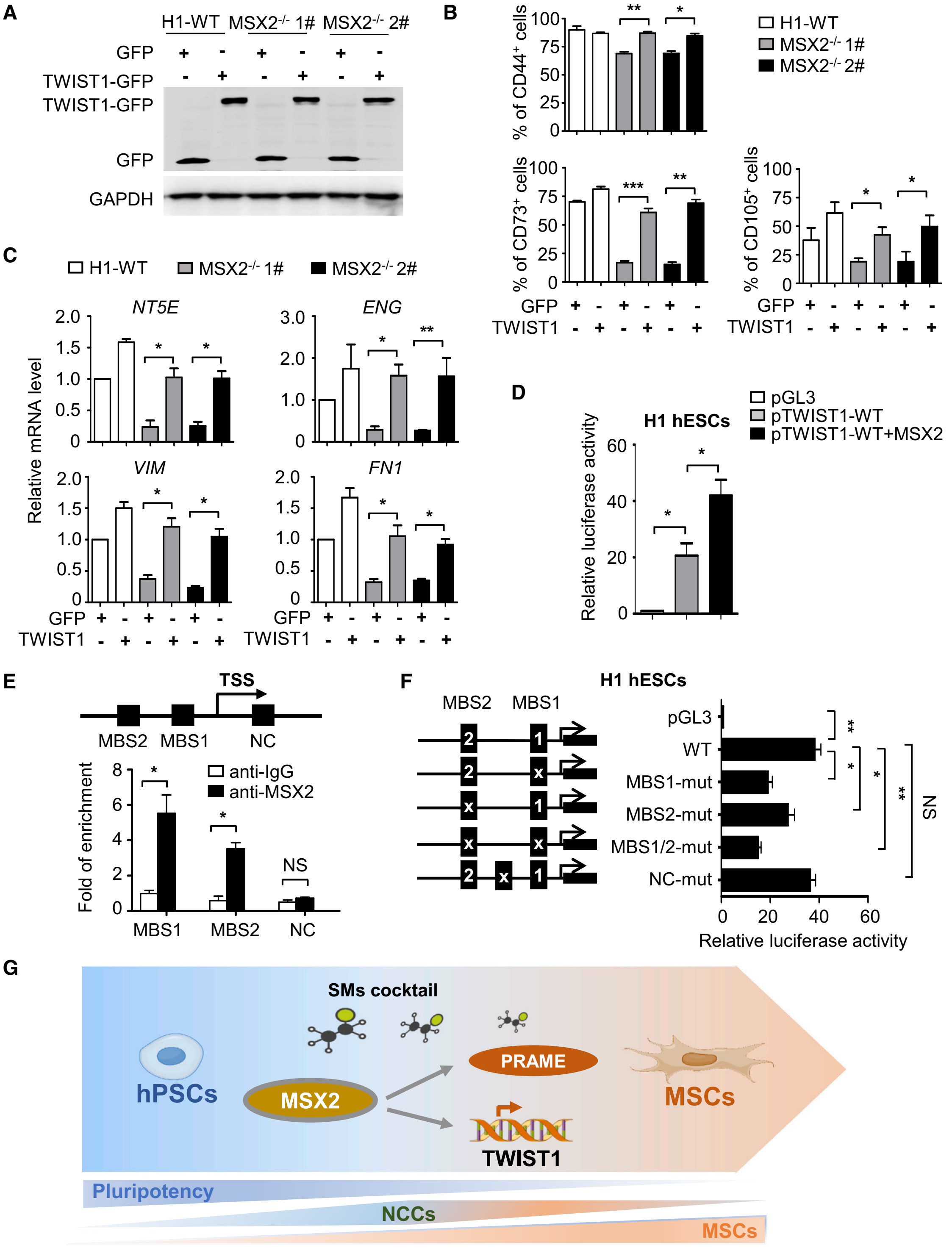

Figure 7. MSX2 Directly Targets TWIST1 during Mesenchymal Differentiation

(A) Western blotting analysis of exogenous TWIST1 or GFP in H1-WT and MSX2-deleted H1 cells at day 7 of MSC induction under SMs conditions. GAPDH was used as a loading control.

(B and C) FCM analysis (B) and qRT-PCR analysis (C) of indicated MSC markers in H1 and MSX2-deleted H1 cells without or with TWIST1 overexpression at day 7 of MSC induction under SMs conditions (mean ± SEM, N = 3). *p < 0.05; **p < 0.01; ***p < 0.001.

(legend continued on next page)

Interestingly, two potential MSX2 binding sites (MBSs), MBS1 (CCAATGAC) and MBS2 (CGAATTGT), were identified within this fragment, while chromatin immunoprecipitation (ChIP) analysis showed that the area containing either MBS1 or MBS2 could be enriched by MSX2 (Figure 7E). Further, mutations of these two sites severely impaired TWIST1 activation by MSX2 both in H1 and BC1 hPSCs (Figures 7F and S7E). Thus, the two MBSs are likely MSX2 binding sites within the TWIST1 promoter that are functional during MSC differentiation of hPSCs.

DISCUSSION

In this study, we found that MSX2 is sufficient to initiate the mesenchymal differentiation program in hPSCs. By taking advantage of MSX2 as a programming factor and addition of soluble factors, we establish a novel strategy to differentiate hPSCs into MSCs within a week without any co-culture or EB utilization. To our knowledge, this is a more rapid procedure than any has been described thus far for MSC differentiation from hPSCs. The transcriptome analysis further reveals a stepwise early developmental process of human MSCs with neural crest identified as the intermediate stage between pluripotency and mesenchymal fate. We also discovered PRAME and TWIST1 as essential regulators in mediating MSC differentiation from hPSCs (Figure 7G).

We previously demonstrated that MSX2 functions to mediate the entry of hPSCs into mesendoderm during hPSC early fate specification (Wu et al., 2015). In this study, we examined the role of MSX2 in mesenchymal differentiation and found that MSX2 is also essential. In animal models, Msx2 deletion results in profound defects in the development of multiple organs, including skull vault, tooth, hair follicle, and mammary gland (Alappat et al., 2003; Satokata et al., 2000; Wilkie et al., 2000). Mutations of MSX2 are associated with Boston-type craniosynostosis and parietal foramina in human development (Jabs et al., 1993; Wilkie et al., 2000). It has been speculated that the function of MSX2 in the development of the aforementioned organs is linked to its ability to regulate epithelial to mesenchymal transition (Richter et al., 2014; Thiery and Sleeman, 2006). Our studies on hPSC mesenchymal differentiation indicate that the function of MSX2 in mesengenesis is conserved from animals to human.

Interestingly, MSX2 itself is sufficient to initiate the mesenchymal differentiation program in hPSCs. This function is largely unknown in hPSCs, thus revealing MSX2 as a stem cell programming factor. Indeed, with the aid of a number of chemical compounds and growth factors, MSX2 programs hPSCs into functional MSCs within a week, thereby significantly accelerating MSC generation compared with previous described methods involving co-culture or EB induction (Barberi et al., 2005; Mahmood et al., 2010). MSCs generated from this system show *in vivo* function comparable with BM-MSCs (Wang et al., 2016). Since the ectopic MSX2 was delivered via lentivirus, MSCs generated using the current differentiation protocol currently cannot be used for therapeutic purposes.

By using time course genome-wide gene profiling analysis, we also discover a molecular roadmap of MSC generation from hPSCs. Interestingly, we identify neural crest as the intermediate stage occurring between pluripotency and mesenchymal fate. Different models with respect to the intermediate stage during MSC differentiation from hPSCs have been proposed previously, including mesoderm, endoderm, neural crest, or trophoblast lineage (Morikawa et al., 2009; Murray et al., 2014; Slukvin and Vodyanik, 2011; Vodyanik et al., 2010; Wang et al., 2016). With lineage tracing studies *in vivo*, it has been shown that $Sox1^+$ neuroepithelium can give rise to MSCs in part through a neural crest intermediate stage (Takashima et al., 2007). Together, these results indicate neural crest can be a physiological stage during human mesengenesis rather than a culture artificial *in vitro*. By utilizing the rapid and high-efficiency MSC differentiation model, we provide convincing evidence supporting the neural crest intermediate stage. Our previous studies revealed that

(D) Relative luciferase activity in GFP-MSX2 H1 hESCs transfected with pGL3 construct containing TWIST1 promoter (pTWIST1-0.7kb-LUC) ± DOX (3 μg/mL) for 72 hr (mean ± SEM, N = 3). *p < 0.05. Values are normalized to the pGL3 group (=1).
(E) ChIP-qPCR analysis of the occupancy of MSX2 on the two potential MSX2-binding sites (MBS1, MBS2) of TWIST1 promoter in GFP-MSX2 H1 hESCs with DOX (3 μg/mL) for 72 hr. Non-specific immunoglobulin G was used as isotype control. Values are normalized to those of their corresponding input samples (mean ± SEM, N = 3). *p < 0.05; NS, not significant.
(F) Relative luciferase activity in GFP-MSX2 H1 hESCs transfected with WT or MSX2-binding site mutated (MBS1-mut, MBS1-mut, MBS1/2-mut) TWIST1 promoter-luciferase reporter constructs with DOX (3 μg/mL) for 3 days. A non-specific mutant in TWIST1 5′ flanking region was used as a negative control (NC-mut). Normalized to the cells transfected with pGL3 (=1) (mean ± SEM, N = 3). *p < 0.05; **p < 0.01; NS, not significant. Values are normalized to the pGL3 group (=1).
(G) Schematic model for efficient hPSC-MSC induction and the underlying mechanism. Based on MSX2 and specific SMs cocktail, hPSCs can be directly programmed into MC-MSCs through an NCC intermediate stage. During the process, MSX2 upregulates the expressions of PRAME and TWIST1. Furthermore, TWIST1 serves as a key direct target of MSX2 and mediates its programming function.
See also Figure S7.

MSX2 is essential for mesendoderm induction from hPSCs. Interestingly, this study demonstrated that MSX2 induces hPSC differentiation to MSCs mainly via a neural crest intermediate. We speculate that the differences of both MSX2 induction time and culture media might lead to the different outcomes in these two studies. Thus, our studies confirm and extend previous findings, demonstrating that functional MSCs can be generated from hPSCs via the neural crest stage.

Very limited studies have been conducted to dissect the mechanism for mesenchymal differentiation from hPSCs (Deng et al., 2016; Yu et al., 2017). With the MSX2-based differentiation strategy and additional profiling analysis, we identified a large number of genes associated with MSCs generation from hPSCs, of which TWIST1 and PRAME are validated functionally as essential regulators. Our findings of TWIST1 in mesenchymal differentiation of hPSCs are consistent with its previously described role in mesenchymal development and epithelial-mesenchymal transition (Kang and Massague, 2004; Mani et al., 2008). At the mechanistic level, we found that MSX2 binds directly to the promoter of TWIST1 and activates it expression. To our knowledge, the molecule link between TWIST1 and MSX2 and the underlying regulation were largely unknown before. We also identify that PRAME, a germinal tissue-specific gene that is also expressed at high hematological malignancies and solid tumors (Epping et al., 2005; Oehler et al., 2009), is essential for mesenchymal differentiation. This is a novel function of PRAME that has never been documented. Despite the lack of modulation of PRAME by MSX2, it will be of enormous interest to further explore how PRAME controls human mesenchymal development and whether PRAME promotes carcinogenesis by giving the cells mesenchymal characteristics during cancer progression. Furthermore, we found that MSX2 is a general effector mediating MSC induction from all the three intermediate sources: neural crest, mesendoderm, and trophoblast. It will be intriguing to investigate whether TWIST1 and PRAME function as downstream targets of MSX2 during MSC induction from mesendoderm and trophoblast.

EXPERIMENTAL PROCEDURES

hPSC-MSC Differentiation

To differentiate hPSCs into MSCs, hPSCs (H1 and H9 hESCs or BC1 and Z-15 hiPSCs) were separated into single cells by using Accutase (Gibco) and seeded into 12-well plates coated with growth factor-reduced gel (Stem Cell Technologies) in E8 medium supplemented with Y27632 (10 μM) (Sigma) at a density of 1.5 × 10^4/mL. After 2 days (day 0), the medium was changed to DMEM/F12 basal media supplemented with 2% fetal bovine serum (Australia), 1% L-glutamine (Gibco) and 1% NEAA (Gibco), 4 ng/mL TGF-β1 (PeproTECH), 4 ng/mL bFGF (PeproTECH), 0.5 μM CHIR99021 (Selleck), and 20 nM DAC (Sigma) from day 0 to day 5, and then the medium was changed to 2% FBS/DMEM-F12 media containing 1% L-glutamine (Gibco), 1% NEAA (Gibco), and 20 nM DAC at day 6–7. The medium was changed every day. As to GFP-MSX2 H1 hESCs or GFP-MSX2 BC1 hiPSCs, 3 μg/mL DOX was added to induce MSX2 expression during the differentiation process. Other factors tested during the differentiation process are listed in Table S3.

Ethical Approval

hBM-MSCs or mice were used under approval of research ethics (approval no. KT2014005-EC-1 for hBM-MSCs, KT2016011-EC-1 for mice) from the Laboratory Animal Center of Institute of Hematology & Blood Diseases Hospital, Chinese Academy of Medical Sciences & Peking Union Medical College.

Mouse Model of DSS-Induced Colitis

The mouse model of acute colitis was performed as reported (Wang et al., 2016) and is described in Supplemental Experimental Procedures. All animal studies were approved (approval no. KT2016011-EC-1) by the Laboratory Animal Center of the Institute of Hematology & Blood Diseases Hospital, Chinese Academy of Medical Sciences and Peking Union Medical College (license no. SCXK & SYXK, 2005-0001, Tianjin).

RNA-Seq and Bioinformatics Analysis

Human bone marrow-derived MSCs, M-MSCs, MC-MSCs, and the cells collected at indicated times of MSC differentiation from DOX-inducible GFP-MSX2 H1 hESCs were used to prepare the RNA-seq samples. RNAs were sequenced by BGI (Shenzhen, China) as we previously described (Wu et al., 2015). Data analysis was performed as described in Supplemental Experimental Procedures. RNA-seq data are available under accession number GSE104784 and SRP055541, or in Tables S4 and S5.

Statistical Analysis

Data are shown as mean ± SEM, N = 3 independent experiments. Statistical calculations were performed using GraphPad Prism 5 software (version v5.01). $p < 0.05$ was considered statistically significant (*$p < 0.05$; **$p < 0.01$, ***$p < 0.001$; NS, not significant).

SUPPLEMENTAL INFORMATION

Supplemental Information includes Supplemental Experimental Procedures, seven figures, and five tables and can be found with this article online at https://doi.org/10.1016/j.stemcr.2018.06.019.

AUTHOR CONTRIBUTIONS

L.S.Z., H.T.W., and J.X.Z. coordinated and designed the project. L.S.Z., H.T.W., C.C.L., Q.Q.W., P.S., D.W., J.J.G., W.Z., Y.F.X., and L.H.S. performed the experiments. J.X.Z., L.S.Z., H.T.W., and D.W. analyzed the data. L.S.Z., H.T.W., and J.X.Z. wrote the manuscript. J.X.Z. designed the experiments and edited the manuscript.

ACKNOWLEDGMENTS

This work was supported by the National Basic Research Program of China (2015CB964902), National Key Research and Development

Program of China Stem Cell and Translational Research (2016YFA0102300, 2017YFA0103100, and 2017YFA0103102), CAMS Initiative for Innovative Medicine (2016-I2M-1-018, 2016-I2M-3-002, and 2017-12M-1-015), Chinese National Natural Science Foundation (81530008, 31671541, 31500949, and 81570205), the Strategic Priority Research Program of Central South University (ZLXD2017004), the Hunan Province Natural Science Foundation of China (HNSF, 2015JJ2158), Tianjin Natural Science Foundation (16JCZDJC33100), Fundamental Research Funds for the Central Research Institutes (2016ZX310184-5), PUMC Youth Fund and Fundamental Research Funds for the Central Universities (3332015128), and PUMC Graduate Innovation Fund (2014-0710-1004, 2016-0710-09). We thank Dr. Linzhao Cheng for providing BC1 hiPSCs for this study. We thank Dr Zhijian Xiao for providing Z-15 hiPSCs for this study.

Received: December 9, 2017
Revised: June 19, 2018
Accepted: June 20, 2018
Published: July 19, 2018

REFERENCES

Alappat, S., Zhang, Z.Y., and Chen, Y.P. (2003). Msx homeobox gene family and craniofacial development. Cell Res. *13*, 429–442.

Ankrum, J., and Karp, J.M. (2010). Mesenchymal stem cell therapy: two steps forward, one step back. Trends Mol. Med. *16*, 203–209.

Barberi, T., Willis, L.M., Socci, N.D., and Studer, L. (2005). Derivation of multipotent mesenchymal precursors from human embryonic stem cells. PLoS Med. *2*, e161.

Boyd, N.L., Robbins, K.R., Dhara, S.K., West, F.D., and Stice, S.L. (2009). Human embryonic stem cell-derived mesoderm-like epithelium transitions to mesenchymal progenitor cells. Tissue Eng. Part A. *15*, 1897–1907.

Brown, S.E., Tong, W., and Krebsbach, P.H. (2009). The derivation of mesenchymal stem cells from human embryonic stem cells. Cells Tissues Organs *189*, 256–260.

Chen, Y.S., Pelekanos, R.A., Ellis, R.L., Horne, R., Wolvetang, E.J., and Fisk, N.M. (2012). Small molecule mesengenic induction of human induced pluripotent stem cells to generate mesenchymal stem/stromal cells. Stem Cells Transl. Med. *1*, 83–95.

Deng, P., Zhou, C., Alvarez, R., Hong, C., and Wang, C.Y. (2016). Inhibition of IKK/NF-kappaB signaling enhances differentiation of mesenchymal stromal cells from human embryonic stem cells. Stem Cell Rep. *6*, 456–465.

Epping, M.T., Wang, L., Edel, M.J., Carlee, L., Hernandez, M., and Bernards, R. (2005). The human tumor antigen PRAME is a dominant repressor of retinoic acid receptor signaling. Cell *122*, 835–847.

Friedenstein, A.J., Petrakova, K.V., Kurolesova, A.I., and Frolova, G.P. (1968). Heterotopic of bone marrow. analysis of precursor cells for osteogenic and hematopoietic tissues. Transplantation *6*, 230–247.

Frobel, J., Hemeda, H., Lenz, M., Abagnale, G., Joussen, S., Denecke, B., Saric, T., Zenke, M., and Wagner, W. (2014). Epigenetic rejuvenation of mesenchymal stromal cells derived from induced pluripotent stem cells. Stem Cell Rep. *3*, 414–422.

Fukuta, M., Nakai, Y., Kirino, K., Nakagawa, M., Sekiguchi, K., Nagata, S., Matsumoto, Y., Yamamoto, T., Umeda, K., Heike, T., et al. (2014). Derivation of mesenchymal stromal cells from pluripotent stem cells through a neural crest lineage using small molecule compounds with defined media. PLoS One *9*, e112291.

Gammill, L.S., and Bronner-Fraser, M. (2003). Neural crest specification: migrating into genomics. Nat. Rev. Neurosci. *4*, 795–805.

Gibson, J.D., O'Sullivan, M.B., Alaee, F., Paglia, D.N., Yoshida, R., Guzzo, R.M., and Drissi, H. (2017). Regeneration of articular cartilage by human ESC-derived mesenchymal progenitors treated sequentially with BMP-2 and Wnt5a. Stem Cells Transl. Med. *6*, 40–50.

Gonzalo-Gil, E., Perez-Lorenzo, M.J., Galindo, M., Diaz de la Guardia, R., Lopez-Millan, B., Bueno, C., Menendez, P., Pablos, J.L., and Criado, G. (2016). Human embryonic stem cell-derived mesenchymal stromal cells ameliorate collagen-induced arthritis by inducing host-derived indoleamine 2,3 dioxygenase. Arthritis Res. Ther. *18*, 77.

Harkness, L., Mahmood, A., Ditzel, N., Abdallah, B.M., Nygaard, J.V., and Kassem, M. (2011). Selective isolation and differentiation of a stromal population of human embryonic stem cells with osteogenic potential. Bone *48*, 231–241.

Jabs, E.W., Muller, U., Li, X., Ma, L., Luo, W., Haworth, I.S., Klisak, I., Sparkes, R., Warman, M.L., Mulliken, J.B., et al. (1993). A mutation in the homeodomain of the human MSX2 gene in a family affected with autosomal dominant craniosynostosis. Cell *75*, 443–450.

Kang, Y., and Massague, J. (2004). Epithelial-mesenchymal transitions: twist in development and metastasis. Cell *118*, 277–279.

Keating, A. (2012). Mesenchymal stromal cells: new directions. Cell Stem Cell *10*, 709–716.

Kimbrel, E.A., Kouris, N.A., Yavanian, G.J., Chu, J., Qin, Y., Chan, A., Singh, R.P., McCurdy, D., Gordon, L., Levinson, R.D., et al. (2014). Mesenchymal stem cell population derived from human pluripotent stem cells displays potent immunomodulatory and therapeutic properties. Stem Cells Dev. *23*, 1611–1624.

Kopher, R.A., Penchev, V.R., Islam, M.S., Hill, K.L., Khosla, S., and Kaufman, D.S. (2010). Human embryonic stem cell-derived CD34+ cells function as MSC progenitor cells. Bone *47*, 718–728.

Lian, Q., Lye, E., Suan Yeo, K., Khia Way Tan, E., Salto-Tellez, M., Liu, T.M., Palanisamy, N., El Oakley, R.M., Lee, E.H., Lim, B., et al. (2007). Derivation of clinically compliant MSCs from CD105+, CD24- differentiated human ESCs. Stem Cells *25*, 425–436.

Loh, K.M., Chen, A., Koh, P.W., Deng, T.Z., Sinha, R., Tsai, J.M., Barkal, A.A., Shen, K.Y., Jain, R., Morganti, R.M., et al. (2016). Mapping the pairwise choices leading from pluripotency to human bone, heart, and other mesoderm cell types. Cell *166*, 451–467.

Lund, R.J., Narva, E., and Lahesmaa, R. (2012). Genetic and epigenetic stability of human pluripotent stem cells. Nat. Rev. Genet. *13*, 732–744.

Luzzani, C.D., and Miriuka, S.G. (2017). Pluripotent stem cells as a robust source of mesenchymal stem cells. Stem Cell Rev. *13*, 68–78.

Mahmood, A., Harkness, L., Schroder, H.D., Abdallah, B.M., and Kassem, M. (2010). Enhanced differentiation of human embryonic stem cells to mesenchymal progenitors by inhibition of TGF-beta/activin/nodal signaling using SB-431542. J. Bone Miner. Res. *25*, 1216–1233.

Mani, S.A., Guo, W., Liao, M.J., Eaton, E.N., Ayyanan, A., Zhou, A.Y., Brooks, M., Reinhard, F., Zhang, C.C., Shipitsin, M., et al. (2008). The epithelial-mesenchymal transition generates cells with properties of stem cells. Cell *133*, 704–715.

Meng, X., Su, R.J., Baylink, D.J., Neises, A., Kiroyan, J.B., Lee, W.Y., Payne, K.J., Gridley, D.S., Wang, J., Lau, K.H., et al. (2013). Rapid and efficient reprogramming of human fetal and adult blood CD34+ cells into mesenchymal stem cells with a single factor. Cell Res. *23*, 658–672.

Morikawa, S., Mabuchi, Y., Niibe, K., Suzuki, S., Nagoshi, N., Sunabori, T., Shimmura, S., Nagai, Y., Nakagawa, T., Okano, H., et al. (2009). Development of mesenchymal stem cells partially originate from the neural crest. Biochem. Biophys. Res. Commun. *379*, 1114–1119.

Motohashi, T., Aoki, H., Chiba, K., Yoshimura, N., and Kunisada, T. (2007). Multipotent cell fate of neural crest-like cells derived from embryonic stem cells. Stem Cells *25*, 402–410.

Murray, I.R., West, C.C., Hardy, W.R., James, A.W., Park, T.S., Nguyen, A., Tawonsawatruk, T., Lazzari, L., Soo, C., and Peault, B. (2014). Natural history of mesenchymal stem cells, from vessel walls to culture vessels. Cell. Mol. Life Sci. *71*, 1353–1374.

Nieto, M.A., Huang, R.Y., Jackson, R.A., and Thiery, J.P. (2016). EMT: 2016. Cell *166*, 21–45.

Nombela-Arrieta, C., Ritz, J., and Silberstein, L.E. (2011). The elusive nature and function of mesenchymal stem cells. Nat. Rev. Mol. Cell Biol. *12*, 126–131.

Oehler, V.G., Guthrie, K.A., Cummings, C.L., Sabo, K., Wood, B.L., Gooley, T., Yang, T., Epping, M.T., Shou, Y., Pogosova-Agadjanyan, E., et al. (2009). The preferentially expressed antigen in melanoma (PRAME) inhibits myeloid differentiation in normal hematopoietic and leukemic progenitor cells. Blood *114*, 3299–3308.

Olivier, E.N., Rybicki, A.C., and Bouhassira, E.E. (2006). Differentiation of human embryonic stem cells into bipotent mesenchymal stem cells. Stem Cells *24*, 1914–1922.

Qin, Q., Xu, Y., He, T., Qin, C., and Xu, J. (2012). Normal and disease-related biological functions of Twist1 and underlying molecular mechanisms. Cell Res. *22*, 90–106.

Richter, A., Valdimarsdottir, L., Hrafnkelsdottir, H.E., Runarsson, J.F., Omarsdottir, A.R., Ward-van Oostwaard, D., Mummery, C., and Valdimarsdottir, G. (2014). BMP4 promotes EMT and mesodermal commitment in human embryonic stem cells via SLUG and MSX2. Stem Cells *32*, 636–648.

Sabapathy, V., and Kumar, S. (2016). hiPSC-derived iMSCs: NextGen MSCs as an advanced therapeutically active cell resource for regenerative medicine. J. Cell. Mol. Med. *20*, 1571–1588.

Salem, H.K., and Thiemermann, C. (2010). Mesenchymal stromal cells: current understanding and clinical status. Stem Cells *28*, 585–596.

Sanchez, L., Gutierrez-Aranda, I., Ligero, G., Rubio, R., Munoz-Lopez, M., Garcia-Perez, J.L., Ramos, V., Real, P.J., Bueno, C., Rodriguez, R., et al. (2011). Enrichment of human ESC-derived multipotent mesenchymal stem cells with immunosuppressive and anti-inflammatory properties capable to protect against experimental inflammatory bowel disease. Stem Cells *29*, 251–262.

Satokata, I., Ma, L., Ohshima, H., Bei, M., Woo, I., Nishizawa, K., Maeda, T., Takano, Y., Uchiyama, M., Heaney, S., et al. (2000). Msx2 deficiency in mice causes pleiotropic defects in bone growth and ectodermal organ formation. Nat. Genet. *24*, 391–395.

Slukvin, I.I., and Vodyanik, M. (2011). Endothelial origin of mesenchymal stem cells. Cell Cycle *10*, 1370–1373.

Takashima, Y., Era, T., Nakao, K., Kondo, S., Kasuga, M., Smith, A.G., and Nishikawa, S. (2007). Neuroepithelial cells supply an initial transient wave of MSC differentiation. Cell *129*, 1377–1388.

Thiery, J.P., Acloque, H., Huang, R.Y., and Nieto, M.A. (2009). Epithelial-mesenchymal transitions in development and disease. Cell *139*, 871–890.

Thiery, J.P., and Sleeman, J.P. (2006). Complex networks orchestrate epithelial-mesenchymal transitions. Nat. Rev. Mol. Cell Biol. *7*, 131–142.

Tran, N.T., Trinh, Q.M., Lee, G.M., and Han, Y.M. (2012). Efficient differentiation of human pluripotent stem cells into mesenchymal stem cells by modulating intracellular signaling pathways in a feeder/serum-free system. Stem Cells Dev. *21*, 1165–1175.

Vodyanik, M.A., Yu, J., Zhang, X., Tian, S., Stewart, R., Thomson, J.A., and Slukvin, I.I. (2010). A mesoderm-derived precursor for mesenchymal stem and endothelial cells. Cell Stem Cell *7*, 718–729.

Wang, X., Kimbrel, E.A., Ijichi, K., Paul, D., Lazorchak, A.S., Chu, J., Kouris, N.A., Yavanian, G.J., Lu, S.J., Pachter, J.S., et al. (2014). Human ESC-derived MSCs outperform bone marrow MSCs in the treatment of an EAE model of multiple sclerosis. Stem Cell Rep. *3*, 115–130.

Wang, X., Lazorchak, A.S., Song, L., Li, E., Zhang, Z., Jiang, B., and Xu, R.H. (2016). Immune modulatory mesenchymal stem cells derived from human embryonic stem cells through a trophoblast-like stage. Stem Cells *34*, 380–391.

Wei, H., Tan, G., Manasi, Qiu, S., Kong, G., Yong, P., Koh, C., Ooi, T.H., Lim, S.Y., Wong, P., et al. (2012). One-step derivation of cardiomyocytes and mesenchymal stem cells from human pluripotent stem cells. Stem Cell Res. *9*, 87–100.

Wilkie, A.O., Tang, Z., Elanko, N., Walsh, S., Twigg, S.R., Hurst, J.A., Wall, S.A., Chrzanowska, K.H., and Maxson, R.E., Jr. (2000). Functional haploinsufficiency of the human homeobox gene MSX2 causes defects in skull ossification. Nat. Genet. *24*, 387–390.

Williams, A.R., and Hare, J.M. (2011). Mesenchymal stem cells: biology, pathophysiology, translational findings, and therapeutic implications for cardiac disease. Circ. Res. *109*, 923–940.

Wu, Q., Zhang, L., Su, P., Lei, X., Liu, X., Wang, H., Lu, L., Bai, Y., Xiong, T., Li, D., et al. (2015). MSX2 mediates entry of human pluripotent stem cells into mesendoderm by simultaneously suppressing SOX2 and activating NODAL signaling. Cell Res. *25*, 1314–1332.

Yang, Y., Liu, C., Lei, X., Wang, H., Su, P., Ru, Y., Ruan, X., Duan, E., Feng, S., Han, M., et al. (2016). Integrated biophysical and biochemical signals augment megakaryopoiesis and thrombopoiesis in a

three-dimensional rotary culture system. Stem Cells Transl. Med. *5*, 175–185.

Yu, Y., Deng, P., Yu, B., Szymanski, J.M., Aghaloo, T., Hong, C., and Wang, C.Y. (2017). Inhibition of EZH2 promotes human embryonic stem cell differentiation into mesoderm by reducing H3K27me3. Stem Cell Rep. *9*, 752–761.

Zhang, X., Yang, Y., Zhang, L., Lu, Y., Zhang, Q., Fan, D., Zhang, Y., Zhang, Y., Ye, Z., and Xiong, D. (2017). Mesenchymal stromal cells as vehicles of tetravalent bispecific Tandab (CD3/CD19) for the treatment of B cell lymphoma combined with IDO pathway inhibitor D-1-methyl-tryptophan. J. Hematol. Oncol. *10*, 56.

Zhou, J., Su, P., Li, D., Tsang, S., Duan, E., and Wang, F. (2010). High-efficiency induction of neural conversion in human ESCs and human induced pluripotent stem cells with a single chemical inhibitor of transforming growth factor beta superfamily receptors. Stem Cells *28*, 1741–1750.

Stem Cell Reports, Volume *11*

Supplemental Information

MSX2 Initiates and Accelerates Mesenchymal Stem/Stromal Cell Specification of hPSCs by Regulating TWIST1 and PRAME

Leisheng Zhang, Hongtao Wang, Cuicui Liu, Qingqing Wu, Pei Su, Dan Wu, Jiaojiao Guo, Wen Zhou, Yuanfu Xu, Lihong Shi, and Jiaxi Zhou

Figure. S1

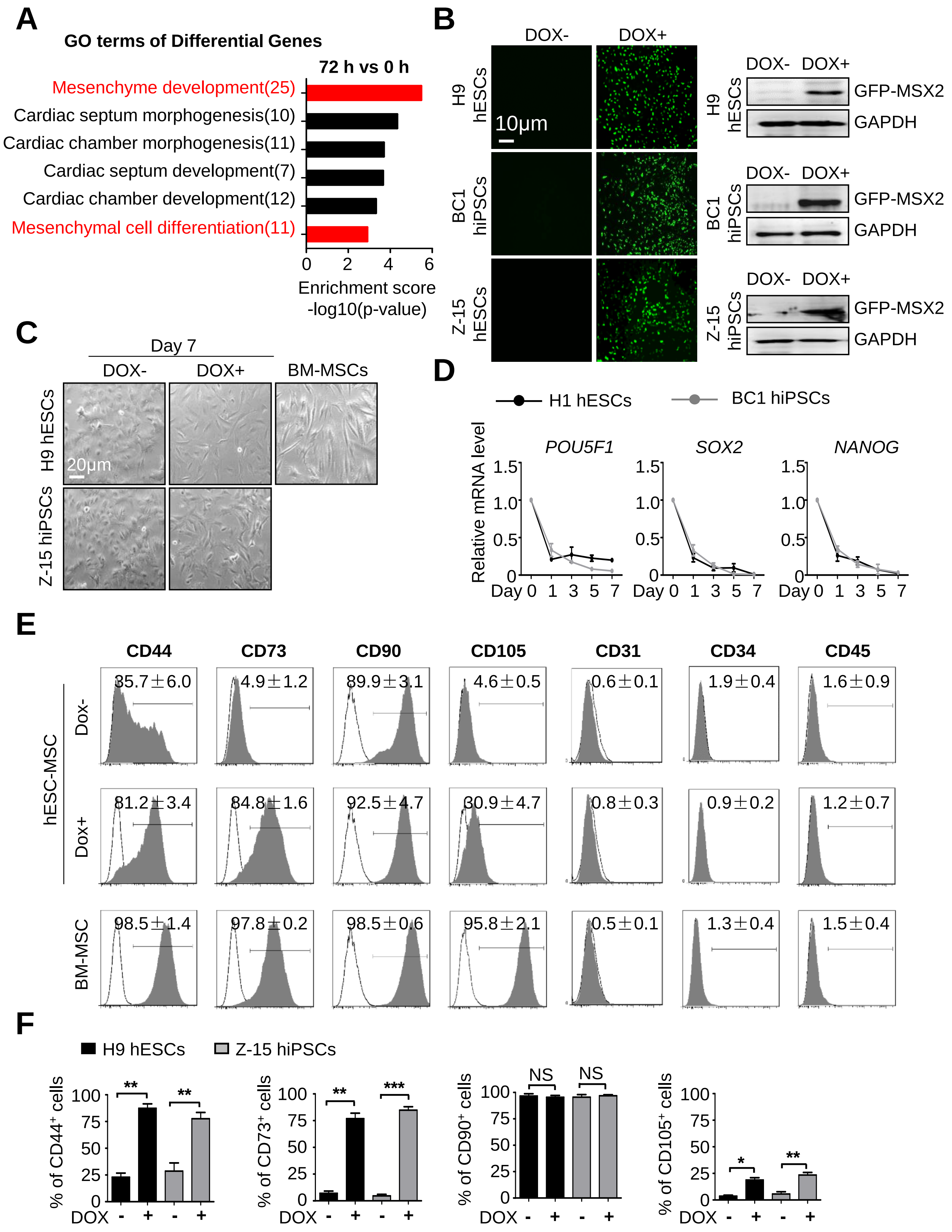

A
GO terms of Differential Genes
72 h vs 0 h
Mesenchyme development(25)
Cardiac septum morphogenesis(10)
Cardiac chamber morphogenesis(11)
Cardiac septum development(7)
Cardiac chamber development(12)
Mesenchymal cell differentiation(11)
0 2 4 6
Enrichment score
-log10(p-value)
B
DOX- DOX+
H9 hESCs
10μm
BC1 hiPSCs
Z-15 hESCs
DOX- DOX+
GFP-MSX2
GAPDH
H9 hESCs
BC1 hiPSCs
Z-15 hiPSCs
C
Day 7
DOX- DOX+ BM-MSCs
H9 hESCs
20μm
Z-15 hiPSCs
D
H1 hESCs
BC1 hiPSCs
Relative mRNA level
POU5F1
SOX2
NANOG
1.5 1.0 0.5 0
Day 0 1 3 5 7
E
CD44 CD73 CD90 CD105 CD31 CD34 CD45
hESC-MSC
Dox-
35.7±6.0 4.9±1.2 89.9±3.1 4.6±0.5 0.6±0.1 1.9±0.4 1.6±0.9
Dox+
81.2±3.4 84.8±1.6 92.5±4.7 30.9±4.7 0.8±0.3 0.9±0.2 1.2±0.7
BM-MSC
98.5±1.4 97.8±0.2 98.5±0.6 95.8±2.1 0.5±0.1 1.3±0.4 1.5±0.4
F
H9 hESCs
Z-15 hiPSCs
% of CD44+ cells
% of CD73+ cells
% of CD90+ cells
% of CD105+ cells
100 75 50 25 0
DOX - + - +
**
***
*
NS

Figure S1. Related to Figure 1. MSX2 initiates mesenchymal program in hPSCs. (A) Gene ontology analysis for up-regulated genes in DOX-inducible GFP-MSX2 H1 hESCs with 3μg/ml DOX treatment (72h vs 0h). (B) Left panel: fluorescence images of GFP-MSX2 H9 hESCs, BC1 and Z-15 hiPSCs with or without DOX (3 μg/ml) treatment for 48 h. Right panel: Western blot analysis of MSX2 in GFP-MSX2 H9 hESCs, BC1 and Z-15 hiPSCs with or without DOX (3 μg/ml) treatment for 48 h. Scale bar=10μm. (C) Phase contrast images of GFP-MSX2 H9 hESCs and Z-15 hiPSCs during differentiation in DMEM/F12 media containing 2% FBS with or without DOX (3 μg/ml) treatment. A time-course analysis is shown (from 0 h to 120 h). BM-MSCs was used as a control. Scale bar=20μm. (D) Time-course analysis of the pluripotent genes (POU5F1, SOX2 and NANOG) expression in GFP-MSX2 H1 hESCs and BC1 hiPSCs with DOX (3 μg/ml) treatment for indicated time by qRT-PCR. All values are normalized to the level (= 1) of mRNA in the cells before adding DOX (0 h). Results are shown as mean ± SEM (N=3). (E) FCM analysis for MSC markers (CD44, CD73, CD90, CD105), endothelial markers (CD31, CD34) and hematopoietic marker (CD45) of GFP-MSX2 H1 hESCs cultured in DMEM/F12 media containing 2% FBS with or without DOX (3 μg/ml) treatment for 7 days. All data are shown as mean ± SEM. (F) FCM analysis of MSC markers (CD44, CD73, CD90, CD105) of GFP-MSX2 H9 hESCs and Z-15 hiPSCs cultured in DMEM/F12 media containing 2% FBS with or without DOX (3 μg/ml) treatment for 7 days. All data are shown as mean ± SEM (N=3).

Figure. S2

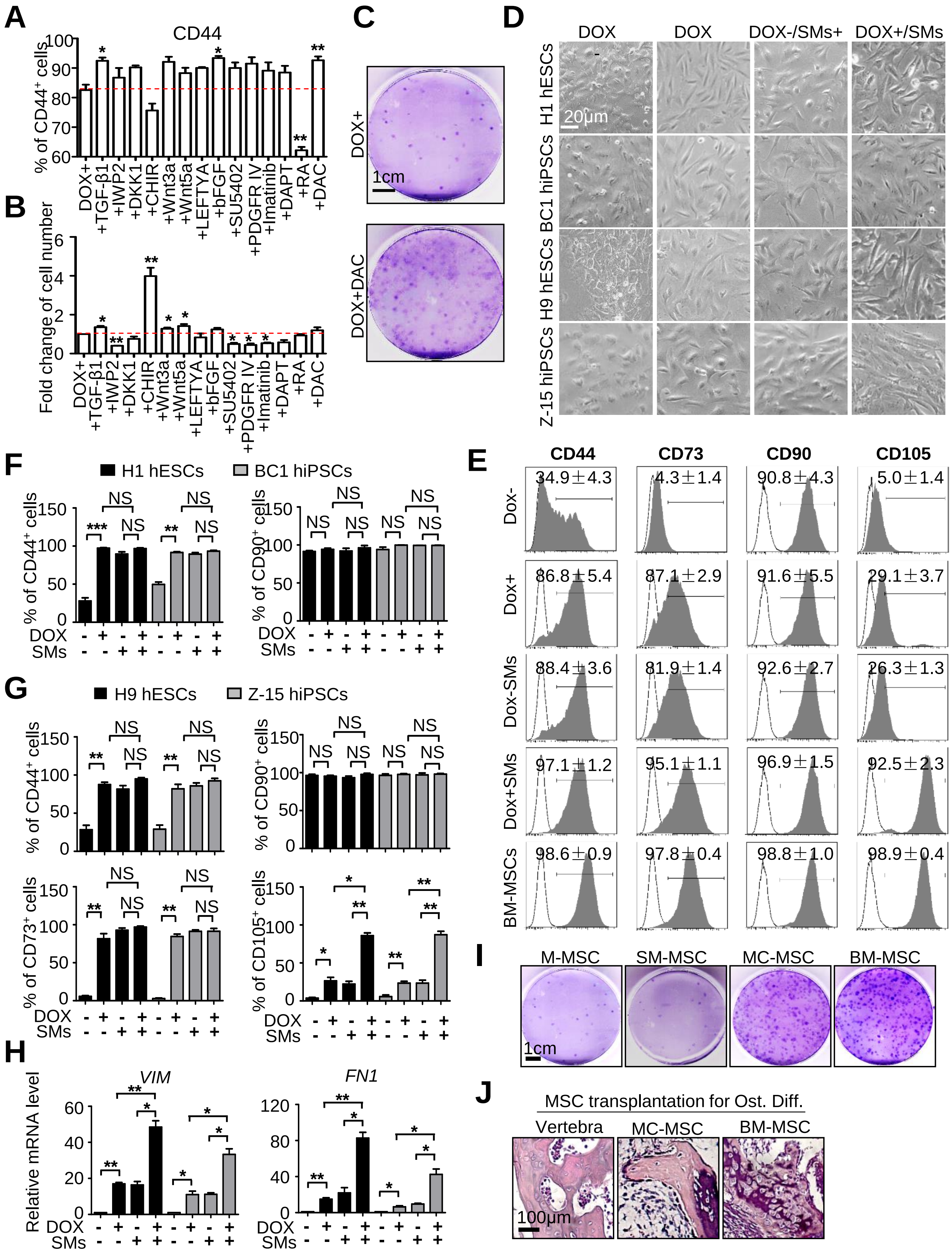
A
CD44
% of CD44+ cells
DOX+
+TGF-β1
+IWP2
+DKK1
+CHIR
+Wnt3a
+Wnt5a
+LEFTYA
+bFGF
+SU5402
+PDGFR IV
+Imatinib
+DAPT
+RA
+DAC
B
Fold change of cell number
C
DOX+
DOX+DAC
1cm
D
DOX
DOX
DOX-/SMs+
DOX+/SMs
H1 hESCs
BC1 hiPSCs
H9 hESCs
Z-15 hiPSCs
20μm
E
CD44
CD73
CD90
CD105
Dox-
Dox+
Dox-SMs
Dox+SMs
BM-MSCs
34.9±4.3
4.3±1.4
90.8±4.3
5.0±1.4
86.8±5.4
87.1±2.9
91.6±5.5
29.1±3.7
88.4±3.6
81.9±1.4
92.6±2.7
26.3±1.3
97.1±1.2
95.1±1.1
96.9±1.5
92.5±2.3
98.6±0.9
97.8±0.4
98.8±1.0
98.9±0.4
F
H1 hESCs
BC1 hiPSCs
% of CD44+ cells
% of CD90+ cells
DOX
SMs
G
H9 hESCs
Z-15 hiPSCs
% of CD44+ cells
% of CD90+ cells
% of CD73+ cells
% of CD105+ cells
DOX
SMs
H
VIM
FN1
Relative mRNA level
DOX
SMs
I
M-MSC
SM-MSC
MC-MSC
BM-MSC
1cm
J
MSC transplantation for Ost. Diff.
Vertebra
MC-MSC
BM-MSC
100μm

Figure S2. Related to Figure 2. Chemical Compounds further facilitate the MSCs generation initiated by MSX2. (A) FCM analysis for MSCs markers (CD44) of GFP-MSX2 H1 hESCs cultured in DMEM/F12 media containing 2% FBS and 3 μg/ml DOX with indicated chemical compound (see Supplemental Table S3) treatment for 7 days. IWP2 (2μM), DKK1 (200nM), CHIR (2μM), Wnt3a (10nM), Wnt5a (10nM), LEFTYA (500nM), SU5402 (2μM), PDGFR IV (5nM), Imatinib (10mg/ml), DAPT (10nM), RA (5nM). All data are shown as mean ± SEM (N=3), *, $P<0.05$; **, $P<0.01$; NS, not significant (not shown). (B) Fold change of total cell number after treatment as shown in Figure S2A. All values were normalized to the level (= 1) of the group without chemical compound treatment. All data are shown as mean ± SEM (N=3), *, $P<0.05$; **, $P<0.01$; NS, not significant. (C) 100 GFP-MSX2 H1 hESC-derived cells with DOX+ or DOX+DAC were seeded in 10 cm dish with 10%FBS/DMEM-F12 media and cultured for two weeks. The colonies were fixed and stained by crystal violet dye before being scanned. Scale bar, 1cm. (D) Phase contrast images of GFP-MSX2 hPSCs (H1 and H9 hESCs, BC1 and Z-15 hiPSCs) after treatment with DOX-, DOX+, DOX-SMs, DOX+SMs for 7 days. Scale bar=20μm. (E) FCM analysis for MSC markers (CD44, CD90, CD73 and CD105) of GFP-MSX2 H1 hESCs cultured in DMEM/F12 media containing 2% FBS with DOX-, DOX+, DOX-SMs, DOX+SMs treatment for 7 days. (F) FCM analysis for MSC markers (CD44, CD90, CD73 and CD105) in GFP-MSX2 H1 hESCs and BC1 hiPSCs cultured in DMEM/F12 media containing 2% FBS with DOX-, DOX+, DOX-SMs, DOX+SMs treatment for 7 days. All data are shown as mean ± SEM (N=3), **, $P<0.01$; ***, $P<0.001$; NS, not significant. (G) FCM analysis for CD44, CD73, CD90, CD105 expression in GFP-MSX2 H9 hESCs and Z-15 hiPSCs after treatment with DOX-, DOX+, DOX-SMs, DOX+SMs for 7 days. Results are shown as mean ± SEM (N=3). *, $P<0.05$; **, $P<0.01$; ***, $P<0.001$; NS, not significant. (H) qRT-PCR analysis of MSC markers (VIM, FN1) in GFP-MSX2 H1 hESCs and BC1 hiPSCs after treatment with indicated treatments for 7 days. All values are normalized to the level (= 1) of mRNA in the group (DOX-). Data are shown as mean±SEM (N=3). *$P<0.05$; **$P<0.01$. (I) 100 derived M-MSCs, SM-MSCs, MC-MSCs or BM-MSCs were seeded in 10 cm dish with 10%FBS/DMEM-F12 media and cultured for two weeks. The colonies were fixed and stained by crystal violet dye before being scanned. Scale bar, 1cm. (J) Bone formation in vivo by M-MSCs or MC-MSCs derived from H1 hESCs in 6-week nude mice. Vertebra of 6-week nude mice was used as a positive control. Scale Bar, 100 μm.

Figure. S3

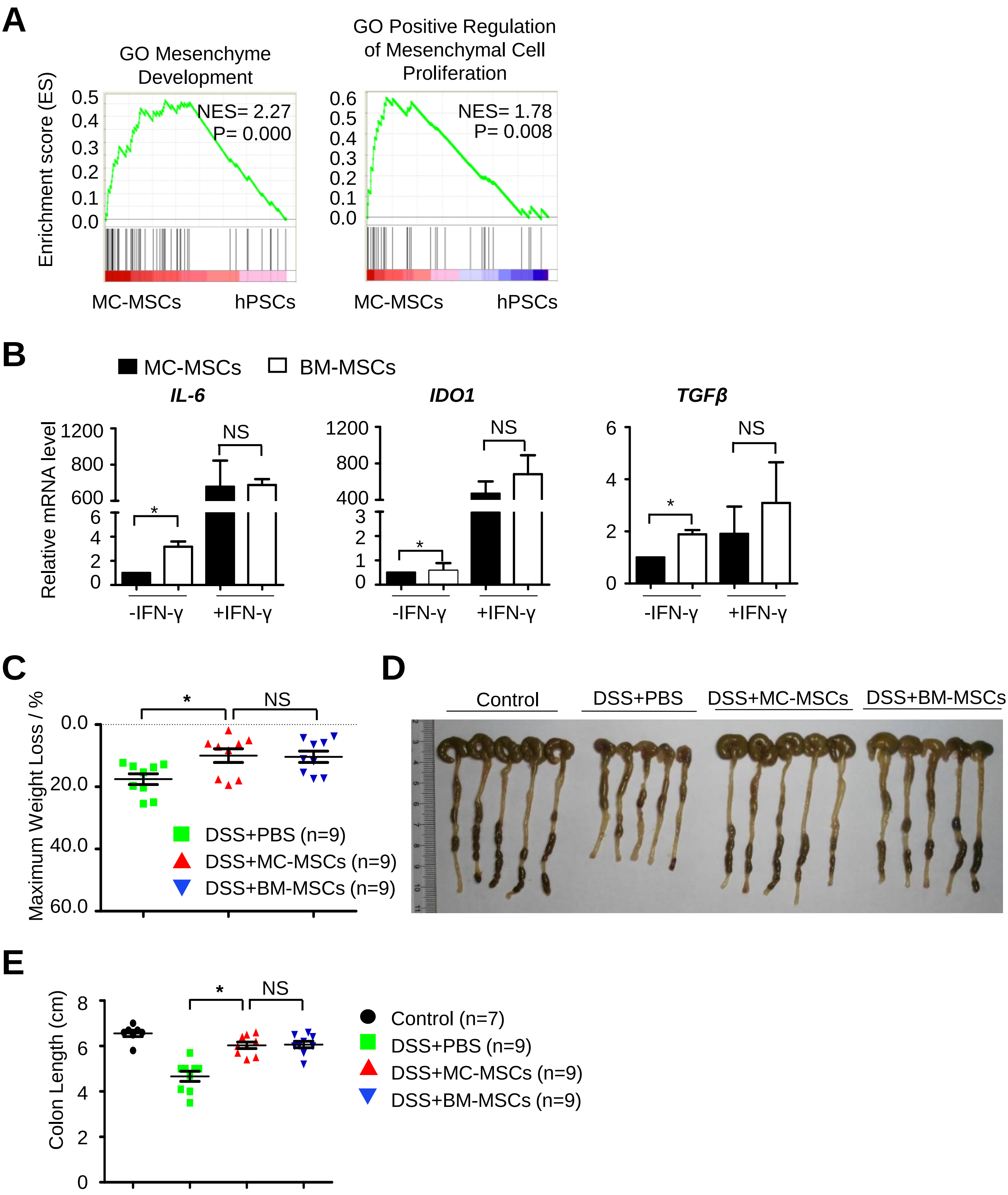

A
GO Mesenchyme Development
Enrichment score (ES)
NES= 2.27
P= 0.000
GO Positive Regulation of Mesenchymal Cell Proliferation
NES= 1.78
P= 0.008
MC-MSCs
hPSCs
B
MC-MSCs
BM-MSCs
IL-6
IDO1
TGFβ
Relative mRNA level
NS
*
-IFN-γ
+IFN-γ
C
Maximum Weight Loss / %
*
NS
DSS+PBS (n=9)
DSS+MC-MSCs (n=9)
DSS+BM-MSCs (n=9)
D
Control
DSS+PBS
DSS+MC-MSCs
DSS+BM-MSCs
E
Colon Length (cm)
*
NS
Control (n=7)
DSS+PBS (n=9)
DSS+MC-MSCs (n=9)
DSS+BM-MSCs (n=9)

Figure S3. Relative to Figure 3. MC-MSCs resemble BM-MSCs and show similar therapeutic effect on mice colitis as BM-MSCs. (A) Gene set enrichment analysis (GSEA) comparing MC-MSCs and hPSCs. The normalized enrichment scores (NES) and *P* values are showed in each plot. (B) MC-MSCs or BM-MSCs were cultured in 10% FBS/DMEM-F12 media with or without10ng/ml human IFN-γ for 24 h. The expression of IL-6, IDO1, TGFβ were measured by qRT-PCR analysis. All data are shown as means±SEM (N=3). (C) The maximum proportion of body weight loss for mice in the DSS treated groups (DSS+PBS, DSS+MC-MSCs, DSS+BM-MSCs). Results are shown as mean±SEM. All data were analyzed by students' T test, *, $P<0.05$; NS, not significant. (D) Images of colons dissected from mice in the four groups (DSS+PBS, DSS+MC-MSCs, DSS+BM-MSCs) at day 13 of the experiment. (E) The length of colons dissected from mice in the four groups at day 13 of the experiment. Data are analyzed using paired T test and shown as mean±SEM, *$P<0.001$; NS, not significant.

Figure. S4

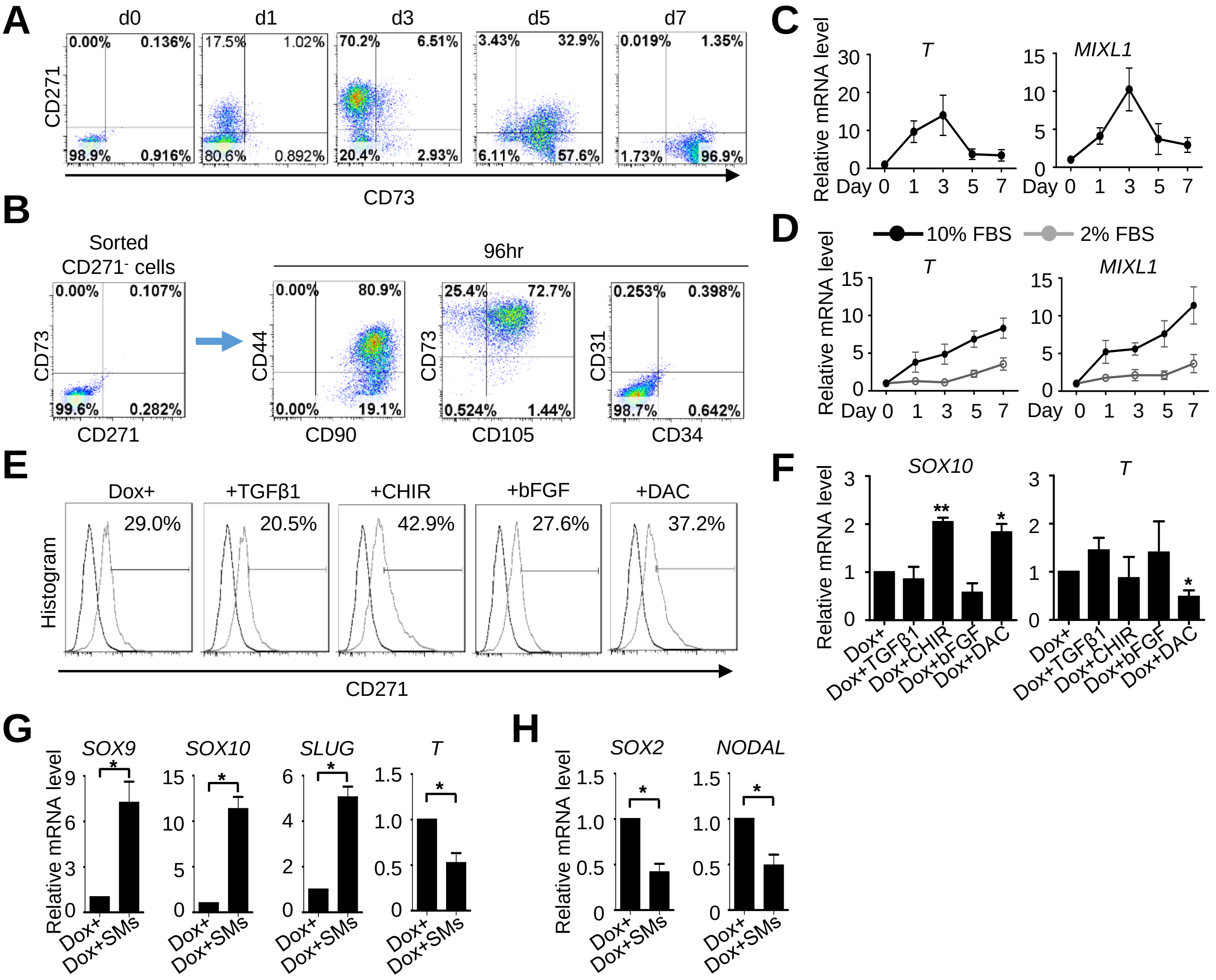
A
d0
d1
d3
d5
d7
CD271
CD73
0.00% 0.136% 98.9% 0.916%
17.5% 1.02% 80.6% 0.892%
70.2% 6.51% 20.4% 2.93%
3.43% 32.9% 6.11% 57.6%
0.019% 1.35% 1.73% 96.9%
B
Sorted CD271- cells
96hr
0.00% 0.107% 99.6% 0.282%
CD73
CD271
CD44
CD90
0.00% 80.9% 0.00% 19.1%
CD105
25.4% 72.7% 0.524% 1.44%
CD31
CD34
0.253% 0.398% 98.7% 0.642%
C
Relative mRNA level
T
MIXL1
Day 0 1 3 5 7
D
10% FBS
2% FBS
E
Dox+
+TGFβ1
+CHIR
+bFGF
+DAC
29.0%
20.5%
42.9%
27.6%
37.2%
Histogram
CD271
F
SOX10
T
Dox+
Dox+TGFβ1
Dox+CHIR
Dox+bFGF
Dox+DAC
G
SOX9
SOX10
SLUG
T
Dox+
Dox+SMs
H
SOX2
NODAL

Figure S4. Relative to Figure 4. Transcriptome analysis of DEGs during MSCs generation of hPSCs. (A) FCM analysis for the percentage of $CD271^{+}$ and $CD73^{-}$ cells(NCCs) in GFP-MSX2 H1 hESCs with DOX+SMs treatment at indicated time points of MSC induction. (B) MSC potential analysis of $CD271^{-}$ cells derived from inducible GFP-MSX2 H1 hESCs. $CD271^{-}$ cells were isolated at day3 of differentiation and cultured under DOX+SMs conditions for 96 hours, followed by FCM analysis. (C) qRT-PCR analysis of the dynamic expression for mesoendoderm associated genes (T, MIXL1) during MSC induction from DOX-inducible GFP-MSX2 overexpressing H1 hESCs under DOX+SMs conditions. All data are shown as mean ± SEM (N=3). (D) qRT-PCR analysis of the dynamic mRNA expression for mesoendoderm (T, MIXL1) associated genes in H1 hESCs under 2%, 5% FBS condition at indicated time points. All data are shown as mean ± SEM (N=3). (E) FCM analysis for the percentage of $CD271^{+}$ NCCs in GFP-MSX2 H1 hESCs with DOX+, DOX+TGFβ1, DOX+bFGF, DOX+CHIR, DOX+DAC treatment at day 3 of MSC induction. (F) qRT-PCR analysis of the expression of NCCs (SOX10) and mesoendoderm (T) associated genes in GFP-MSX2 H1 hESCs with DOX+, DOX+TGFβ1, DOX+bFGF, DOX+CHIR, DOX+DAC treatment at day 3 of MSC induction. (G) qRT-PCR analysis of the expression of NCCs (SOX9, SOX10, SLUG) and mesoendoderm (T) associated genes in GFP-MSX2 H1 hESCs with DOX+, DOX+SMs treatment at day 3 of MSC induction. (H) qRT-PCR analysis of the expression of SOX2 and NODAL in GFP-MSX2 H1 hESCs with DOX+, DOX+SMs treatment at day 3 of MSC induction.

Fig. S5

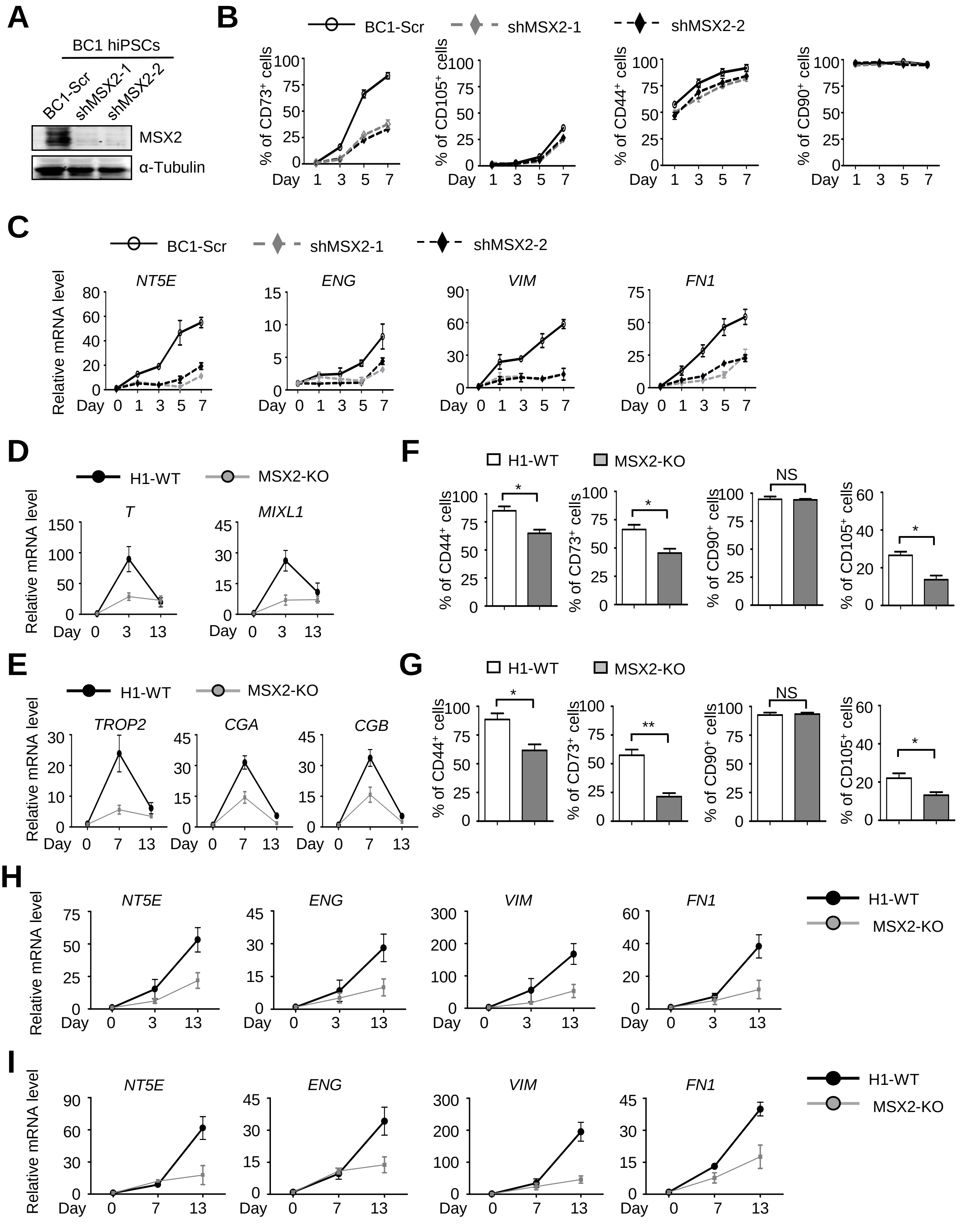
A
BC1 hiPSCs
BC1-Scr
shMSX2-1
shMSX2-2
MSX2
α-Tubulin
B
BC1-Scr
shMSX2-1
shMSX2-2
% of CD73+ cells
% of CD105+ cells
% of CD44+ cells
% of CD90+ cells
Day
C
BC1-Scr
shMSX2-1
shMSX2-2
NT5E
ENG
VIM
FN1
Relative mRNA level
Day
D
H1-WT
MSX2-KO
T
MIXL1
Relative mRNA level
Day
E
H1-WT
MSX2-KO
TROP2
CGA
CGB
Relative mRNA level
Day
F
H1-WT
MSX2-KO
% of CD44+ cells
% of CD73+ cells
% of CD90+ cells
% of CD105+ cells
NS
G
H1-WT
MSX2-KO
% of CD44+ cells
% of CD73+ cells
% of CD90+ cells
% of CD105+ cells
NS
H
NT5E
ENG
VIM
FN1
H1-WT
MSX2-KO
Relative mRNA level
Day
I
NT5E
ENG
VIM
FN1
H1-WT
MSX2-KO
Relative mRNA level
Day

Figure S5. Relative to Figure 5. MSX2 depletion impaired the neural crest, mesoderm or trophoblast derived MSCs. (A) Western-blotting analysis conformed the expression of MSX2 in BC1 hiPSCs depleted of MSX2 or expressing a scramble shRNA (Scr) after MSC induction for 7 days under SM conditions. α-Tubulin was used as a loading control. (B) FCM analysis for MSC markers (CD44, CD73, CD90, CD105) for indicated time in BC1 hiPSCs depleted of MSX2 or expressing a Scr shRNA after MSC induction under SMs conditions (mean±SEM, N=3). (C) Time-course analysis of MSC-specific markers (NT5E, ENG, VIM, FN1) in BC1 hiPSCs depleted of MSX2 or expressing a Scr shRNA after MSC induction under SMs conditions. All values are normalized to the level (= 1) of mRNA in the cells before adding DOX (0 h) (mean±SEM, N=3). (D) qRT-PCR analysis of the dynamic mRNA expression for mesoendoderm associated genes (T, MIXL1) during MSC induction from WT H1 and MSX2-deleted H1 hESCs under EB conditions. All data are shown as mean ± SEM (N=3). (E) qRT-PCR analysis of the dynamic mRNA expression for tropoblast associated genes (TROP2, CGA, CGB) during MSC induction from WT H1 and MSX2-deleted H1 hESCs under TB-MSC deriving conditions. All data are shown as mean ± SEM (N=3). (F) FCM analysis for MSC markers (CD44, CD73, CD90, CD105) in WT H1 and MSX2-deleted H1 hESCs after MSC induction under mesoendoderm-MSC deriving conditions. All data are shown as mean ± SEM (N=3). *P<0.05; NS, not significant. (G) FCM analysis for MSC markers (CD44, CD73, CD90, CD105) in WT H1 and MSX2-deleted H1 hESCs after MSC induction under TB-MSC deriving conditions. All data are shown as mean ± SEM (N=3). *P<0.05; *P<0.01; NS, not significant. (H) qRT-PCR analysis of the dynamic mRNA expression for MSC markers (NT5E, ENG, VIM, FN1) during MSC induction from WT H1 and MSX2-deleted H1 hESCs under EB conditions. All data are shown as mean ± SEM (N=3). (I) qRT-PCR analysis of the dynamic mRNA expression for MSC markers (NT5E, ENG, VIM, FN1) during MSC induction from WT H1 and MSX2-deleted H1 hESCs under TB-MSC deriving conditions. All data are shown as mean ± SEM (N=3).

Figure. S6

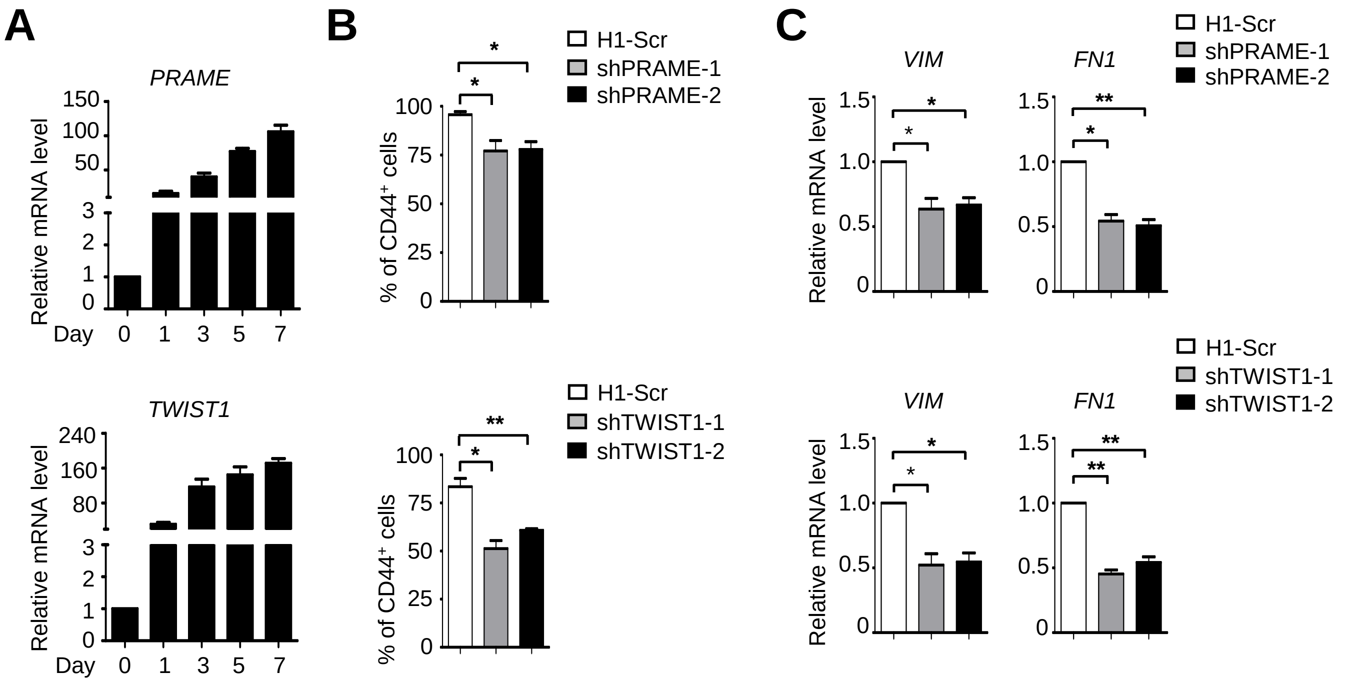
A
PRAME
Relative mRNA level
150
100
50
3
2
1
0
Day 0 1 3 5 7
TWIST1
240
160
80
B
H1-Scr
shPRAME-1
shPRAME-2
% of CD44+ cells
100
75
50
25
0
shTWIST1-1
shTWIST1-2
C
VIM
FN1
1.5
1.0
0.5
*
**

Figure S6. Relative to Figure 6. The depletion of PRAME or TWIST1 impaired MSC generation. (A) qRT-PCR analysis of the dynamic mRNA expression for PRAME or TWIST1 during MSC induction from H1 hESCs under SM conditions. All data are shown as mean ± SEM (N=3). (B) FCM analysis for MSC markers (CD44) in H1 hESCs depleted of PRAME or TWIST1 or expressing a Scr shRNA after MSC induction for 7 days under SMs conditions (mean±SEM, N=3). *P<0.05; **P<0.01. (C) qRT-PCR analysis of MSC-specific markers (VIM, FN1) in H1 hESCs depleted of PRAME or TWIST1 or expressing a Scr shRNA after MSC induction for 7 days under SM conditions (mean±SEM, N=3). *P<0.05; **P<0.01.

Fig. S7

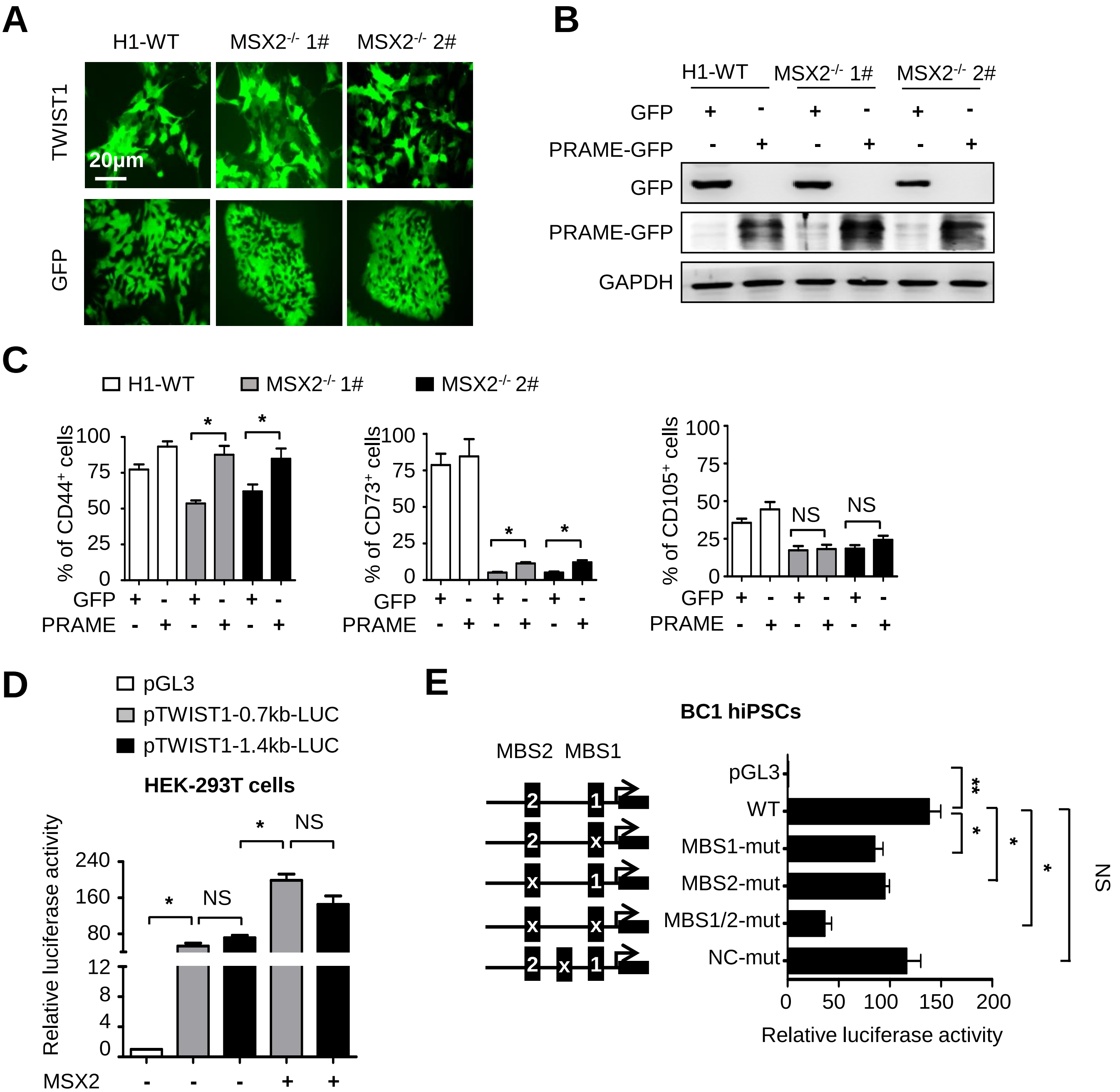
A
H1-WT
MSX2-/- 1#
MSX2-/- 2#
TWIST1
GFP
20μm
B
H1-WT
MSX2-/- 1#
MSX2-/- 2#
GFP
PRAME-GFP
GAPDH
C
H1-WT
MSX2-/- 1#
MSX2-/- 2#
% of CD44+ cells
% of CD73+ cells
% of CD105+ cells
GFP
PRAME
NS
D
pGL3
pTWIST1-0.7kb-LUC
pTWIST1-1.4kb-LUC
HEK-293T cells
Relative luciferase activity
MSX2
NS
E
BC1 hiPSCs
MBS2 MBS1
pGL3
WT
MBS1-mut
MBS2-mut
MBS1/2-mut
NC-mut
Relative luciferase activity
NS

Figure S7. Relative to Figure 7. MSX2 directly targets TWIST1 during mesenchymal differentiation. (A) Fluorescence images of WT H1 and MSX2-deleted H1 cells ($MSX2^{-/-}$ 1#; $MSX2^{-/-}$ 2#) infected with a GFP or TWIST1-GFP vector at day 7 of MSC induction under SMs conditions. Scale bar=20μm. (B) Western blotting analysis of exogenous PRAME or GFP in WT H1 and MSX2-deleted H1 cells ($MSX2^{-/-}$ 1#; $MSX2^{-/-}$ 2#) at day 7 of MSC induction under SMs conditions. GAPDH was used as a loading control. (C) FCM analysis of MSC markers (CD44, CD73, CD105) in H1 and MSX2-deleted H1 cells ($MSX2^{-/-}$ 1#; $MSX2^{-/-}$ 2#) without or with PRAME overexpression at day 7 of MSC induction under SMs conditions. All data are shown as mean ± SEM (N=3). *, $P<0.05$; NS, not significant. (D) Relative luciferase activity in HEK-293T cells co-transfected with psin-EF1α-MSX2 vector and pGL3 basic luciferase construct or pGL3 construct containing TWIST1 promoter (pTWIST1-0.7kb-LUC, pTWIST1-1.4kb-LUC). Renilla plasmid was co-transfected as an internal control. All values are normalized to the level (= 1) of the luciferase activity in cells transfected with pGL3 empty vector. All data are shown as means ± SEM (N=3), *, $P<0.05$; NS, not significant. (E) Relative luciferase activity in GFP-MSX2 BC1 hiPSCs transfected with WT or MSX2-binding site mutated (MBS1-mut, MBS1-mut, MBS1/2-mut) TWIST1 promoter-luciferase reporter constructs with DOX (3 μg/ml) treatment for 3 days. A non-specific mutant in TWIST1 5′ flanking region was used as a negative control (NC-mut). All values were normalized to the level (= 1) of the luciferase activity in cells transfected with pGL3 basic vector. Results are shown as mean ± SEM (N=3). *, P<0.05; **, P<0.001; NS, not significant.

Supplemental Tables

Table S1. Antibodies Used in This Study. Related to Fig. 1-7 and Fig. S1-S7.

Antibodies for FCM. Related to Fig. 1-7 and Fig. S1, S2, S4, S6.

Antibody	Cat. NO.	Source
Anti-human CD44-PE	550989	BD Pharmigen
Anti- human CD90-APC	328113	BioLegend
Anti- human CD73-PercP CY5.5	46-0739-42	eBioscience
Anti- human CD73-PE	550257	BD Pharmigen
Anti- human CD105-APC	323208	BioLegend
Anti- human CD31-PE	555446	BD Pharmigen
Anti- human CD34-APC	555824	BD Pharmigen
Anti- human CD45-PE	560975	BD Pharmigen
Anti- human CD271-PE	557196	BD Pharmigen
Anti- human CD271-APC	345107	BioLegend
Anti-mouse CD3e-PE	100307	BioLegend
Anti- mouse CD4	100516	BioLegend
Anti- mouse CD8a	100721	BioLegend
Anti- mouse CD8-APC	47-0081-82	eBioscience
Anti-mouse-CD3ε	100314	BioLegend
Anti-mouse-CD28	16-0281-85	eBioscience
Molecular proles Sulfate latex	A37302	Invitrogen

Antibodies for Western-blotting assay. Related to Fig. 5-7 and Fig. S1, S5, S7.

Name	Company	Catalog	Host	Dilution
MSX2	SANTA CRUZ	Sc-15396	Rabbit	1:300
TWIST1	GeneTex	GTX50821	Rabbit	1:500
PRAME	SANTA CRUZ	sc-137188	Mouse	1:1000
GFP	ProteinTech	66002-1-Ig	Mouse	1:2000
α-Tubulin	Abcam	Ab11304	Mouse	1:10000
GAPDH	Proteintech	10494-1-AP	Rabbit	1:2000

Table S2. Primers Used in This Study. Related to Fig. 1-2, 4-7 and Fig S1- S7.

qRT-PCR primer sequences. Related to Fig. 1-2, 4-7 and Fig. S1-S7.

Gene	Forward Primer	Reverse Primer
ACTIN	CTCTTCCAGCCTTCCTTCCT	AGCACTGTGTGTTGGCGTACAG
POU5F1	CTTGAATCCCGAATGGAAAGGG	GTGTATATCCCAGGGTGATCCTC
SOX2	GCCGAGTGGAAACTTTTGTCG	GGCAGCGTGTACTTATCCTTCT
NANOG	TTTGTGGGCCTGAAGAAAACT	AGGGCTGTCCTGAATAAGCAG
MSX2	TTACCACATCCCAGCTCCTC	CCTGGGTCTCTGTGAGGTTC
TWIST1	GTCCGCAGTCTTACGAGGAG	GCTTGAGGGTCTGAATCTTGCT
PRAME	AGCCTTTGACGGGAGACAC	GAGTTCTTCCGTAAATCCAGCA
RUNX2	CTCACTACCACACCTACCTG	TCAATATGGTCGCCAAACAGATTC
BGLAP	GGCGCTACCTGTATCAATGG	TCAGCCAACTCGTCACAGTC
SOX9	AATGGAGCAGCGAAATCAAC	CAGAGAGATTTAGCACACTGATC
COL2A1	TGGACGCCATGAAGGTTTTCT	TGGGAGCCAGATTGTCATCTC
PPAR-γ	GCTGGCCTCCTTGATGAATA	TGTCTTCAATGGGCTTCACA
FABP4	TATGAAAGAAGTAGGAGTGGGC	CCACCACCAGTTTATCATCCTC
ADIPOQ	TGGTCCTAAGGGAGACATCG	TGGAATTTACCAGTGGAGCC
ACAN	CCCCTGCTATTTCATCGACCC	GACACACGGCTCCACTTGAT
NT5E	GGGCGGAAGGTTCCTGTAG	GAGGAGCCATCCAGATAGACA
ENG	AGCCCCACAAGTCTTGCAG	GCTAGTGGTATATGTCACCTCGC
VIM	GAAGAGAACTTTGCCGTTGAAG	GAAGGTGACGAGCCATTT
FN1	TGACCCCTACACAGTTTCCCA	TGATTCAGACATTCGTTCCCAC
GAPDH	GAGGTGTGAGTGGGATGGTGG	GCCTGCTTCACCACCTTCTTG
SOX9	AGCGAACGCACATCAAGAC	CTGTAGGCGATCTGTTGGGG
SOX10	CCTCACAGATCGCCTACACC	CATATAGGAGAAGGCCGAGTAGA
CD271	CCTACGGCTACTACCAGGATG	CACACGGTGTTCTGCTTGT
FOXD3	GCAACTACTGGACCCTGGAC	CTGTAAGCGCCGAAGCTCT
IL-6	ACTCACCTCTTCAGAACGAATTG	CCATCTTTGGAAGGTTCAGGTTG
IDO1	GCCAGCTTCGAGAAAGAGTTG	ATCCCAGAACTAGACGTGCAA
TGFβ	GGCCAGATCCTGTCCAAGC	GTGGGTTTCCACCATTAGCAC
TROP2	GAGATTCCCCCGAAGTTCTC	AACTCCCCCAGTTCCTTGAT
CGA	TGCCCAGAATGCACGCTAC	TTGGACCTTAGTGGAGTGGGA
CGB	TACTGCCCCACCATGACCC	GGTAGTTGCACACCACCTGA
SNAI1	TCGGAAGCCTAACTACAGCGA	AGATGAGCATTGGCAGCGAG
SNAI2	CGAACTGGACACACATACAGTG	CTGAGGATCTCTGGTTGTGGT
T	CTGGGTACTCCCAATGGGG	GGTTGGAGAATTGTTCCGATGA
NODAL	CTGCTTAGAGCGGTTTCAGATG	CGAGAGGTTGGAGTAGAGCATAA
MIXL1	TTTGGCTAGGCCGGAGATTAT	GGGCTTCAGACATTTCGTTTCAG

shRNA sequences and their targets. Related to Figure 4-6 and Fig. S5-S6.

Target	shRNA	Sequence
MSX2	shRNA-1-F	CCGGCCGTTCCATAGACCTGTGCTTCTCGAGAAGCACAGGTCTATGGAACGGTTTTT
MSX2	shRNA-1-R	AATTCAAAAACCGTTCCATAGACCTGTGCTTCTCGAGAAGCACAGGTCTATGGAACGG
MSX2	shRNA-2-F	CCGGTGCAGGCAGCGTCCATATATGCTCGAGCATATATGGACGCTGCCTGCATTTTTG
MSX2	shRNA-2-R	AATTCAAAAATGCAGGCAGCGTCCATATATGCTCGAGCATATATGGACGCTGCCTGCA
TWIST1	shRNA-1-F	CCGGGCATTCTGATAGAAGTCTGAACTCGAGTTCAGACTTCTATCAGAATGCTTTTT
TWIST1	shRNA-1-R	AATTCAAAAAGCATTCTGATAGAAGTCTGAACTCGAGTTCAGACTTCTATCAGAATGC
TWIST1	shRNA-2-F	CCGGCCTGAGCAACAGCGAGGAAGACTCGAGTCTTCCTCGCTGTTGCTCAGGTTTTT
TWIST1	shRNA-2-R	AATTCAAAAACCTGAGCAACAGCGAGGAAGACTCGAGTCTTCCTCGCTGTTGCTCAGG
PRAME	shRNA-1-F	CCGGCCTGTGATGAATTGTTCTCCTCTCGAGAGGAGAACAATTCATCACAGGTTTTTG
PRAME	shRNA-1-R	AATTCAAAAACCTGTGATGAATTGTTCTCCTCTCGAGAGGAGAACAATTCATCACAGG
PRAME	shRNA-2-F	CCGGATGTTGACTTGAGGAGTTAATCTCGAGATTAACTCCTCAAGTCAACATTTTTTG
PRAME	shRNA-2-R	AATTCAAAAAATGTTGACTTGAGGAGTTAATCTCGAGATTAACTCCTCAAGTCAACA
SOX10	shRNA-1-F	CCGGCCTCATTCTTTGTCTGAGAAACTCGAGTTTCTCAGACAAAGAATGAGGTTTTTG
SOX10	shRNA-1-R	AATTCAAAAACCTCATTCTTTGTCTGAGAAACTCGAGTTTCTCAGACAAAGAATGAGG
SOX10	shRNA-2-F	CCGGGCTGCTGAACGAAAGTGACAACTCGAGTTGTCACTTTCGTTCAGCAGCTTTTTG
SOX10	shRNA-2-R	AATTCAAAAAGCTGCTGAACGAAAGTGACAACTCGAGTTGTCACTTTCGTTCAGCAGC

Vector clone primer sequences. Related to Figure 7 and Figure S7.

	Primer Sequence
pTWIST1-1.4kb-LUC-F	CCGCTCGAGCAGAAATGCTTAAAATGTTA
pTWIST1-0.7kb-LUC-F	CCGCTCGAGGTCAGACTGGGTCGTTGTAG
pTWIST1-LUC-R	CCCAAGCTTGAGGAGAGAGCAGGAGGACG
pTWIST1-MBS1-mut-LUC-F	CTCACGTCAGGTACCGACAACTGCTGCCCC
pTWIST1-MBS1-mut-LUC-R	GGGGCAGCAGTTGTCGGTACCTGACGTGAG
pTWIST1-MBS2-mut-LUC-F	GGTTTGGGAGGAACCCGGACTAGACCCCGA
pTWIST1-MBS2-mut-LUC-R	TCGGGGTCTAGTCCGGGTTCCTCCCAAACC
pTWIST1-NC-mut-LUC-F	ACGGGGGAGGGCCTGCCCTAAGCGGAAACT
pTWIST1-NC-mut-LUC-R	AGTTTCCGCTTAGGGCAGGCCCTCCCCCGT
GFP-PRAME-F	GGACTAGTGCCACCATGGACTACA<br>AGGACGACGATGACAAGGAACGAAGGCGTTTGTG
GFP-PRAME-R	CTAATTAGGCATGAAACAGG

ChIP-qPCR primer sequences. Related to Figure 7.

MBS	Forward Primer	Reverse Primer
MBS1	GGCCGCCCGGGCCAGGTCGT	CTCCGTGCAGGCGGAAAGTT
MBS2	GGCCGCCCGGGCCAGGTCGT	GGAGGAGGGACTTTTCGAAG
MBS-NC	GGCACCGTTGCCTCGCGCCC	AACGGTCCTTACCCGTGACC

Table S3. Chemical compounds. Related to Figure 1-2, 4-7 and Figure S1-S7

Reagent	Cat. NO.	Source
TGF-β1	100-21	PEPROTECH
TGF-β3	100-36E	PEPROTECH
bFGF	100-18b	PEPROTECH
CHIR99021	S2924	Selleck
SU5402	572630-500UGCN	Millipore
DKK1	120-30	PEPROTECH
IWP2	I0536	Sigma-Aldrich
Wnt3a	5036-WN-010	R&D SYSTEMS
Wnt5a	645-WN-010/CF	R&D SYSTEMS
PDGFR Inhibitor IV	521233	EMD Millipore
Activin A	120-14	PEPROTECH
LEFTYA	746-LF-025/CF	R&D SYSTEMS
SB431542	S1067	SELLECK
BMP-4	120-05	PEPROTECH
Y-27632	S1049	SELLECK
DAPT	D5942	Sigma-Aldrich
IFNγ	300-02	PEPROTECH
Imatinib	CDS022173	Sigma-Aldrich
RA	R2625	Sigma-Aldrich
DAC	A3656	Sigma-Aldrich
BIO	B1686	Sigma-Aldrich

Supplemental Experimental Procedures

Cell culture

hESC lines (H1 and H9) and hiPSC (BC1 and Z-15) were maintained in E8 Essential medium (Gibco) on Matrigel (Stem Cell Technologies) and passaged at a dilution of 1:6 every 4-6 days. The HEK-293T cells were cultured in DMEM (Invitrogen) containing 10% fetal bovine serum (FBS) (Hyclone) and were passaged every 2-3 days. hBM-MSCs were a kind gift from professor Zhongchao Han, which were derived from fresh bone marrow aspirates donated by healthy adults (range 20–56 years) under approval of research ethics (approval no. KT2014005-EC-1) as described previously(Lu et al., 2017). BM-MSCs were cultured in DMEM-F12 (Gibco) media containing 10% FBS, 1% L-Glutamine (Gibco) and 1% NEAA (Gibco). BM-MSCs were passaged every 3-5 days. BM-MSCs at passages 3-8 were used throughout the study.

Western Blotting Analysis

Western blotting analysis was performed as describe previously(Wu et al., 2015). For the detection of target genes expression in protein level, 5×10^6 were lysed with 150μl laemmli sample buffer (BioRad) and inactivated in 100℃ for 5min as we previously did. The samples were electrophoresed on a 10% SDS-PAGE gel and then transferred onto a PVDF membrane (GE Healthcare Life Sciences). The membranes were blocked using 5% nonfat milk (BD) diluted in TBST (PBS containing 0.1% Tween 20) at room temperature for 1 h before being incubated with primary antibody at 4 °C overnight. After being washed with TBST for three times (5 min each), the membranes were incubated with HRP-conjugated secondary antibody (GE Healthcare) at room temperature for 2 h. After three washing using TBST, proteins were detected using the ECL Detection Reagent (Thermo). Finally, the blots were developed by using the Super-Signal West Pico Chemiluminescent Substrate (Pierce). Antibodies used are listed in Supplemental Table S1.

Flow Cytometric Analysis

Differentiated cells were dissociated into single cells using 0.05% Trypsin/EDTA and the cells were stained with the indicated antibodies (listed in Supplemental Table S1) in 0.2% BSA. After incubation for 30 min at room temperature in dark, the cells were washed twice with cold PBS and analyzed using FACS Canto II (BD Biosciences). 7-aminoactinomycin D (7AAD) staining was used for dead cells

exclusion. Data were analyzed with FlowJo software (Tree Star, Ashland, OR). Antibodies used are listed in Supplemental Table S1.

qRT-PCR

Total RNA was harvest with TRIzol reagent (Thermo) according to the manufacturer's instruction and quantified by using NanoDrop 2000 (Thermo). cDNA was synthesized by reverse transcriptase kit (Promaga). qRT-PCR assay was performed with SYBR Green (Qiagen) using the ABI PRISM 7900 (Applied Biosystems). The primer sequences are listed in Supplemental Table S2.

Standard Colony Forming Assay (CFU-F) of MSCs

Standard Colony Forming Assay (CFU-F) of MSCs was performed as previously reported(Meng et al., 2013). Briefly, hBM-MSCs or MSCs derived from hPSCs were detached with 0.05% trypsin and seeded at a density of 100 cells per 10cm dish and cultured in DMEM-F12 (Gbico) supplemented with 10% FBS, 1% L-Glutamine (Gibco) and 1% NEAA (Gibco), 0.5μM CHIR99021 (Selleck). 2 weeks later, the colonies were washed with 1×PBS, fixed with 4% PFA (Gbico) before staining with 0.2% Crystal Violet Solution (Solarbio). The colonies in 10 cm dish was scanned (Canon).

Tri-lineage Differentiation of MSCs

MSCs derived from hPSCs and BM-MSCs were used for adipogenic, osteogenic, and chondrogenic differentiation potential analyses were performed as described previously (Vodyanik et al., 2010; Zhang et al., 2017). All qRT-PCR primer sequences are available in Supplemental Table S2.

For adipogenic differentiation, MSCs derived from hPSCs or BM-MSCs were cultured in adipogenic differentiation medium, which contains IMDM (Gibco), 10% FBS (Hyclone), 1mM Dexamethasone (Sigma), 500μM IBMX (Sigma), 10μg/ml Insulin, 60μM Indomethacin and 2mM L-Glutamine (Gibco). After 3 weeks of culture with medium changed every 3 days, the cells were analyzed using Oil Red O staining and qRT-PCR.

For osteogenic differentiation, MSCs derived from hPSCs or BM-MSCs were maintained in osteogenic differentiation medium containing IMDM (Gibco), 10% FBS (Hyclone), 100nM Dexamethasone (Sigma), 50μg/ml Ascorbic acid (Sigma), 10μM β-Glycerophosphate (Sigma), 2mM L-Glutamine (Gibco) and 1× Nonessential amino acids (Gibco). The medium was changed every 3 days. After 3

weeks of culture, the cells were subjected to Von Kossa staining and harvested for qRT-PCR analysis. For chondrogenic differentiation, MSCs derived from hPSCs or BM-MSCs were cultured in chondrogenic differentiation medium consisting of IMDM (Gibco) supplemented with 10% FBS (Hyclone), 100nM Dexamethasone (Sigma), 50μg/ml Ascorbic acid (Sigma), 40μg/ml Proline, 10ng/ml TGF-β3, 100μg/ml Sodium Pyruvate, 2mM Insulin-Transfer-S (Gibco), 53.5μg/ml Lindeic acid, Glycerophosphate (Sigma) and 12.5μg/ml BSA (Sigma). The medium was changed every 3 days. After 3 weeks, the cells were used for Alcian Blue staining assay and collected for qRT-PCR analysis.

Transplantation of MSCs into Immunocompromised Mice

The mouse model of *in vivo* bone formation by MSCs was performed as previously reported(Deng et al., 2016) with minor modification(Deng et al., 2016). All animal studies were approved (approval no. KT2016011-EC-1) by Laboratory Animal Center of Institute of Hematology & Blood Diseases Hospital, Chinese Academy of Medical Sciences & Peking Union Medical College (license no. SCXK & SYXK 2005-0001, Tianjin). Briefly, 2×10^6 hBM-MSCs or MC-MSCs derived from DOX-inducible GFP-MSX2 overexpressing H1 hESCs under DOX+SMs conditions at day 7 of hPSC-MSCs were mixed with hydroxyapatite/tricalcium phosphate (HA/TCP) scaffolds. Then, the hBM-MSCs and MC-MSCs were transplanted into the left and right dorsal subcutaneous sites of 6-week nude mice, respectively. 8 weeks later, all of the mice were euthanized and the transplants of each mouse were collected and fixed with 4% formaldehyde for 48h. The formed bone *in vivo* was identified by haematoxylin and eosin (H&E) staining and photographed by inverted microscope (Nikon).

Population Doublings Assay

MSCs derived from hPSCs and BM-MSCs were serially passaged in six-well plates for 10 passages. Briefly, cells cultured in six-well plates were trypsinized and re-cultured in a new six-well plate at 1.0×10^4 cells/cm^2 when they reached 90% confluency. Population doublings (PD) was calculated as follows(Meng et al., 2013; Umeda et al., 2015; Wei et al., 2012): NPD= (log10 [Np] - log10[Ns])/log10, where Np represents the harvested cell number before passage and Ns represents the seeded cell number at each passage, respectively.

Inflammatory Response of MSCs to IFNγ *in vitro*

To evaluate the inflammatory response of MSCs to inflammatory factor IFNγ, MC-MSCs or hBM-MSCs were detached with 0.05% trypsin and cultured in 24-well plate with DMEM-F12 (Gbico) containing 10% FBS, 1% L-Glutamine (Gibco) and 1% NEAA (Gibco). After being treated with 10ng/ml human IFN-γ (PeproTech) for 24h, the cells were collected for RNA isolation with TRIzol reagent (ThermoFisher). The expression level of inflammatory factors was detected by Real-time PCR with the SYBR reagent (Life) and 7900HT Fast Real-time PCR System (ThermoFisher). Primers for detection of IL-6, IDO1 and TGFβ were synthetized (Invitrogen) and listed in Table S2.

In Vitro Assays of T Cell Proliferation

The inhibition analysis of T lymphocyte proliferation by MSCs *in vitro* was performed as previously described(Wang et al., 2016) with minor modification. T lymphocytes were isolated from spleen and mesenteric lymph nodes of 6-week male C57 BL/6 mice. Briefly, the total cells were labelled with PE-CD3e (BioLegend) and T lymphocyte were enriched by fluorescence-activated cell sorter (BD FACSAria IIIu). Then, the sorted $CD3^+$ T lymphocytes were labeled with 5-μM FITC-CFSE (Invitrogen) 2×10^4 MC-MSC or BM-MSCs were mixed with 1×10^5 CFSE labelled $CD3^+$ T cells per well in a 96-well plateand cultured in 1640 basal medium (Gibco) supplemented with 10% FBS (Austria) at 100 μl/well.. For $CD4^+$ T lymphocyte proliferation analysis, the $CD3^+$ T cells were stimulated with plate-bound 1 μg/ml anti-CD3 antibody (BD Bioscience) and 2 μg/ml anti-CD28 antibody (eBioscience). For $CD8^+$ T lymphocyte proliferation analysis, the $CD3^+$ T cells were stimulated with molecular probes sulfate latex (Invitrogen). After stimulation for 72 h, the cells were harvested and stained with anti-CD4 and Anti-CD8 antibodies (BD Bioscience). The $CD4^+$ or $CD8^+$ population was gated for CFSE dilution analysis.

Mouse Model of Dextran Sulfate Sodium (DSS)-induced Colitis

The DSS-induced colitis model was performed as previously described(Wang et al., 2016). Briefly, 8-week male C57BL/6 mice were administered with drinking water containing 2% DSS (BioWest) for 6 days. 1×10^6 human BM-MSCs or MC-MSCs derived from hPSCs in PBS or PBS alone (a vehicle control) were injected i.p. into animals on day 2 and 3 after the start of the DSS treatment. The mice untreated drinking water were used as control. Body weight of the mice was monitored every day from

day 0 to day 13. The stool consistency, rectal bleeding and body weight (Wang et al., 2016) of mice were used to evaluate the severity of colitis as follows: 0, normal; 1, soft; 2, soft but formed; 3, liquid. All of the mice were euthanized at day 13. Upon necropsy, the colon of each mouse was dissected from each mouse and its length was measured. After being flushed with 1 × DPBS, the colons were fixed with 4% formaldehyde for at least 48h. The distal part of the colon was used for haematoxylin and eosin (H&E) staining.

MSC Potential Analysis of CD271$^+$ NCCs or CD271$^-$ cells from hPSCs

To evaluate the MSC potential of NCCs, the isolated CD271$^+$ NCCs or CD271$^+$ cells at day 3 of MSC induction were seeded into 12-well plates coated with growth factor-reduced gel (Stem Cell Technologies) at a density of 2×10^4/ml and cultured in DMEM/F12 media supplemented with 2% FBS, 4 ng/ml TGFβ1, 4 ng/ml bFGF, 0.5 μM CHIR99021 and 20 nM Decitabine) and 3 μg/ml DOX for 4 days. Then, the cells were dissociated into single cells using 0.05% Trypsin/EDTA and stained with the indicated antibodies (listed in Supplemental Table S1) in 0.2% BSA for FCM analysis.

Differentiation of hPSCs into Mesoendoderm-derived MSCs

Differentiation of hPSCs into mesoendoderm-derived MSCs was performed as previously reported(Tran et al., 2012) with minor modification. Briefly, to induce mesoendoderm, hPSCs clumps were seeded onto GFR-coated dishes in Custom TeSR (Stem Cell Tech) containing 5ng/ml Activin A (Peprotech), 2μM BIO (Sigma) and 20ng/ml BMP4 (Peprotech) for 3 days. Then, the derived cells were further induced into MSCs with MSC-induction medium containing 90% α-MEM (Gibco), 10% FBS (Hyclone), 10ng/ml EGF (Peprotech) and 10ng/ml bFGF (Peperotech) for 10 days. Finally, the cells were dissociated into single cells using 0.05% Trypsin/EDTA and stained with the indicated antibodies (listed in Supplemental Table S1) in 0.2% BSA for FCM analysis.

Differentiation of hPSCs into Trophoblast-derived MSCs (TB-MSCs)

Differentiation of hPSCs into mesoderm-derived MSCs was performed as previously reported(Wang et al., 2016) with minor modification. Briefly, for trophoblast (TB) induction, hPSCs clumps were seeded onto GFR-coated dishes in Custom TeSR (Stem Cell Tech) containing 10μM SB431542 (Selleck) and 10ng/ml BMP4 (Peprotech) for 7 days. Then, the derived cells were further induced into MSCs with 80%

α-MEM (Gibco), 20% FBS (Hyclone), 1×NEAA(Gibco) and 1% L-Glutamine (Gibco) for 6 days. Finally, the cells were dissociated into single cells using 0.05% Trypsin/EDTA and stained with the indicated antibodies (listed in Supplemental Table S1) in 0.2% BSA for FCM analysis.

Bioinformatics Analysis

Heatmap was generated using HemI heatmap illustrator software (GPS) or R language. Gene ontology (GO) was conducted using the online tool (http://geneontology.org/ or http://david.abcc.ncifcrf.gov/). Pathway enrichment analysis was performed using Ingenuity Pathway Analysis (IPA 1.0 version) software or KEGG website (http://www.kegg.jp/). Principal component analysis (PCA) were completed in ClustVis website (http://biit.cs.ut.ee/clustvis/). The Venn map was generated in Venny 2.1 website (http://bioinfogp.cnb.csic.es/tools/venny/index.html).

Lentivirus Production

For knockdown of gene expression, shRNAs targeting MSX2, TWIST1, PRAME and SOX10 were cloned into pLKO.1 vector (Addgene), while a scramble shRNA (Scr) was used as a control. For overexpression of genes, the coding sequence of PRAME or TWIST1 was inserted into psin-EF1a-GFP vector. The lentivirus was packed and collected as described previously(Wu et al., 2015). The sequences of shRNAs are listed in Supplemental Table S2.

Construct Generation

For sgRNA-mediated MSX2 knockout by CRISPR/Cas9 system, lentivirus-based CRISPR-Cas9-Lenti-V2 vector containing sgRNAs designed with the CRISPR design website (http://tools.genome-engineering.org) were used.

For shRNA-mediated MSX2, TWIST1, PRAME and SOX10 knock-down, lentivirus-based pLKO.1 vectors (Addgene) containing various shRNA target sequences (Sigma-Aldrich) (See Supplemental Table S2) were used to package virus according to the manufacturer's instructions (Viralpower Lentivirus Packaging System, Invitrogen).

Construct carrying PRAME were generated from the coding sequence of PRAME (from cDNA of H1 hESC-derived MSC-like cells by exogenous MSX2 overexpression for 7 days). The coding sequence was finally cloned into SpeI and BmtI double digested psin-EF1a-GFP vector for further

experiment.

The reporter constructs of pTWIST1-0.7kb-LUC and pTWIST1-1.4kb-LUC were cloned from H1 hESC genomic DNA, then cloned into Xho I and Hind III double digested pGL3 vector (Promega). The two MSX2 binding sites (MBS), MBS1 (CCAATGAC) and MBS2 (CGAATTGT) on pTWIST1-0.7kb-LUC construct were predicted by JASPAR website (http://jaspar.genereg.net/cgi-bin/jaspar_db.pl). The pTWIST1-0.7kb-LUC construct containing MSX2 binding-site (MBS) mutants were generated by recombinant PCR. Primer sequences used for construct generation are provided in Supplemental Table S2.

Reporter Assay

For MSX2 target analysis, HEK-293T cells, H1 hESCs or BC1 hiPSCs were co-transfected with 0.5μg of pTWIST1-LUC reporter construct and 0.5μg of psin-EF1a-MSX2 using FuGene HD transfection reagent (Promega). 0.05μg of Renilla plasmid was co-transfected as an internal control. 48 h or 72 h after transfection, cells were harvested for luciferase activity measurement. Luciferase activity was determined using Dual-Luciferase assay (Promega) according to the Manufacturer's Instruction. All assays were carried out in triplicate. Details for the construction of pTWIST1-LUC, MSX2 binding site mutants are available in Supplemental Table S2.

Chromatin immunoprecipitation (ChIP) assay

3×10^7 GFP-MSX2 H1 hESCs were collected for ChIP assay after treatment with treated with DOX (3μg/ml) for 7 days. ChIP assay was performed using the Magna ChIP™ A/G kit (Millipore) following the Manufacturer's Instructions. The primers designed to detect the enrichment of the fragments are available in Supplemental Information, Supplemental Table S2.

Supplemental References

Deng, P., Zhou, C., Alvarez, R., Hong, C., and Wang, C.Y. (2016). Inhibition of IKK/NF-kappaB Signaling Enhances Differentiation of Mesenchymal Stromal Cells from Human Embryonic Stem Cells. Stem cell reports *6*, 456-465.

Lu, S., Ge, M., Zheng, Y., Li, J., Feng, X., Feng, S., Huang, J., Feng, Y., Yang, D., Shi, J., *et al.* (2017). CD106 is a novel mediator of bone marrow mesenchymal stem cells via NF-kappaB in the bone marrow failure of acquired aplastic anemia. Stem cell research & therapy *8*, 178.

Meng, X., Su, R.J., Baylink, D.J., Neises, A., Kiroyan, J.B., Lee, W.Y., Payne, K.J., Gridley, D.S., Wang, J., Lau, K.H., *et al.* (2013). Rapid and efficient reprogramming of human fetal and adult blood CD34+ cells into mesenchymal stem cells with a single factor. Cell research *23*, 658-672.

Tran, N.T., Trinh, Q.M., Lee, G.M., and Han, Y.M. (2012). Efficient differentiation of human pluripotent stem cells into mesenchymal stem cells by modulating intracellular signaling pathways in a feeder/serum-free system. Stem cells and development *21*, 1165-1175.

Umeda, K., Oda, H., Yan, Q., Matthias, N., Zhao, J., Davis, B.R., and Nakayama, N. (2015). Long-term expandable SOX9+ chondrogenic ectomesenchymal cells from human pluripotent stem cells. Stem cell reports *4*, 712-726.

Vodyanik, M.A., Yu, J., Zhang, X., Tian, S., Stewart, R., Thomson, J.A., and Slukvin, II (2010). A mesoderm-derived precursor for mesenchymal stem and endothelial cells. Cell stem cell *7*, 718-729.

Wang, X., Lazorchak, A.S., Song, L., Li, E., Zhang, Z., Jiang, B., and Xu, R.H. (2016). Immune modulatory mesenchymal stem cells derived from human embryonic stem cells through a trophoblast-like stage. Stem cells *34*, 380-391.

Wei, H., Tan, G., Manasi, Qiu, S., Kong, G., Yong, P., Koh, C., Ooi, T.H., Lim, S.Y., Wong, P., *et al.* (2012). One-step derivation of cardiomyocytes and mesenchymal stem cells from human pluripotent stem cells. Stem cell research *9*, 87-100.

Wu, Q., Zhang, L., Su, P., Lei, X., Liu, X., Wang, H., Lu, L., Bai, Y., Xiong, T., Li, D., *et al.* (2015). MSX2 mediates entry of human pluripotent stem cells into mesendoderm by simultaneously suppressing SOX2 and activating NODAL signaling. Cell research *25*, 1314-1332.

Zhang, X., Yang, Y., Zhang, L., Lu, Y., Zhang, Q., Fan, D., Zhang, Y., Zhang, Y., Ye, Z., and Xiong, D. (2017). Mesenchymal stromal cells as vehicles of tetravalent bispecific Tandab (CD3/CD19) for the treatment of B cell lymphoma combined with IDO pathway inhibitor D-1-methyl-tryptophan. Journal of hematology & oncology *10*, 56.
